# Dynamic Evolution of Retroviral Envelope Genes in Egg-Laying Mammalian Genomes

**DOI:** 10.1093/molbev/msad090

**Published:** 2023-04-17

**Authors:** Koichi Kitao, Hiyori Shoji, Takayuki Miyazawa, So Nakagawa

**Affiliations:** Laboratory of Virus-Host Coevolution, Institute for Frontier Life and Medical Sciences, Kyoto University, Sakyo-ku, Kyoto, Japan; Laboratory of Virus-Host Coevolution, Institute for Frontier Life and Medical Sciences, Kyoto University, Sakyo-ku, Kyoto, Japan; Laboratory of Virus-Host Coevolution, Institute for Frontier Life and Medical Sciences, Kyoto University, Sakyo-ku, Kyoto, Japan; Department of Molecular Life Science, Tokai University School of Medicine, Isehara, Kanagawa, Japan; Division of Genome Sciences, Institute of Medical Sciences, Tokai University, Isehara, Kanagawa, Japan; Division of Interdisciplinary Merging of Health Research, Micro/Nano Technology Center, Tokai University, Hiratsuka, Kanagawa, Japan

**Keywords:** genomics, transposable element, horizontal transfer

## Abstract

Independently acquired envelope (*env*) genes from endogenous retroviruses have contributed to the placental trophoblast cell–cell fusion in therian mammals. Egg-laying mammals (monotremes) are an important sister clade for understanding mammalian placental evolution, but the *env* genes in their genomes have yet to be investigated. Here, *env*-derived open reading frames (*env*-ORFs) encoding more than 400 amino acid lengths were searched in the genomes of two monotremes: platypus and echidna. Only two *env*-ORFs were present in the platypus genome, whereas 121 *env*-ORFs were found in the echidna genome. The echidna *env*-ORFs were phylogenetically classified into seven groups named env-Tac1 to -Tac7. Among them, the env-Tac1 group contained only a single gene, and its amino acid sequence showed high similarity to those of the RD114/simian type D retroviruses. Using the pseudotyped virus assay, we demonstrated that the Env-Tac1 protein utilizes echidna sodium-dependent neutral amino acid transporter type 1 and 2 (ASCT1 and ASCT2) as entry receptors. Moreover, the Env-Tac1 protein caused cell–cell fusion in human 293T cells depending on the expression of ASCT1 and ASCT2. These results illustrate that fusogenic *env* genes are not restricted to placental mammals, providing insights into the evolution of retroviral genes and the placenta.

## Introduction

Endogenous retroviruses (ERVs) are remnants of ancient retroviral infections to host germ cells. ERVs have provided de novo genes and new functions in their hosts (Johnson 2019). In therian mammals, various retroviral envelope (*env*) genes have been reported to be co-opted for placental fusogenic genes during evolution, the original of which is responsible for viral entry by fusing the retrovirus and the target cell membrane. *Syncytin-1* and *syncytin-2* derived from ERV-W and ERV-FRD1, respectively, are thought to be responsible for the cell–cell fusion of human placental trophoblasts (Mi et al. 2000; Blond et al. 2000; Blaise et al. 2003; Mallet et al. 2004). Placental genes derived from *env* are not restricted to humans, but also to rodents (Dupressoir et al. 2005, 2009, 2011; Redelsperger et al. 2014), rabbits (Heidmann et al. 2009), carnivorans (Cornelis et al. 2012), ruminants (Cornelis et al. 2013; Nakaya et al. 2013), tenrecs (Cornelis et al. 2014), and even in opossums belonging to marsupials (Cornelis et al. 2015). Moreover, non-mammalian species such as the viviparous *Mabuya* lizards were reported to acquire an *env*-derived gene, *syncytin-Mab1*, which was involved in placental cell–cell fusion (Cornelis et al. 2017). Given the widespread occurrence of ERVs in vertebrates (Hayward et al. 2015) and the ancient origin of retroviruses (Aiewsakun and Katzourakis 2017), the ERV invasion into the host genomes should have been occurring in egg-laying mammals (i.e., monotremes), which would be of particular interest to speculate the situation before the placental birth in mammals. Recently, the chromosome-level-assembled genomes of platypus and short-beaked echidna have been reported (Zhou et al. 2021). It enables us to investigate the monotreme ERVs in more detail. Indeed, in platypus and echidna genomes, we previously reported highly conserved genes that were derived from a retroviral reverse transcriptase, although their molecular functions are unclear (Kitao et al. 2022). Here, we comprehensively identified nearly full-length *env*-coding open reading frames (hereafter called *env*-ORFs) in the platypus and echidna genomes and found their dynamic evolution. Furthermore, a fusogenic Env protein identified in the echidna genome was experimentally verified. Our study demonstrates the ongoing acquisition of fusogenic *env* genes in mammals that do not utilize a placenta, providing key insights into the evolution of retroviral genes and the placenta.

## Results

### Discovery of *env*-ORFs in the Platypus and Echidna Genomes

**Fig. 1.**
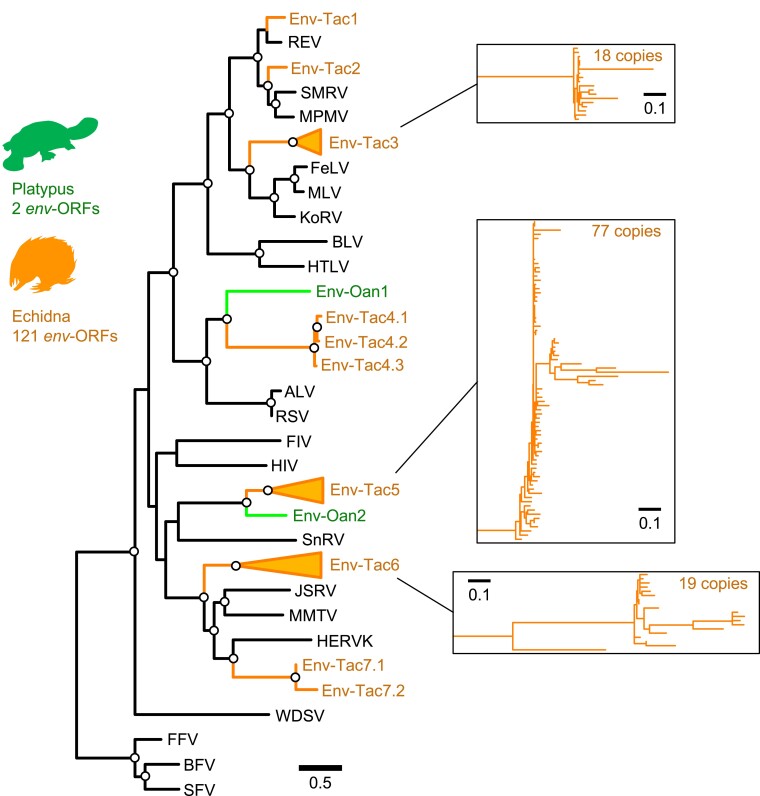
Phylogenetic tree of *env*-ORFs in the platypus and echidna genomes. Alignment of TM regions of the Env proteins coded by *env*-ORFs and representative retroviral Env proteins is used. An open circle in an internal node indicates >95% ultrafast bootstrap support (1,000 replicates).

**Fig. 2.**
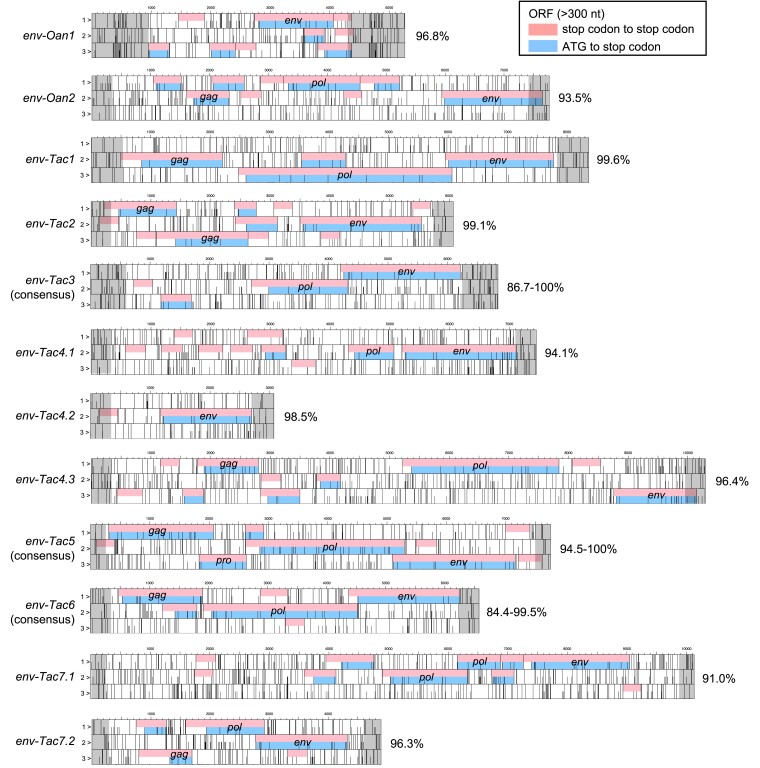
Proviruses encoding platypus and echidna *env*-ORFs. For each *env*-ORF, its retroviral structure, including *gag*, *pro*, and *pol* genes, was illustrated. Short bars indicate start codons, and tall bars indicate stop codons. ORFs of more than 300 nt were filled. ORFs from a stop codon to a stop codon are shown at the top, and ORFs from an ATG triplet to a stop codon are shown at the bottom. Retroviral gene names (*gag*, *pro*, *pol*, and *env*) were also indicated. 5′- and 3′-LTRs were filled with gray. Percent nucleotide identity between 5´- and 3´-LTRs was indicated on the right side of the sequences. For multicopy *env*-ORFs (i.e., *env-Tac3*, *-Tac5*, and *-Tac6*), consensus sequences were constructed.

**Fig. 3.**
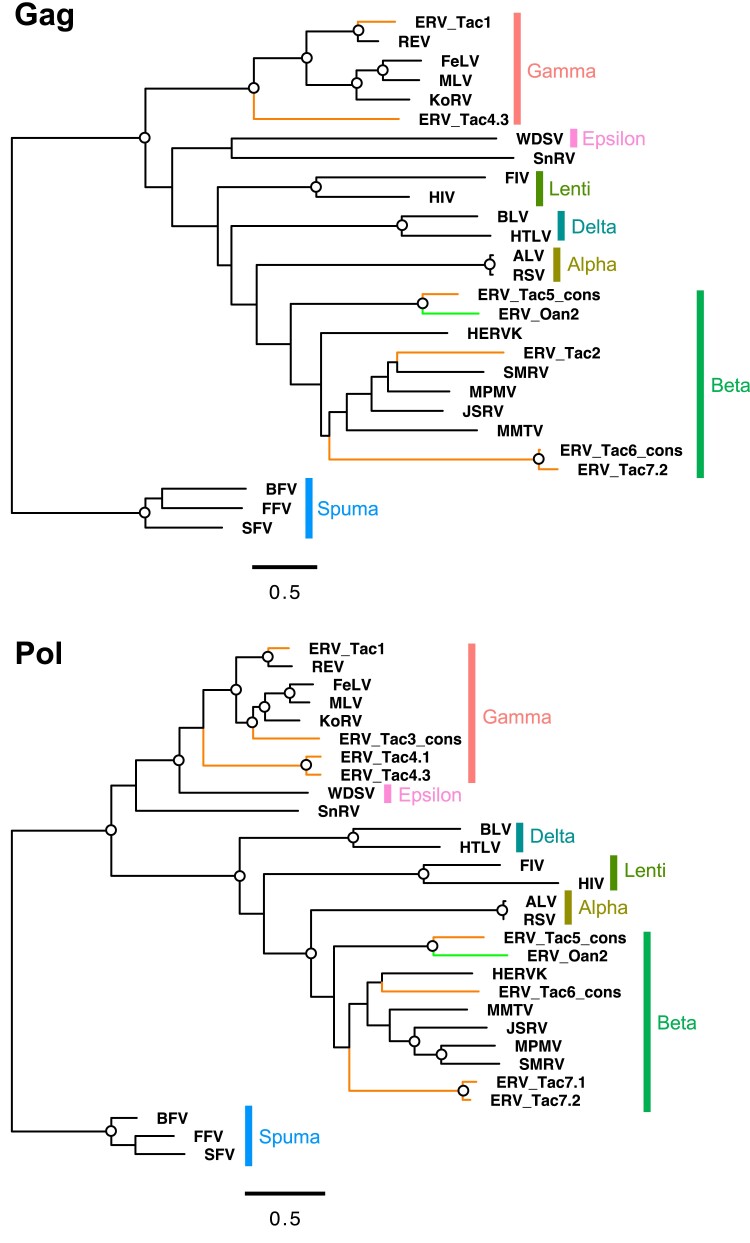
Phylogenetic trees of Gag and Pol proteins encoded in proviruses. The alignment of Gag and Pol proteins encoded in proviruses (fig. 2) and representative retroviruses was used for tree construction. “ERV_Tac” indicates a provirus containing the equivalent number of *env-Tac* gene (*e.g*., ERV_Tac1 is a provirus containing *env-Tac1*). An open circle in an internal node indicates >95% bootstrap support (1,000 replicates).

To obtain ORFs encoding nearly full-length envelope proteins in monotremes, we first extracted ORFs encoding more than 400 amino acids (i.e., 1,200 nucleotides starting from the start codon) from the chromosome-level-assembled reference genomes of platypus and echidna (Zhou et al. 2021). We then extracted ORFs encoding envelope proteins by sequence similarity search (see Materials and Methods). This search identified only two *env*-ORFs in the platypus genome. This observation is consistent with previous studies showing very few ERV-derived ORFs in the platypus (Nakagawa and Takahashi 2016; Ueda et al. 2020). In contrast, we identified 121 *env*-ORFs in the echidna genome. These monotreme *env*-ORFs were clustered into nine groups based on the putative amino acid sequences. We named these *env*-ORF groups as env-Oan1 and -Oan2 (platypus: *Ornithorhynchus anatinus*) and env-Tac1 to -Tac7 (echidna: *Tachyglossus aculeatus*) (fig. 1). Detail information of each *env*-ORF such as ORF length, chromosome, locus, direction, and overlapping gene or neighboring genes were summarized in supplementary data set S1, Supplementary Material online. The env-Oan1 and -Oan2 are found to have only one copy for each in the platypus genome. For echidna, the env-Tac1, -Tac2, -Tac4, and -Tac7 were also low copies (one copy for env-Tac1 and -Tac2, two copies for env-Tac7, and three copies for env-Tac4), whereas env-Tac3, -Tac5, and -Tac7 had 18, 77, and 19 copies, respectively. These high-copy-number *env*-ORFs were shallowly branched in the phylogenetic tree (fig. 1), suggesting the recent expansion in the echidna genome. Proviral structures containing *env*-ORFs were identified based on their genomic sequences (fig. 2 and supplementary data set S2, Supplementary Material online). Note that, for the high-copy-number env-ORFs (i.e., env-Tac3, -Tac5, and -Tac6), we generated consensus sequences of *env*-ORFs with flanking sequences and identified their proviral structures. The topologies of the phylogenetic trees of putative Gag and Pol proteins were consistent with those of the Env proteins and also suggested that these ERVs belong to either gammaretroviruses (env-Tac1, -Tac2, -Tac3, and -Tac4) or betaretroviruses (env-Tac5, -Tac6, and -Tac7) (fig. 3). Nucleotide sequences of 5′-LTR and 3′-LTR were almost identical in the high-copy proviruses encoding *env-Tac3* (86.7% to 100%), *env-Tac5* (94.5% to 100%), and *env-Tac6* (84.4% to 99.5%) (supplementary data set S3, Supplementary Material online), suggesting that they still actively expand in echidna genomes.

### Tissue Expression of Transcripts Encoding *env*-ORFs

**Fig. 4.**
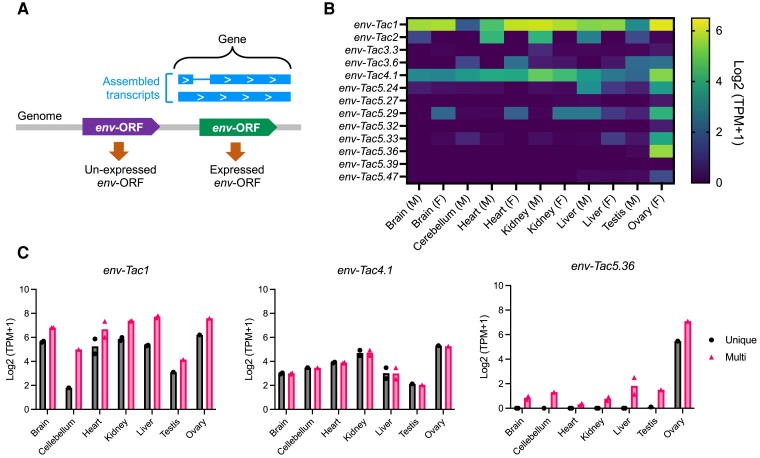
Tissue expression of echidna *env*-ORFs. (*A*) Tissue expression was calculated based on the genes of the assembled transcripts that overlapped with *env*-ORFs. (*B*) Heatmap rows indicate the expression levels of *env*-ORFs, and columns indicate echidna tissues. M, male; F, female. TPMs were calculated using uniquely mapped reads. (*C*) TPMs with or without multimapped reads. Dots show individual values, and bars show the means.

To investigate the transcription of *env*-ORFs, we calculated the expression levels of genes consisting of transcripts encoding *env*-ORFs from the platypus and echidna tissue transcriptome data (fig. 4*A*). In the platypus tissues, we could not detect the transcripts of *env-Oan1* and *-Oan2*. In contrast, *env-Tac1*, *-Tac2*, *-Tac4.1*, and eight *env-Tac5* loci were expressed in echidna tissues (fig. 4*B*). The *env-Tac1* showed the highest expression among these *env*-ORFs, whereas the *env-Tac2* was expressed only in male samples. When multimapped reads were included in the analysis, the expression levels of *env-Tac1* were increased, suggesting the presence of *env-Tac1*-like sequences in the echidna genome (fig. 4*C*). In contrast, the expression level of *env-Tac4.1* did not change when uniquely mapped reads were used. All expressed sequences in *env-Tac5* genes showed specificity to the ovary (fig. 4*B*). The expression levels of the *env-Tac5* genes, such as *env-Tac5.29*, were greatly increased when multimapped reads were used, suggesting that their sequences were similar (fig. 4*C*). These transcriptome analyses revealed that *env*-ORFs are actively transcribed in echidna tissues and have different tissue specificities among *env*-ORF groups.

### *env-Tac1* Potentially Encodes a Functional Env Protein

**Fig. 5.**
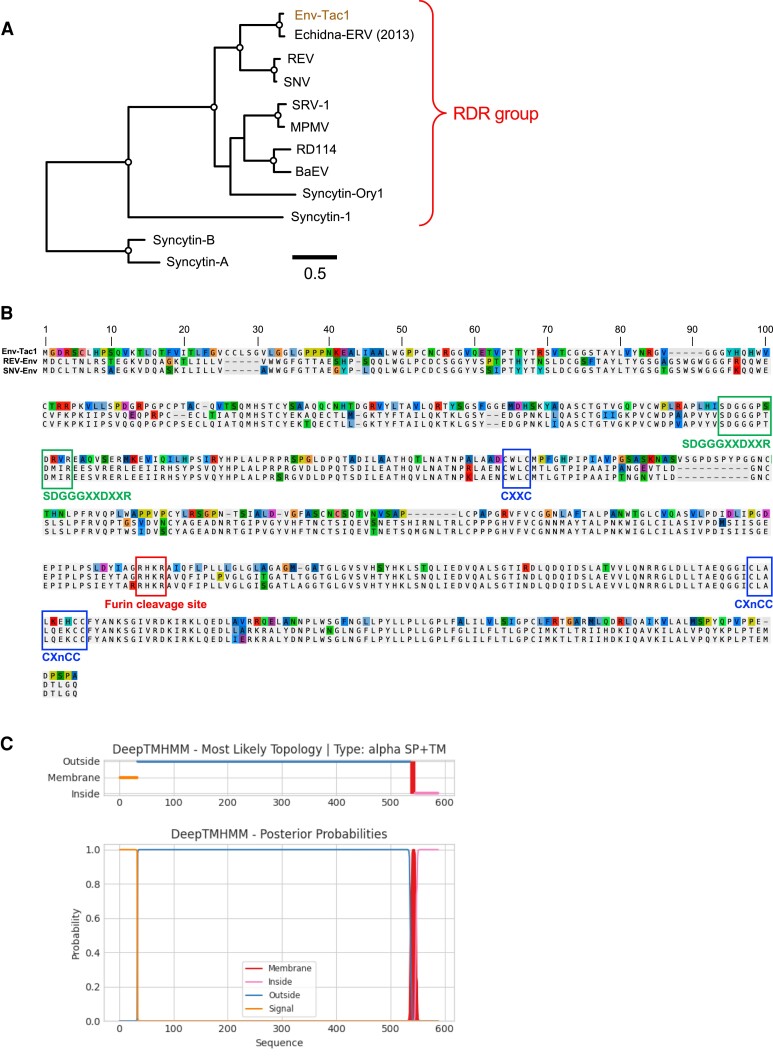
Characterization of the Env-Tac1 protein. (*A*) A phylogenetic tree of RDR Env proteins with mouse Syncytin-A and B as an outgroup. An open circle in an internal node indicates >95% bootstrap support (1,000 replicates). (*B*) Multiple alignment of amino acid sequences of the Env-Tac1, REV-Env, and SNV-Env. Functionally important motifs were indicated with captions. (*C*) Predicted topology of the Env-Tac1 protein using DeepTMHMM v1.0.13 (Hallgren et al. 2022). The Env-Tac1 proteins have the *N*-terminus signal peptide and a TM domain like other retroviral Env proteins.

The Env-Tac1 protein is closely related to reticuloendotheliosis virus (REV), which belongs to the RD114/D-type retrovirus (RDR) interference group (figs. 1 and 5*A*). Previously, Niewiadomska and Gifford reported a REV-like *env*-ORF lacking the SU domain in the echidna genome, as detected by PCR (Niewiadomska and Gifford 2013). The *env-Tac1* was thought to be a full-length copy of this ERV group, which was suggested by our phylogenetic analysis of REV-Env proteins (fig. 5*A*). A multiple sequence alignment of the Env-Tac1 protein with the Env proteins of REV and spleen necrosis virus (SNV), which is a subgroup of REV, showed that the Env-Tac1 protein retains functionally important amino acids (fig. 5*B*). In particular, the SDGGGXXDXXR motif is important for the binding to receptors, amino acid transporter type 1 (ASCT1 [also known as SLC1A4]) and/or amino acid transporter type 2 (ASCT2 [SLC1A5]) (Sinha and Johnson 2017). The predicted topology of the Env-Tac1 protein suggested a typical Env protein structure with a signal peptide at the *N*-terminus and a single transmembrane (TM) domain at the *C*-terminus (fig. 5*C*). Together, the Env-Tac1 protein retained amino acid motifs for functional RDR Env protein.

### Env-Tac1 Protein Interacts with ASCT1 and ASCT2 to Induce Membrane Fusion

**Fig. 6.**
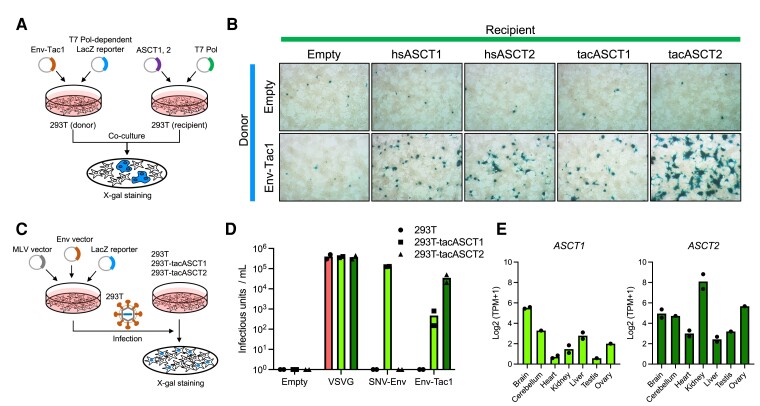
Fusogenic activity of the Env-Tac1 protein. (*A*) The cell–cell-fusion-dependent-LacZ assay. In the donor 293T cells, Env-Tac1-encoding plasmid or empty plasmid was transfected with the reporter plasmid, which expresses lacZ in the presence of T7 polymerase. In the recipient 293T cells, the plasmid encoding ASCT1 or ASCT2 of human or echidna was transfected with an expression plasmid for T7 polymerase. After coculture of donor and recipient cells, fused cells were visualized by X-gal staining. (*B*) The Env-Tac1 protein caused cell–cell fusion in the presence of ASCT1 and ASCT2 of human (hsASCT1 and hsASCT2) and echidna (tacASCT1 and tacASCT2). The LacZ-positive fused cells were stained by X-gal 48 h after transfection. (*C*) Human 293T cells expressing echidna ASCT1 and ASCT2 (293*T*-tacASCT1 and 293*T*-tacASCT2) were generated using the piggyBac system. The expression plasmid of VSVG, SNV-Env, Env-Tac1, or empty plasmid was transfected to 293T cells with the MLV virion and LacZ reporter plasmids. Supernatants containing pseudotyped viruses were infected with 293T, 293*T*-tacASCT1, and 293*T*-tacASCT2 cells. (*D*) LacZ-positive focuses were counted and viral titers per milli-liter were calculated. (*E*) Tissue expression of echidna ASCT1 and ASCT2 from RNA-seq data.

The fusogenic activity of the Env-Tac1 protein was experimentally characterized under the assumption that the Env-Tac1 uses ASCT1 and/or ASCT2 as receptors. First, we conducted a cell–cell-fusion-dependent Lac Z assay to verify the cell–cell fusion activity of the Env-Tac1 protein in the presence of ASCT1 or ASCT2 (fig. 6*A*). The Env-Tac1 protein was revealed to mediate cell–cell fusion in 293T cells in the presence of both human and echidna ASCT1 and ASCT2 (fig. 6*B*). The fusion activity was the highest on echidna ASCT2. These experimental data support that the Env-Tac1 protein is the RDR Env protein that utilizes both ASCT1 and ASCT2 as receptors. To investigate whether the Env-Tac1 protein enables retroviral entry, we generated murine leukemia virus (MLV)-based pseudotypes with the Env-Tac1 protein carrying the LacZ marker (fig. 6*C*). 293T cells stably expressing echidna ASCT1 and ASCT2 (293T-tacASCT1 and 293T-tacASCT2, respectively) were generated using a piggyBac system. We found that the Env-Tac1 protein mediated the infection of both 293T-tacASCT1 and 293T-tacASCT2 cells (fig. 6*D*). The infectious units were higher in 293T-tacASCT2 cells than those in 293T-tacASCT1 cells, which is consistent with the higher cell–cell fusion activity on tacASCT2. Env proteins of SNV mediated infection only in 293T-tacASCT1 cells, suggesting that there are differences in amino acids that determine whether they are directed toward ASCT1 or ASCT2. We examined the expression of *ASCT1* and *ASCT2* in various echidna tissues and found that *ASCT2* was particularly highly transcribed in the kidney (fig. 6*E*), in which *env-Tac1* was also expressed (fig. 4*B*). These data indicate that the Env-Tac1 protein is a potential fusogen in echidna tissues utilizing ASCT1 and ASCT2 as receptors.

### Estimation of *env-tac1* Insertion

We conducted a rough estimation of the times of the ERV insertion based on the 5′- and 3′-LTR similarities. The genome substitution rate of monotremes is estimated at ∼2.6 × 10^−3^ substitutions per site per million years (Zhou et al. 2021). It is known that 5′- and 3′-LTRs of an ERV are identical when inserted. Therefore, assuming that 5′- and 3′-LTRs of ERV-Tac1 are under neutral selection, the *p*-distance and inserted age of the two LTR nucleotide sequences were calculated for all ERVs obtained in this study (supplementary data set S3, Supplementary Material online). As for *env-Tac1*, the *p*-distance of the 5′- and 3′-LTRs is 3.9 × 10^−3^ substitutions per site, suggesting the ERV of *env-Tac1* can be inserted around 1.5 million years ago (MYA). Considering the two monotremes diverged around 55 MYA (Zhou et al. 2021), the *env-Tac1* could insert specifically in the echidna lineage. Indeed, the estimated time of all ERVs found in this study exhibits <55 MYA, excluding Env-Tac6.14 (estimated 60.1 MYA) (supplementary data set S3, Supplementary Material online). This result suggests that those ERVs were inserted after the speciation of the platypus and echidna lineages, which is concordant with the observation of the phylogenetic tree (fig. 1).

To further investigate whether the homologous genes of *env-Tac1* exist in the platypus genome as well as marsupial or eutherian genomes, we extensively searched using the FASTA program (E-value ≤ 1E^−10^) with the nucleotide sequence of *env-Tac1* against genomes of platypus, 24 marsupial species, and 5 representative eutherian species (summarized in the supplementary data set S4, Supplementary Material online). As a result, similar sequences were found, excluding platypus and the Asian elephant (*Elephas maximus indicus*) genomes (summarized in the supplementary data set S4, Supplementary Material online). To investigate evolutionary relationships between the *env-Tac1* and the *env-Tac1*-like sequences, we obtained the most similar sequence (*i.e*., a sequence showing the lowest E-value) for each species and generated a maximum-likelihood-based tree adding RDR *env* genes used in figure 5*A*. As a result, the *env-Tac1*, including echidna ERV (Niewiadomska and Gifford 2013), made a clade with envelope genes of REV and SNV (supplementary fig. S1, Supplementary Material online), suggesting that there are no homologous sequences in the mammalian genomes examined in this study. This observation is consistent with the estimated insertion time of ERV-Tac1.

## Discussion

The acquisition of the ERV-derived genes has been associated with the evolution of placentation in therians (Imakawa et al. 2015, 2022). The capture of *syncytin* genes from retroviral *env* genes are thought to be a pivotal step for the emergence of placenta in egg-laying ancestors (Lavialle et al. 2013). Indeed, the very small numbers of ERVs in the platypus genomes arose the hypothesis that intensive invasion of therian genomes may facilitate the co-option of ERV-derived placental genes (Chuong 2013). However, our study revealed that the echidna genome contained more than 100 nearly full-length *env*-ORFs. The number of echidna *env*-ORFs was not unusual compared with that in other mammals. For example, 37 and 255 methionine-starting *env*-ORFs longer than 400 codons were found in the human and mouse genomes, respectively (Nakagawa and Takahashi 2016), and 162 nearly full-length *env*-ORFs have been identified in 9-banded armadillo (Malicorne et al. 2016). Thus, the copy number of *env*-ORFs varies greatly among mammalian species, including the monotremes, during evolution. This observation also indicates that horizontal gene transfer via retroviruses could be common in mammals considering the evolutionary timescale.

Among echidna *env* genes, we demonstrated the cell–cell fusion activity of the Env-Tac1 protein (fig. 6*B* and *D*). *Env-Tac1* and its receptor *ASCT2* were found to be highly transcribed in the kidney (figs. 4*B* and 6*E*) suggesting that the fusogenic activity may be important function for kidney of echidnas. However, the biological process in which the fusogenic activity involved is still unclear. In addition, we have not examined whether the *env-Tac1* mRNA is translated. We previously revealed that *env*-ORFs, including *syncytin-1* and *syncytin-2*, require specific RNA motifs named SPRE to be translated (Kitao et al. 2021); however, the *env-Tac1* does not contain the SPRE motif, and its translational regulatory mechanism is currently unknown. Further studies are required to characterize the molecular function of *env-Tac1*.

Identifying the evolutionary conservation of retroviral genes is necessary for determining their co-options on host functions. Previously, we reported retroviral reverse transcriptase-derived genes *RTOM1*, *RTOM2*, and *RTOM3*, which are conserved in platypus and echidna (Kitao et al. 2022). The dN/dS analysis revealed that these genes were under purifying selection between the two species. In this study, however, no orthologous *env* genes were found in platypus and echidna, indicating that *env* genes are not conserved in monotremes. Given that most of the ERVs encoding *env*-ORFs still retained retroviral ORFs other than *env* genes (fig. 2), these *env*-ORFs could be under the process of mutational decay. Future comparative analyses of the evolutionary conservation of individual *env* genes in other echidna species, whose genomes have not yet been sequenced, will reveal the evolutionary conservation of individual *env* genes. In addition, we cannot distinguish whether each ERV cluster identified in this study expanded due to independent invasions or retrotranspositions, which required further studies. In conclusion, we demonstrated the diversity of *env* genes in monotremes and identified an *env*-derived gene in echidna showing cell fusion ability, which indicates the dynamic evolution of *env*-derived genes in mammals.

## Materials and Methods

### Detection of *env*-ORFs

The platypus genome (mOrnAna1.p.v1, GCF_004115215.1) and echidna genome (mTacAcu1.pri, GCF_015852505.1) were used for the *env*-ORF screening. The ORFs of more than 400 codons from start to stop codons were retrieved using the getorf program in the European Molecular Biology Open Software Suite v6.5.7.0 (Rice et al. 2000). To obtain ORFs similar to *env* genes, hmmscan in HMMER3 v3.2.2 (Eddy 2011) was used (expected threshold: 1E-10). The profile-HMMs of retroviral Env proteins retrieved from the GyDB collection (https://gydb.org/index.php? title = Collection_HMM) were used as queries (Llorens et al. 2011).

### Phylogenetic Analysis

To infer the phylogenetic relationship of monotreme *env*-ORFs obtained in this study, amino acid sequences of 20 retroviral Env were retrieved from representative retroviruses (accession numbers are listed in supplementary data set S5, Supplementary Material online). In total, 153 amino acid sequences were aligned using the MAFFT v7.487 with the L-INS-i method (Katoh and Standley 2013). Conserved TM domains of the Env proteins (regions after furin cleavage RXR/KR motif) were extracted. For the Env-Tac1 phylogeny belonging to the RDR interference group, we generated a multiple sequence alignment of full-length amino acid sequences of the Env-Tac1 and RDR Env proteins (supplementary data set S5, Supplementary Material online) using the same procedure. IQ-TREE2 v2.0.8 (Minh et al. 2020) was used to construct the phylogenetic tree with 1,000 replicates generated by an ultrafast bootstrap approximation (Hoang et al. 2018). The tree was visualized using FigTree v1.4.4 (https://github.com/rambaut/figtree/releases).

### Characterization of Proviruses of *env*-ORFs

The nucleotide sequences of *env*-ORFs with 10-kbp downstream and upstream extensions were retrieved using BEDtools v2.30.0 (Quinlan and Hall 2010). 5′- and 3′-LTRs were identified using LTRharvest (Ellinghaus et al. 2008) and LTRdigest (Steinbiss et al. 2009) included in GenomeTools v1.6.2. To obtain consensus sequences for the multicopy *env* genes (*env-Tac3*, *env-Tac5*, and *env-Tac6*), the proviral sequences were aligned with MAFFT as previously described. Then minor insertions were removed using T-coffee v11.0.8 (Notredame et al. 2000) with the “rm_gap 60” option. A consensus sequence for each *env* group was generated using the cons program in the European Molecular Biology Open Software Suite v6.5.7.0 (32). The ORFs in proviruses were visualized using ApE v3.0.6 (Davis and Jorgensen 2022). Characterization of proviral ORFs was based on the protein motif search in the HMMER web server (Finn et al. 2011). For the estimation of the integration time, 5′- and 3′-LTRs for each ERVs were aligned using MAFFT as previously described, and the *p*-distance was calculated using the distmat program in the EMBOSS with default parameters.

### Analysis of Transcriptome Data

RNA-seq data of platypus (20 samples from 6 tissues) (Marin et al. 2017) and echidna (11 samples from 7 tissues) (Zhou et al. 2021) were obtained from the SRA database whose accession IDs were summarized in supplementary data set S6, Supplementary Material online. Low-quality reads were trimmed and filtered using fastp v0.19.5 with default options (Chen et al. 2018). The filtered reads were mapped to each reference genome using HISAT2 v2.1.0 (Pertea et al. 2016). For each RNA-seq dataset, we conducted genome-guided transcript assemblies using StringTie2 v2.1.6 with “−rf” options to specify the strandedness (Kovaka et al. 2019). The resultant GTF files and the RefSeq GTF file were merged using StringTie2 with the “−merge” option to generate a merged GTF file. To quantify the expression level of each *env*-ORF, we calculated transcripts per kilobase million (TPM) for 20 platypus and 11 echidna RNA-seq samples using the Stringtie2 program v2.1.6 with “-e −rf” options (Kovaka et al. 2019). Exons in the merged GTF file which overlapped with *env*-ORFs were identified using BEDtools intersect with “-s” option to specify the strandedness (Quinlan and Hall 2010), and TPM of genes whose exons were overlapped with *env*-ORF were regarded as expression levels of *env*-ORFs. For extraction of unique-mapped reads, we collected reads that had “NH:i:1” flag, which indicates they were uniquely mapped, in aligner-generated SAM files. A heatmap of TPMs was generated using GraphPad Prism v9.4.1. Source data are included in supplementary data set S7, Supplementary Material online.

### Similar Sequence Search

We conducted a comprehensive search of *env-Tac1*-like sequences using FASTA program (*E*-value ≤ 1E^−10^) version 36.3.8e with the nucleotide sequence of *env-Tac1* against genomes of platypus, 24 marsupial species, and 5 representative eutherian species (summarized in the supplementary data set S4, Supplementary Material online). Note that the five representative eutherian species were chosen from five distinct *Eutheria* lineages: *Afrotheria* (*Elephas maximus indicus*), *Euarchonta* (*Homo sapiens*), *Laurasiatheria* (*Bos taurus*), *Glires* (*Mus musculus*), and *Xenarthra* (*Tolypeutes matacus*). For each species, the lowest *E*-value hit was chosen as the representative *env-Tac1*-like sequence. A multiple alignment of nucleotide sequences was generated using MAFFT as previously described. Gapped regions were removed using trimAl version 1.4.rev22 with a gappyout option (Capella-Gutiérrez et al. 2009). A maximum-likelihood-based phylogenetic tree was constructed using IQ-TREE2 as previously described.

### Cell Culture

293T cells (#RCB2202, Riken BioResource Research Center, Tsukuba, Japan) were cultured in Dulbecco's modified Eagle's medium (#D5796, Merck, Darmstadt, Germany) supplemented with 10% heat-inactivated fetal calf serum (#10270106, Thermo Fisher Scientific, Waltham, MA, USA) and penicillin (100 units/mL) and streptomycin (100 mg/mL) (#09367-34, Nacalai Tesque, Kyoto, Japan) at 37 °C in a humidified atmosphere of 5% CO_2_ in the air.

### Plasmids

To construct the Env-Tac1 expression plasmid (phCMV3-Env-Tac1), dsDNA encoding the *env-Tac1* ORF was synthesized by Eurofins Genomics (Tokyo, Japan). The synthesized dsDNA was inserted into EcoRI and BamHI sites of phCMV3 (#P003300, Genlantis, San Diego, CA, USA) using NEBuilder HiFi DNA Assembly Master Mix (#M5520AA, New England Biolabs, Ipswich, MA, USA). For the construction of the SNV-Env expression plasmid (phCMV3-SNV-Env), the SNV-Env-coding region was amplified from pPR102 (Dougherty et al. 1989) using PCR and inserted into the EcoRI and BamHI sites of phCMV3. To construct the human ASCT1 and ASCT2 expression plasmids (phCMV3-hsASCT1 and phCMV3-hsASCT2), human ASCT1 and ASCT2 were amplified from 293T cDNA and inserted into the EcoRI and BamHI sites of phCMV3. For the construction of the echidna ASCT1 and ASCT2 expression plasmids (phCMV3-tacASCT1 and phCMV3-tacASCT2), dsDNA fragments coding the ASCT1 and ASCT2 were synthesized based on the RefSeq sequences of XM_038750847.1 (ASCT1) and XM_038766835.1 (ASCT2) (Eurofins Genomics K.K., Tokyo, Japan) and inserted into the EcoRI and BamHI sites of phCMV3. For the construction of the echidna ASCT1 and ASCT2 expression piggyBac plasmids (pPB-tacASCT1 and pPB-tacASCT2), the GFP coding region of the piggyBac plasmid (#VB900088-2265rnj, VectorBuilder, Chicago, IL, USA) was removed by inverse PCR and was replaced with the echidna ASCT1 and ASCT2 fragments using NEBuilder HiFi DNA Assembly Master Mix. All PCRs described above were carried out using KOD One Master Mix (#KMM-101, TOYOBO, Osaka, Japan) with a C1000 Touch thermal cycler (Bio-Rad, Hercules, CA, USA). Primer sequences are listed in supplementary data set S8, Supplementary Material online.

### Construction of Echidna ASCT1 and ASCT2 Expressing 293T Cells

293T cells were seeded in 24-well plates at 2.5 × 105 cells per well. The next day, 0.4 *µ*g of pPB-tacASCT1 or pPB-tacASCT2 and 0.1 *µ*g of the hyper PBase expression plasmid (#VB900088-2874gzt, VectorBuilder) were transfected into 293T cells. Forty-eight hours after transfection, 293T cells were selected with 1 *µ*g/mL of puromycin. After passaging for more than 7 days, the selected cells were termed 293T-tacASCT1 and 293T-tacASCT2.

### Pseudotyping Assay

Pseudotyped viruses were produced by transfection of 2 × 106 293T cells in a 35-mm dish with 0.7 *µ*g of the plasmid encoding MLV Gag and Pol proteins (pGag-Pol-IRES-bsr) (Morita et al. 2000), 0.7 *µ*g of retroviral plasmid for nuclear localization signal-attached LacZ (pMX-nlsLacZ), and 0.7 *µ*g of Env expression plasmid (phCMV3, pCAG-VSVG, phCMV3-SNV-Env, or phCMV3-Env-Tac1) using 2.8 *µ*L of Avalanche Everyday Transfection Reagent (#EZT-EVDY-1, EZ Biosystems, College Park, MD, USA). Forty-eight hours after transfection, supernatants were filtered through 0.45-µm polyvinylidene difluoride (PVDF) membranes and transferred to target cells, which were seeded in 96-well plates the day before infection, with 8 *µ*g/mL Polybrene. X-gal staining was performed 2 days after infection.

### Cell–Cell-Fusion-Dependent-LacZ Assay

Donor 293T cells, which were seeded at 2.5 × 10^5^ cells per well in 24-well plates, were transfected with 0.5 *µ*g of phCMV3 or phCMV3-Env-Tac1 and 0.5 *µ*g of the LacZ reporter plasmid (pT7EMCV-LacZ), which expresses LacZ with internal ribosome entry site from encephalomyocarditis virus in the presence of T7 polymerase. The recipient 293T cells were transfected with 0.5 *µ*g of receptor expression plasmid (phCMV3, phCMV3-hsASCT1, phCMV3-hsASCT2, phCMV3-tacASCT1, or phCMV3-tacASCT2) and 0.5 *µ*g of expression plasmid for T7 polymerase (pCAG-T7Pol). For transfection, 0.8 *µ*L of Avalanche Everyday Transfection Reagent was used. Donor and recipient cells were cocultured 6 h after transfection, and the LacZ-positive fused cells were stained by 5-bromo-4-chloro-3-indolyl-*β*-d-galactopyranoside (X-gal) 42 h after coculture.

## Supplementary Material

msad090_Supplementary_Data

## Data Availability

All data generated during this study are included in supplementary information files, Supplementary Material online.
