# Supplementary material for: Dynamic Evolution of Retroviral Envelope Genes in Egg-Laying Mammalian Genomes
Source: Mol Biol Evol. 2023 Apr 17;40(5):msad090. doi: 10.1093/molbev/msad090 (PMC10152393; doi:10.1093/molbev/msad090)
Supplement: msad090_Supplementary_Data [file msad090_supplementary_data.zip › 230322_MBE_FigS1.docx]

**Supplementary Material for**

**Dynamic evolution of retroviral envelope genes in egg-laying mammalian genomes**

**Authors**

Koichi Kitao^1*^, Hiyori Shoji^1^, Takayuki Miyazawa^1^, So Nakagawa^2,3,4*^

**Affiliations**

^1^Laboratory of Virus-Host Coevolution, Institute for Frontier Life and Medical Sciences, Kyoto University, Sakyo-ku, Kyoto 606-8507, Japan

^2^Department of Molecular Life Science, Tokai University School of Medicine, Isehara, Kanagawa 259-1193, Japan

^3^Division of Genome Sciences, Institute of Medical Sciences, Tokai University, Isehara, Kanagawa 259-1193, Japan

^4^Division of Interdisciplinary Merging of Health Research, Micro/Nano Technology Center, Tokai University, Hiratsuka, Kanagawa 259-1292, Japan

*Corresponding authors: Koichi Kitao ([kitao.z7deb13@gmail.com](mailto:kitao.z7deb13@gmail.com)) and So Nakagawa ([so@tokai.ac.jp](mailto:so@tokai.ac.jp))

**Supplementary figure S1**

**Supplementary figure S1.** Phylogenetic tree of *env-Tac1*-like nucleotide sequences in mammalian genomes, related to **Figure 5A**. Nucleotide sequences of RDR Env genes used in the **Figure 5A** (summarized in **supplementary data set S4**) and *env-Tac1*-like sequences detected from 29 mammalian genomes (in *Italic* font, summarized in **supplementary data set S5**) were shown. A bootstrap value (%) is shown in the internal node if it is ≥50%.
