# Supplementary material for: Dynamic Evolution of Retroviral Envelope Genes in Egg-Laying Mammalian Genomes
Source: Mol Biol Evol. 2023 Apr 17;40(5):msad090. doi: 10.1093/molbev/msad090 (PMC10152393; doi:10.1093/molbev/msad090)
Supplement: msad090_Supplementary_Data [file msad090_supplementary_data.zip › 230322_Dataset_S2.docx]

**Supplementary data set S2. Annotated provirus sequences.**

LTRs were underlined and env-ORFs were colored in blue.

>ERV_Env-Oan1

TGTGGGAGTGTGCCAAGGTGGACCTGGGAACTGGCCACCGAAAGGTCAAAATGCCATCAGTGACGTAACCTCTCTGGCCAGAGGAGGCAGGACGGGAAGATAGGTGTCCCTCACCTCCGTGCCCACCCAATAAGGTATGGAAGGGACGGGAGCGGAAGGGGGCGGAGATTGAACAGGAAGGAAGTGGGGACCACCCAAACCAAGGTAATAAATACCTATTGCCTCTGGTGTTCGGGTCTCTCTCTGGGAAGATCACAGCAATAGCAACGGCCACCCACGTGTCTCTCCCTGTCGCGAGCACCAGACGCTATTTTGCCACCAGGAACAACGCACAGAGACAGGGGCCGCGGGACGGGTGAGTGTAACACATAACTGGGTTAAATACATAAGTGGGTAATGTATGAGTGTAACGCATATGTGAGCTTAGTGTATAAGCGAGCAATGTATAAGTGAGTATAATGCACATGTGAGTTTAGTGCGTAAGTGGGTAGTGTACAAGCGAGTGTAACGCGCAAGTGAGTTTAACGCACGAGTGTGTAACGCATAAGTGAATCTAAAGCCTAAATGGGGTCGGCGCATAAGTAGATTGAACGCTTAAGTGGATTCCTCCCAAGTGGGTGCGCACATGGTGGGAACTTGCCGCCTCACCACGTTGTTAGTAAACCATTAACGAGCTCTTCCTGGGCGGGCCCTGGTCTGCCCACACTCGAGAAACATACTAGTGTAATCCTCCCATCAGGGAAGCTTTAACACCAAATTCCTCCCAAAGGGGAAGGTTTAACTCCATACTTTCCCCAAAAAGGGAAGGCCGACTGTCGCGTCTAAATAATTAATTAATTCAAACCGCCCCGTGGAATAAATTCTATACAAAACTCAGGTTTTCCATCTCCAGCCTCTCTCTCTCTCTCTCCCGCGCCGATTCCGAAAGAACCTGTCCCCGGCAACGGGTGACATAAATGGCGACGAGGATGGGATCGGCGATAGGCGATAGAGAGCCGGGAGGGCTATTTGAATTCCTTGCGAATCAGGGATGGAAAACCCCGAGATGGGACAGCCGCTGGGTGGAGGACCATTGGGAAGATCCGGCCAATCTGGTGAGGGAGTTTTGTAATTGGCGTGAGGCTACCCAAATCAGGAAAAGGAAAGGAAAGACCACCCTTCTCGGCCTCCTAGCAACAGCGCTGCAGAGCGCTGCAGGCCGCCGCCCAGGATCGGCGGCGGTTGCGGCAGGAAAGGGACGCCCTGGAGGCGAAACTAAGGGAACGGGACACGAGATGCCTCTTATTGCAGACAGCGGCTGAGGTTAAAGCACAGCAGAACACCCTTTTGGAGCAGAAATTGGCACCCCGGGTGGCCAAGGAGTTAGAGGAGAGAAGAAACCTTCCGGTAACCGAGCAGGAGGTTAAGCAAGTCATGTACTCGGGAATTTGCCAGGGGAATTGCTCAGGGGAAGGGTAGGGAGATCTCGGCCCTTTACCAGAAGTGGAACCGCCCCCTCCCCAGCAGGATCGGGTGGTGCCCCCGCAGAATCCCGAGTGCCAACCCAAGCCCATCTACCCTATTATCAAGGAGGAGCGGAACCCTGAGGGAAAGGTCACCACCCAGACTATGGGCTTCACAGCCCCTGAGCTCAGGCAGATCTCTAAGGAGTTTTCCCGGGACCCCGGGGAACCGGTGATAACCTGGCTGGCTAGGATTTGGGAGGGAGCAGCCGCTCAGGTAGACCTCAGCCCAAATGAAAGCAGCCGCCTAGATTTGGGTCCAGGGGTGGATGTATCCCACACTCACAAGGTCGAACTCTTTGGACCCTCGCGGTACATTGGGCTCGGACGATACCCTGCAGTGAACAAGGAAGGGCCGGCACTCTCCGGTGGACCGACCCAACCACACTAATCCAGCATCTCCTGGTGCTTGGGATCGAACAAGTCCTGGATGATAGGGGGAAAACTCAGGAACCTAGTCCATGGTCCCTACCCGCCTAAGAGGGTTTACTTCATGCCATAGTAATGGGTGCACCCCCGGGGCCAGAGCATGCTCTGGCTCACCGCTTGAGGGATAGAGCCACAGGAGCAAGCACCTGGAGCACATTGGCCAATGTGGTCAATAGTGACTGGCCCCTATTTCAGTCCTCAGAGGTACCCATTACCCGGCGTGTAGCAACCATGATTGGGAAGGGCCTAGGTTTGAGGAGGACAAAGTCAGCCCCAACATATACTAGAAGGAACACCCGCCAACCTCCGAAGTGGATCGGCTCAGGAAGACTGGGCTTAGGACGGGTAGCTATTTGGCGGGAGTTGAGGGAACGGGGCTTTCCCATGGATGAGTTAGATGGACTACCCACCCATATCCTGAAACTGCTCACTCTGGAAGGTAGACCATGGGTCCCAACCATCCCGGAGGAGGAAGACTAGGAGGGAGATCCGGCAGGCCGGGCTAATGGACGGTGGCCGCGGCCCCCTTACAGGCCTATGACCGTCCTAATGGATGACCGCCAGGAAGTCTCTTTCCTGGTAGATACCGAGGCCCAGATCCTCCACCTGGGATGGGGCTCAGCACATCTTTACATAGCTCCTACAGGGATACCCTCCCCTGAGAAGGATACTGGAGACAGATTGGGAAGCGAGTCATTATCCGGCATTCCCCAAAAGGCCAGTTATGGGTTTACCCTTATGAGAGCTCTCATGGGACACATTTGGGCAGCTAATACCAATGAGGGCTGCCCACTAACCCTGGTAGAGGCTTGGACATCCTCCAAGACCTAAGCTCTTCTCTTTGGCAGATACCACACCTGGGAGAGGGGAAATGCCGACCCAGCGCTCGATGAATCTGCTCAGCCTGCTTGTCATTCTGACCCTGGAATGTGGGAGAGCCCGCGGCAACACCTTCTTGAAGGCCTTGGCCCACTACAGCCAGGACCTGAATGTTTCAAACTGTTGGGTTTGTGGGCATTCCCCATTGAATGGAGGGGGAGGGTACCCCCTTATAGCCCATCCTTATGACAACTCCACCTGGTTCAATCCAACGGGAGCCCCCCATCCCTGCAGCACCCGCCCTGTCACGGAGCCCGCAAATGCCACCACATACTTTATGGTGAGTGGGTGGACTGACCATGCCGTGTTCCCATTCTGCTTTCACAGTACTGGTACCAAATTCTGGGCAGGCAATAGTTCCCATGTATGTGACGTAGCTAAGCTCCAGGGCGGGCCCAGATCTACGGCCGGACATTGTGGCCCTCCGACCACCAAGCAGGGGGTGGATGTCCGCTCCCATGGCCTGCTCATTGCGAATGGGACCTTTGGCAACTGTGGCACCATCCCCTGTGCAGTACCTGCCTATCTAATTTGTGGCGTCCGTGCCTATTGGTGGCTCCCTGCCGGCTGGGGAGGGACATGCTTCCTTGGTTTTGTACTCCCAGCTATCCATCACACCCTGAGTCGCCCGGAAGATCAACTCCGAAACCGTCGGGGGGCCCCAATCTCAGAGTCTAAATGCTTCTTCGGGATTATGTTTCCTGCATATGGGTTTGCACGGGCGGCTCATGAAATACTTAACCTAGCTAAACTAATTGAACAGATAACCAATGACACTGCTGTCAGCCTTATGGCCCTCAGGGACGAACAGAAAGCCATCTGGACGACCGTCCTTCAGAACCGAATGGCCCTTGACTTCCTCCTCGCCAGCCAAGGAGGGGTCTGCAAGCTTATCGGGAAGGAGTGTTGCACCTTTATCCCTGACAATTCTGGACATGTGGACGCAATTGTTGCTGATATGTACCACGCAGTTAACCAGTACCGGAATGATGACACTGCTGGAGGAGTCTGGGACTGGTTCCAAGGACTCTTTACGAACTGGGGGAGCTCACTATTCCATGGGTTGCTGCTGCTTTCGCTCTTGTTGGTGGGCATAGTGGGGGGATGCTGGTTCCTAGGTTGCTGTTGCTCGGTCCTCTCTACATGCATGCGACGAACACTGAAGTCATCCCCGACTCCTCTCTCTGTGCATTACCTCAAGAATGTTGTAGTCCACCAGTCACCCTCCCTACCTCTGGTCACCACACCTTAAAGGCTCCGCCTCCCCCCCCCCGTCCTAAGTCCCTGGCCAGAGCACGGGAGCTGTCCTCTGCCCCGACCTATGGATCTACTCTCTGGACTTCCGTGGCCAGCCAGGAGAGGATATCAGTTCCTTGTGGTCAGGGGAGGGACAATGTTGGAGGGCACGGCCTCCGGCATCACTACACCACCACGCGCCCCGGAGGGGATGCAGGGAAGGTTGGGGGTCAAATGGGCACGAATGGCGAGGGCCAAGGGGTGGTATGTGCCAAGGTGGACCTGGGCACTGGCTACCCAAAGGTCAAAATGCCATCAGTGACGTAACCTCTCTGGCCAGAGGAGGCAGGATGGGAAGATAGGTGTCCCTCACCTCCGTGCCCACCCAGTAAGGTATGGAAGGGAAGGGAGCGGAAGGTGGCGGAGATTGAACAGGAAGGAAGTGGGGACCACCCAAACCAAGGTAATAAATACCTATTGCCTCTGGTGCTCGGGTCTCTCTCTGGGAAGATCACAGCAGTAGCAACGGCCACCCACGTGTCTCTCCCTGTCGCGAGCACCAGACGCCATTCTGCCACCAGGAACAACGCACAGAGACAGGGGCCGCGGGACGGGTGAGTGTAACACATAACTGGGTTAAATACATAAGTGAGTAATGTATGAGTGTAACGCATATGTGAGCTTCGTGTATAAGCGAGCAATGTATAAGTGAGTATAATGCACATGTGAGTTTAGTGCGTAAGTGGGTAGTGTACAAGCGAGTGTAACGCGCAAGTGAGTTTAACGCACGAGTGTGTAACGCATAAGTGAATCTAAAGCCTAAATGGGGTCGGCGCATAAATAGATTGAACGCTTAAGTGGATTCCTCCCAAGTGGGTGCGCACATGGTGGGAACTTGCCGCCTCACCACGTTGTTAGTAAACCATTAACGAGCTCTTCCTGGGCGGGCCCTGGTCCGCCCACACTCGAGTAACATACTAGTGTAAACCTCCCGTCAAAGAAGCTTTAACACCAAATTCCTTCCAAAGGGGAAGGTTTAACACCATACTTTCCCCCAAAAGAGAAGGCCGACTGTCGCATCTAAATAATTAATTAATTCAATCCGCCCCGTGGAATAAATTCTATACAAAACTCAGGTTTTCCATCTCCAGCCTCTCTCTCTCTCTCTCCCGCGCCGATTCCGAAAGAACCTGTCCCCGGCAACGGGTGACAGGGA

>ERV_Env-Oan2

ATGGCTGGCGTGGGGGGCCATGGGAGCACAGGGAATGGCAAGTGGCCGAGCCAAAAAGCGAGTACCTAGCAAATAGGTCCAGGCTGAGAAAAACAAGTCAGCCTTGGGAGGCCCCCAGGGGTATGCAGTCACGCACGGCATCCGAGGTCAGAGATGTTCCCACAGTATCCGAGGTCAGAGATGTTTTCATCTGGTCTCCCTCTGATAAGGAAGTCGGGGTGCAGGTGTATGCCAAATAAACTGGGACGTGTTCTCTTCGCTTATTGGTGAAAGCTGGTTGCCATGCCACATGACCCGCTTGGCGGATAAAAGGGAATGCGAGGGGAGAACGGGGGCTGCCCCCACCGCTTTCGGTGCAATGTAACGGGAACTAATAAAGCTGCTTCAAGTTTGGTGCCTCTGGCTGACTCTTCCTTGGGAGAACGCGCAGGCCGCGTCCAGAGACGTCCAAAGACCTGAGATGGTAAGACCCTAGCATTGCGGGAAGCGGAACGCAACGAGTGGCACCCAAAGTGGGGCCCGAGGGAGAGGGGACCCCATAAAACAGGAAGCCAAGGAGGAGTGCCCCGTAGACTAGGGAGCTCATAGTCAGGCGGGAAGATGGGAGTGGAAAAGTCGCTGCCCCTGTTTAAGCCAGATGAGATGGAACTGTATGTACCATATTGTTATAAGATTCTGAAGGAACACAGGATGAACGTACATCTGAAGATTAGGGAATTTATCGGGAAGCTGACGATGATCTGCCCGTGGATCTCTCAGTCTGGACTGACTGGGGATTAGTGGAGGATAGCGGGGGAACAGATGACAGCGTATGAGGATCCTTCCCCGGGAGAGCTTACCGACCATGATTTTCTGATTTATGGCATCCTAGGGGCTGCGCTCAACGGGCCCGAGTGAAAGCCATGACACTTTAATGTCACCAGCCAGACAGAGGGTGCAGGGGAGATAGTACAGCGCCCCCCCACCAGCGACCCCGACACCGAGCTATCACAAGCTATACCCCGATCTGTCATCCGTAGGCGCGCCCTCCAGTGTAGTCCTTGAGACGCGAGATACAGCAGAACAATCGGGGCGGCTAAAAGTGATAGTATGGCGCAGTCAGAAAAGGAGGCAGAAATGACACGGCTTCGAAGGCGGCTAGCTGAACTAAGTATGGGTAGCAGGCTTAAGCACGATTCCCGAGAAAGAGGGGTGGGAGCAGAGGCTTGCGCAGGTCCGCCCGGAACGGCGATGCAATTGGCGCTGGAGGAAGGCAGAAGATTGGGGGAGGACACAGGGAGTTGGGACGCATACCCCGTGATACAGGATGGGGACGGGCAATGGGCTTTCCAGCCGATAGCGTGGATTAAACTAAAGGAACTAAAATCGGCCTGCTCCTCCTACGGGCCGGACTCTCCCTATGTGCAGCAATTGCTCGAAAATCTGACCTTAGAATCAGTCCTCACGCCAAATGACTGGAAATCTCTGGCTCGAGGTTGTTTAGACCCGGGTCAATCTATAATCTGGATGTCAGAATAAACAGCTGTGGTGAAGGATATGGTAAAAACAGCACGAGTTTCATAACCCCGCCGAGGACTTCTCAATATTAGCAGGAATGAGTAGGAATTAGCAGTATGAGACCACCGAAGCTCAATTATTGTACGACCCCCCGACGTACGTACTAGTGGGACAATTAGCCTTGACAGCCTGCAGCAAGGTCCCCCAGAAAGGGGACCGGCGCTTGCCCATGACACAGATCATGCAAGGATCTGGAGAACCTATACATGACTTTGTCTCCAGAATGCAACAAGCAGTATTGAGGTCTATAGGCGACAATGCCGGAGCGGAGATAGTCCTGAAACAGATGGTCAGGGAAAACGCTAACCCTGCTTGTAAAAAGGCGATGATGGGCCTCCCAAAGGATGCGCCCTTGGAAGATATTCTTAGCCGGTGCGAGGGATTGGGGGGCGAGGAATATAAAGCACAGGTGCTAGTGGGGGCCATAGTGAAGGGGTTACGTGGAGGAATGTCTGAGCAGAGGAAGTGTTTCTGCTGTGGTAAACAGGGACACTTGAAGGCACAATGCCGTGTGGGAGGATCCCTGAGCGTTGGGGCCCAGGAAAGGGGCGCCCCAACCTGTTTTCAGTGCGGGAAGCCAGGGCACTATGCGAAACAGTGTCGGCAGAAAAGGCACAGACAACAGTCGGGAAACTGGCGGGGAGGCCCCGCACGGGCCCCGCACGCCTACCCAGTGACGCAGTCAGAACCAAAGGACTCGCTACTCCAGGAATATCGAGAGCTCCAGACACAGGAACCCCGTGGGCAGCTGTCCGCTGCATGGACAATGTTAAAATAAACCCGGGGGACACTGTGGCCATCCCCATTTGGCCATTGCCACGGGACGTGCTTGTCACCGGTCTCGAGTCCCGAACATCAGGGGTGGTTCACACCACCACAATGGGGGGAGGCTCAAGCCAAGTCCCCCTAACAAATCCTTACCCATACCCTATATTCATTGAAAGATCGAGACCCATAGCAAAGGCCACACCGCTAGAGGCGGGACCCCCGGACCATAACCTTAGCCAAACCTTGGAAGACGGTGATTGTTGAAGGGATTCCTTTTCGAGGGACTCTCGACACCGGCACTGACCGCTCGGTGGTCACCCACTGCGATTGGCCAGCCGCGTGGCCAACGACAGAGGGACAGATGGGCATAAAAGGGATAGGCGGAACACAAACCGCCCACGAGGCTGACAGATCCCTAGCCTGGAAATGCGAGGGAAAACAAGGACAATTTGTGCCCATTGTGGTCCAAGACCTGGGAACAAACCTGTGGGGGAGAGATGTTATGCAGGGTATGGGACTAAAATTGACTGATCAGGCTGAAAATTTTGGATAGGGGCCATTGGGGTGTGGCGCCTGGAGACACCCGCGATAGTCTGGAAATCTTCCGAGCCTGTGTGGGTGGACCAGTGGCCCCTCACCTTAGAAAAGCTTCAGGCGCTCACGGAGATAGTAGAGGAGCAATTGAAAGCAGGGCACTTGGAGATCTCGTTTAGCCCCAATAATTCTCCCGTGTTTGTGATAAAGAAGAAAAACACTGGGAAATACCGCATGCTTATGGACCTCCGAAAGATTAATGCCCTTATACAGCCTATGGGGCCGCTTCAGGTGGGTTTGCCCTCCCCAAATATGATCCCAAAGGGGCAGTCTATCAGGATCCTTGATATTCGTGATTGCTTTTACAGTATCCCACTGCACGAGCAGGATAGACCCTGATTTGCCTTTACGGTACTGTCCATTAATTTTGCTGCACCCACTAAGAGATACCAATGGAAGGTCCTGCCCCAGCGCATGGTAAACAGCCCGACACTTTGTCAATGGTACGTCGGGAAGATACTGGAAGATATCCGATCACAGTACCCCGACGCCACCATGTTACACTATATGGATGATATCCTTTTGAGTCACCCATCTCCGGGTCAGCTTCACTCCCTAATGGTCGCAGTGATCGAGCACCTCAAGCACTACAGCTTGGTGGTGGCCCCTGAGAAAGTCCAAGACAAGGAACCATTCTTGTATTTGGGATTCTCCCTCCTGGGTGATCGAGTAACACAACAGACACCTCAGATCGAATTCGGTCGATATACCACTTTGAATGACATACAGCGCTTGGTGGGGCAAATCCAATGGCTCAGAGCTAGATTGCCCATTCCTTCGGGCTTGATGGAGCCCTTGTATGATCTCTTAAAAGGGGACCCAAACCTCCGGTCACCCAGGGAATGGACGACCACCGCCAAGGATGCGGTCCAGACAATTCTCCAGGTAGCTACCCGGGGCTCCACTGACCGGGTGGATCCGACGGCATGGTTGGAGGTAACTGTCTTTAGAGACCATGAACTGTTTGCCGCCTTCCACCAAGACAATCGGGTGCTCGAGTGGAGCTACTCTGGTCGCACCTCTAAGGTGCTGGAGCGGGAGGAGGTGATTCTTGGACGTTTCTGCCTCACCCTAATACGGCGGGTTCGAGTGCTGATGGGGAGCACTCCGATTATCTACTTGGGCATATCCCAGGGGGAGGTTGATGCCCTAGCACAAGAAGATCTCACCTGGGCGACAATCACGCAGACGGCTCATGTCGGGGAGAGGTCGACCCTCTTGATGGGTCCCCTCCTCCGGAATACCTGGCTGTTGCGCGCACCTGTGGTTCAGACTGAACCAGTAGCGGGCGACAATATTTTCACGGACGCAACCAAAGATAGGAGAGCCGCGGTGTATAACCAGACCTCAGGTGCCTTGACCGTGCTCTCTACCCTGCATGAGTCCACGCAGAGGAACGAGCTTTATGCCATCAGCTGGGCGTTGGAAAAATATTCTCAGCCTATTATTTCTGACAGTTTATATGCAGTTAGCCTTGTCAACAGGTTGGAGACGTCGATCCTCTTTGACCGCCGCTCAGAAATCGGGGAACAGCTGTGTGACTTGCAACGCAGGCTGCTCTCCCGGACAAGTCCTATATATGTTATACACATCCGGTCTCATACCGACAACCGCGGGCCGGTGTTTGCGGGTAACTGAGTGGTGGACGCCAGCCTCTATCAGGTACTAGCGGGGGGATCCTTCCCGGAGTCAGCGCAGAAGGCGGACGCCCTTTTTCACCTCCCCGCTGTTTCCCTCCGCCGCCTGTACGGTTTGACGAAAGCGGAAGCCCGGGGCATCGTACGCCATTGCACCCGGTGCCTCCCGTTCCTTACACGCCCACCCGGCCAGAGCGGAGTGAATCCGCGCGATTTGGTCCCGAATGACATCTGGCAGATGGATGTCACCCATTGGGGGCGCGACTGCGTCCATGTCTCCGTGGACACTCACTCCGGATTCCTGATGGCTACGAAGCAGCCAGGTGAGGCGGTTCGGCACGTGCAGAGCCACCTGTACCACTGGTTTGCCAGTAGCGGAGTGCCAAGGGAGATCAAGACAGACAATGGTCCGGCGTACACGTCGCAGACCATGGCCAAGTTTTTCACGACCTTTGGCATTAAACATGTCACCTGCATTGCCTATAACCCTAATGGTCAAGCCATCGTGGAGCATGCCAACCGGATGCTCAGGACCCTCTTGACCAAACAAGGGGTGGGATGGCGGGTGGGCCAGAGGGAGCTGGACACGGCAGTCTATACCCATAAGTTTTCTGAGTGTGGACCGAGTCTCTGGCCTCACCCCGGCTATGTGGCATCTGTGCGTCGCGGATAAGTCGGGGTTGCCCTCGCGCTGGACGCAAATGGATGACGTGGCTCAACAGGCTAAGTGGAAGGACGCCCAAGGCGGGTGGCAGGGACCACATCAGGTTCTTATCCGTGGTCGAGGATATGCTTGACCAGTCATATTTGGTCAGAGGAGAGGGGCCAGTCTGGGTCTCAAGCCGAAACTTCCGGGTCCTCGAGGGGCGACTGACGATGCTGACGGCAGTGACCCGGCCTCGGTGACCTCGGCCCCCTCGGAGGGCGGTGCACTCGATGCTACAACTTCTGCTGCTGCTCTCGCTGGGCAGTGGCGTCGGAGCAGTGCCAGGCCCAGCAGGAGGCCACCAAGTCACCTGGCAGGCCTGGTTGACTCCGCGTTGCTGGCACAGGAGGAGGTGAGCGGAGAATGGTGATGGGTGCTGGTACCACACCCTCCGGTGGTGCGGCTAGTCTCATGGCGGGAGGACTCGCCAGACCAGATCCTGTGGGGTAATATGTCCAAACTGACGGGCCAACCACGGGACACTCGTCAGAACCGTGGGGAGGAGGCGGTTGCATTGTGGAACGTTACCCTCCGGAGCCTGGGTTTGCCTATTTGTTTTTATGATGCATCCCGGAATGTGTCTGCATCGATATGACGGTACTGCTTGGCCCTCCGCAGCTGGCCGGTCACGCAGTCACCCTTCTCCGGGGCGAGGGGCATCCCACTCACCGGAACCTCACCATGATGGGTTTACACGGGGGACTTCGGTGTACATGGCCACGCCTTAATGTCACTGTTACCGATCACTCCGAGGGGAAATTTGGGCCAATTCGTAACTCCTCCCTTATCCCTTTTCCACCCCCTAAGGCGCTGCGCTGAGAGCTGTGTGGGGGTGTGGGTTCCACTGACCCGATGCTCTACGAGCATCTTCCGATGTGTAGTCTTGGGGGACAATGTCACCACCCTTATTACTTGGGACAATCCCATTCGTAACCACTCCTATAGTGAGGCCGCCGCCCCGGTCTTGTTTGCATCTCTGGCCGGTACCCAACATCCCGGCCTCTGGAAAGCCTTCACTACCTTGAGGACCATGAACGTGACGGTTCATTCATGTCCGTGCAAGGGGCCCTGTCAGGATTACCCGTATCGGGTGTGGCGGGCGGTCTTCCGAGAGGGCCTTAACATCACCCTGGAATCCACGCCGGAGAACTGTACTGATTACCGGTACCATTTCACGGGCCGTATCTTGATCCCATCCCCTTATGTTTTTTTGCATTGTCAGCTTGGCTCGAACCGCTCGGTCGAGGTGAGCGCCACGTGCGCCTCTGACTCCGACTATGCTCTCTTCGCCTCAGATTGTGACCTCACGAATGATTCCCCCTGTCAGGGACATGGGCAAGGGATAGTCACATTTCTGGTCCGGCGGCCCGGATACCTCCTGGTTCCGGTCCGCTCGGCACACCCGTGGTTCTCTTCCCTGTCCGAGCGCGACTGGTATCACGTGTCGCAATGGATCAAGCAACAGAGGCGGGAATTGTTTGAGGCTGTTTTGGGTATTGCCAGCCTGGCTTTCTCTGCCTTTCAGGAGTGGCAGATCCAAAACTTGTTTGATGGGATAGGGTATCTCGGCTCGCAGCTTCAGGCTTTTATGCATAGCACTTGTCAGGCCTTCGTGGTACAGCAGCAGCTTGATGTCTCGCTTCAGCAAGAGGTAGTGGGCTTGGAACGCGTCCTCGAAATGCTTGGGGATGAAGTGCGCCTCCTGTCTTTGAGACAGGAAGTTCAATGCGATTACCGTTACCGGCATGTATGTGTTCTTTCCCTCGTTTGTAACGCGACTAACTCCTCTTTCCCAGGCGACTGGCTCTCCGTTAAGCGTCACTTGGAAGGCCTCTTCTTGGCGGCGAACGTTTCTTCCGAGCTACAGGAGTTGCGGCTTCTGGTCAACAAATTGCGCGCTCAGGAGCTGAACTTCACCTTGTCCGGCCCGGCCAAGGACTCTCACCAGCTGGCTGTCTCGACAGGCGCCGGGGGGCCTGCCCCCTTGGCTGTGGCATGTGGTGACTGCCGCTGGTTTGTTCCTGCTTTGTCTTCTATGCCTCCCGTGCTTCGTTTGCCTTATTCTTTGGTTCCTGCGCCATGCAGTCCGGGACGTGGAGACCGAATTGTTCGCTCTTCACATGCGACGGCCCTGTGCGGTCCCCGCATAAGGGAAAGGGGGAGACGTGGGGGGCCACGGAAGCACGGGGAATGGCAAGCGGCCGAGCCAAAAAACGAGTACCTAGCAAATAGGTCCAGGCTGAGAAAAGCAAATCAGCCTTGGGAGGCCCCCAGGGGTATGCAGTCACGCACGGCATCCGAGGTCAGAGATGTTCCTACGGTATCCGAGGTCAGAGATGTTTTCATCTGGTCTCCCTCTGATAAGGAAGTCGGGGCGCAGGTGTATGCCAAATAAACTGGGACGTGTTCTCCTCTCTTATTGGTGAACGCTGGTTGCCATGCCACATGACCCGCTTGGCTGATAAAAAGAGAATGCGAGGGGACAACGGGGGCCGCCCCCGCCGCTTTT

>ERV-Env-Tac1

TTGTGAGGCTTGGAGAGGCTTGAGTCTCTACAACTAGACAGACCCCTATCTTGGGCAACCGGGCCAGTAACACAAGGAAGGAACATTTGCATAACCGCTCCTCAGACCCCTATCTTGGGCAACCGGGCCAGTAACACAAGGAAGGAACATTTGCATAACCGCTCCTCAGACCCCTATCTTGGGCAACCAGGCCAGTAACACAAGGAAGGAACATTTGCATAACCGCTCCTCAGACCCCTATCTTGGGCAACCAGGCCAGTAACACAAGGAAGGAACATTTGCATAACCGCTCCTCAGACCCCTATAAAGGGTGTAAAGTTGTGCTCGTGGGTGCTGCCGCTTCCCAAGCCTTACTATAAGGCGGTAGCCCAGATTTAAATCTGCAATAAAGCTGCGACCTGCCCTCTTTGGCGCTGGGCAGGTCATCTAAATTTTGTCCGGTTGTCGTGCGATCCAATTTGTTGGTGGGTGGGATCCCGGGCCCTGGAGACCTTGATCAGGTTTCCTTACAACATTTGGGGGCTCGTCCGGGATCCCCACCATTTTAGGCGGACCCCTTCGCTCCGCCATTCGGGATCGGCGCAACAGACCCGGTACCGGTGAGTCACTTTGTCAGGCCTCGCGAGGGTTTGGGAGTATAGGAACGGCAGGACGCTGCCTTTGTTCCGACTCCACTCGGATCAGGGGACGCTCTGATCTCGAGTTTGGGTCTCATGGTCGAGTACAGTTGTCCGTGAGACGTGTATGTTTTGGGCTGTTTGTTTGGTTGTTGGTCCGTCGTTTTTGTTCTTTGTGCGTCTTGCGAATTGCCGGTGGACCCCGGCTAGAGCCGAGGGAGAGGGGGCGCATGCGTAATGTTTTAATCTTCGGGCGAGTACGGGGTACAGGAAAGGGCCCTTCAAGCCCGTTGGAACATCTTCTTCGGTATTTTTGTCTCCCAGTTTTTCTGCTCGCACTTGCCGTCCTCTCCCTCTCGGCCAAGATGGGACAGGGCAGGTCAAAGGGCCCCTTGAGCCCGTTAGGGTGTTTGCTCAAACACTTCTCTGATTTCCAGCGGCGAGCTGATAACTATGGCGTGTCTGTTAATAACTTTGACTTACGCAGGTTTTGCGAGTTGGAATGGCCCACCTTTAAGGTTGGCTGGCCTGACACTGGGACCCTAGACATAGGGGTGGCGGCCGCCGTCCGCCGAGTTGTGGACGGGAACCCAGGCCACCCGGACCAAATCCCATACATTACCATTTGGATAGATATTATAGTAGACAACCCTAAGTACTTAAAGGACTGCGGGTGCCGGCCCCTCAGCGCTTCTAAGGTCCTCGTATCCAGTACTCTAGGGTCCAAAGCTGCTCGCAAGCCTCCCGTGCTCCCCACGCAACCGGAGAGCCCGAGGAGGGGGCGAGCGCCTCCTCCGCGAGCGCCCCCTCCCCCCTATAGGGAACCCTCAGCCCCGCCCGAGGAAGAGGCTTTTCCCCAACCAGATTCCACCGGACCTCCAAGCCCCCCCCACACCCGAAGTGGGACTGAATTTGGGCCGACAGGAAGGGCACCGGGGGTCTCGGGAATATATCCCCTGAGGGAAACAGGAGAAAGGGATGAAACGGGGCGGCCTGTGCGGACATATGTTCCTTTTACCACGTCAGATCTGTACAATTGGAAGAACCAGAATCCTTCCTTTTCCCAAGCCCCGGAGGAGGTAATCAACTTACTAGAGTCAGTCTTCTATACCCATCAACCTACTTGGGACGACTGCCAGCAACTCCTCCGCGTCTTGTTTACGACGGAGGAAAGGGAAAGAGTAAAGGCAGAGAGCAAAAAGGAGGTCCGAAATATTCGCGGTGAACCAAGCACTGACGCAGGGGAAGTGGAGGCCCAGTTCCCCTCTGGCAGGCCTGATTGGGACCCCAACACCCCGGGAGGGGAGGCCAATCTGAATCAATACCGCCAGATCCTCTTACGGGGGCTACGGGCGGCGGCCAGAAAGCCGACTAATCTCTCTAAGATAACCGAGGTCCGGCAGGGGCCAACGGAAAGTCCTACGGCCTACCTGGAACGACTATATCAGGCCTACCGGACCTGGACCCCCATAGACCCTGGGAGTCCTGATAATCAGGCAGCTATAGTAATTCAATTCGTGTCGCAGTCGGCCCCAGATATCCGAAAAAAAGATTCAAAAAATGGATGGGTTTCAGGGAAAGCCTCTCTCTGAGCTGGTAGCCATAGCCCAGAAGGTTTTTGACCAACGAGAGGACCCCACCAGAACAACTTATGAATTAACCCAAAAAATGGCGAGGGTCCTCCTAGCTCGAGAGGAACATTCAGAGAATAGGCGACGGGGAGGCAGGTCAGGTCAGAAGAGGCTGCCCCTGGGAAAGGACCAATGTGCCTACTGCAGGGAGAATGGGCACTGGAAACGGGACTGTCCCAAGTTAAAAGGGGGCGCAGCTCCGGTCCTGGTAGAGGAGGAGACTCAATAGGGCCGTCGGGGTCCCTCAGCCCTCCAGGAACCCAGGCTAAAGTTAAAAGTCGGGGGGCAATTGATTGATTTTCTGGTTGACACAGGGGCAACCCATTCAGTAGTGCAGAAACCCGTTGGTCCAATGACAAGGGATACGGTGACTATTGTAGGGGCCACCGGGGCCACGTGCAGGTACCCTAAATCGGAAGGTCGAATTGTTGATCTAGGGAAGGGATTGGTAACACACTCCTTCCTAGTTATTCCCGAATGCCCTGACCCCCTGTTGGGACGGGACCTCCTGCACAAGTTAAGGGCCACCATTATATTCCCCGAAGCGGGGACCCCTGAAATTAGAACTGAAGGCAAGTTACTGCTGTCCTCACCCTTGGTGGAGGAGTATCGTCTGTTCACTGAACAACCTGCACAAAACCTCGCCCTCTTAGATTTATGGAGGGAGGAGATCCCCGGAGTATGGGCAGAATCGAACCCTCCGGGACTCGCTACTACCCAGGTCCCCGTGCATGTCCAGCTTACCAGCACGGCCCTGCCGATCAGAATAAGGCAATACCCTATAAGTCTGGAGGCTAGAAGGAGCCTCAGGGGGAGTATTCGGAAATTTAAGGAGGCAGGAATATTGAAACCCGTCCACTCCCCTTGGAATACCCCCCTCCTACCCGTCCGGAAAACTGGGACCTCGGAATACCGCATGGTACAGGACCTGAGGGAGGTGAATAAGCGAGTGGAAACCATACACCCCACTGTTCCCAACCCTTATACCCTCCTCAGCCTTCTGCCACCTGACCGAACCTGGTATTCGGTCCTAGATCTTAAGGACGCATTCTTCTGTATACCTTTGACTTGTCAATCACAGCTCCTGTTTGCATTCGAATGGATAGACATGGAGGAGGGGGAGTCGGGCCAATTGACCTGGACCAGACTGCCCCAGGGATTTAAGAATTCCCCCACCTTGTTTGACGAAGCTTTGAGTAGAGATTTGCAGGGATATCGATTTGACCACCCAACAGTAACGCTCCTCCAGTACGTAGACGACCTTTTGATTGCCGCCGGGAGTCGAGATGAATGCCTCCAAGCTACCAGGGACCTGCTGGTCACTCTAGGATCAATGGGGTACCGCGTGTCAAGCAGCAAGGCCCAGCTGTGCCAGGAGGAGGTCACTTACTTGGGATTCAGGATCAAGGACGGGACCAGGACGTTGGCCCAGAGCCGGGTCCAGGCCATCCTGCAGGTCCCAGCCCCGAAGACCAAGAAGCAGGTACGAGAGTTCCTGGGCACGGTCGGCTACTGCAGGCTCTGGATCCCCAGCTTCGCGGAGTTGGCACAACCCCTATACGCCGCCATCCGAGGGGCCGATGCCCCCCTACGATGGACCAGTACCGAAGAGGAAGCCTTCCAGCGGTTGAAAACGGCCCTGCTGCAGCCACCTGCTCTGGCCCTACCCGACCTGGACAAGCCCTTCCAGCTTTTTGTAGACGAGGCAGAGGGTGTTGCCAAGGGGGTGCTCATGCAGACTCTCGGCCCCTGGAAGAGACCAGTGGCGTATCTCTCCAGGAAACTGGACCCCGTGGCCGCCGGATGGCCCCGCTGTCTGCGGGCCATTGCAGCCGCCGCCCTCCTGTCCAAGGAAGCGTCGAAGTTAACCTTCGGGCAGAGTTTGGAGATCACCTCGTCTCACAACTTGGAGGGTCTCCTGCGCACGCCCCCGGACAAATGGCTGACCAATGCTCGAGTAACCCAATATCAGGTCCTGCTCCTGGACCCACCCCGGGTGATCTTCAAGCAAACTGCGGCACTTAATCCCGCAACCCTGCTGCCAGCAACTGACGACTCCTTGCCCCTGCATCACTGCGCGGACACCCTGGATGCCCTAACCACCACCCGCCCGGATCTGACCGACCAACCCCTTGCCGACGCTGAGGCCACGCTCTTCACTGATGGGAGCAGTTACGTGAAGGAAGGCCTGAGGTATGCGGGGGCGGCCGTGGTGACAACGGACTCCATCGTCTGGGCTGAGGCACTCCCGAAAGGGACGTCGGCCCAGCGGGCTGAACTTATAGCCTTAACCAAGGCGCTGGAATGGAGCAGGGGTAAGACTGTGAACATCTACACCGACAGCCGTTATGCGTTTGCTACCCTGCACGTACATGCAATGATCTACAAGGAAAGGGGACTGCTGACTGCCGGGGGCAAGGCCATCAAAAACGCCTCTGAAATTTTAGCTCTTCTAACGGCCATCTGGCTGCCAAAGCGTGTCGCCGTCATCCACTGCAGAGGACACCAACAAGGTGAATCGTTGGAAGCATTGGGAAACCGGCTGGCTGACAAGACAGCCCGGGAGGTCGCTAAGAAGTCACCGGCAATTCAGGCCTCCCTGTGCGACTCGCCCCGTACCCCAGTTGACTGGGTCCCAGTGGACACCCCACAATATACAAAAGAGGAAGAGGCTCTCGGCCAACGGCTTGGCGGAACCACTGACTCGACCGGCTGGTGGCGACTCCCTGACGGGCGGATCCTACTCCCAAAAGCAGTAGGGAGGCGGGTAGTCGAGCAGACCCACCGTGCTTCCCATCTTGGGGAATCCAAACTGGCCGCGGTCATACGAAAGCACTACCTCATCTGTGGCATCTACGGGGCAGTAAAAGACGTGGTGCGCAGGTGCGAGGCCTGCGCTCGGGTAAATGCACAATCCGTCCCTACCAGCTCGGCCGAGAACGTCCGCGACCGAGGACTGGCCCCCGGGGAACATTGGGAAATTGACTTTACCGAGATGACTCCGGCCCGGGGCGGCTACAAGTATTTGTTGGTCCTGGTGGATACCTTCTCCGGATGGGTGGAGGCTTACCCGGCGAAGGGGGAAACGGCTCAGATTGTCGTCAAGCACCTGGCAAATGATCTAGTCCCGCGATTTGGACTGCCACTTCGTATTGGGTCTGACAATGGTCCGGCTTTTGTCGCAAAGATAACTCAGCAGCTGGCCTCCGCGCTCCGGATCACCTGGAAACTACACTGTGCGTACCGGCCCCAGAGCTCTGGGCAGGTGGAAAGGATGAATCGGACTTTGAAAGAAACTATCACCAAATTAAAGATGGAAACTGGGGGTGATTGGGTCGCGCTTCTCCCCCAGGCCCTCCTCCGGGCCCGGTGCACACCAGGGAGGGAAGGCCTGTCCCCCTTTGAGATTGTCTATGGTCTGAGGCCCCCCCTGGTGCCCCGAGTCGGCCTTGACCAGCTTGCCGAAGTCACCCATCGGTCTTTGCTTAAGTCCTTACAGGCGCTGCAGGCTACGCGGTCCCTCGCCCGGACGACCTTGGCAGATCAGCAACCTGGGGCAGAGGTCCATCGAGGAAGGGAGCCCCTCTTCCATCCTGGGGACCTTGTCTACGTAAAGAGACTCGACCCCCGGCAGCTCGCTCCCCGCTGGGACGGGCCCTTCACTGTCGTTTTGAGCACTCCCACTGCCGTGAAGGTAGCTGGTAAGACCCCGTGGATCCACCACACCAGGTTGAAGGGTGCCCCAGAGTGCAGTGGAACATGGGGGATCGATCCTGCCTCCACCCCTCTCAAGTTAAAACTCTCCAGACGTTTGTCATAACCTTGTTCGGAGTCTGCTGTCTCTCTGGGGTTCTGGGGGGCCTCGGGCCCCCCCCTAACAAAGAGGCACTGATAGCGGCCCTGTGGGGACCCCCCTGTAACTGTCGAGGGGGCGTCCAAGAAACCGTACCCACCACCTATACGCGATCAGTTACTTGTGGCGGCTCGACGGCTTACCTAGTGTACAACAGGGGAGTGGGTGGAGGATATCATCAACATTGGGTATGCACTCGCCGACCTAAGGTACTACTGTCCCCGGACGGTCGACCTGGGCCCTGCCCAACGGCATGCCAGGTCACCTCCCAGATGCACTCCACTTGCTATAGCGCGGCCCAGCAGTGTAACCACACGGACGGGAGGGTCTATTTGACTGCCGTCCTACAGAGAACTTACAGTGGCTCCTTTGGGGGAGAAATGGACCACTCCAAATACGCCCAGGCCTCCTGCACGGGGACCGTCGGTCAGCCCGTCTGTTGGCCACTCCGGGCCCCCCTCCACATCTCTGATGGTGGGGGGCCGTCCGATCGCGTCCGGGAAGCGCAGGTGTCGGAACGCATGAAGGAGGTAATCCAGATCTTACACCCCTCGATCCGGTATCACCCCCTGGCGCTACCTAGGCCCCGGAGCCCCGGTCTGGACCCTCAGACTGCGGATATCCTCGCCGCCACCCATCAAACTTTGAATGCCACTAACCCTGCGCTGGCGGCAGATTGCTGGCTCTGCATGCCCTTCGGCCATCCTATACCCATTGCAGTGCCCGGGAGCGCGTCCAAAAACGCTTCCGTGTCCGGGCCCGATTCTCCTTACCCCGGAGGAAACTGTACTCACAACCTTCCCTTTCGTGTGCAGCCGCTTTGGGCCCCCCCTGTTCCCTGTTACCTTAGGTCAGGCCCCAACACCAGTATCGCCTTGGACGTAGGCTTCGCCTCTTGTAACTGCTCTCAAACCGTCAATGTCTCCGCCCCACTGTGCCCGGCCCCCGGTCGAGTCTTTGTGTGCGGTGGGAACTTGGCTTTCACGGCCCTTCCCGCCAACTGGACGGGTCTTTGTGTCCAAGCCTCCGTACTTCCTGACATCGACCTTATTCCAGGTGACGAGCCTATTCCACTCCCCAGCCTGGATTATATCGCCGGTAGACATAAGAGGGCCATTCAGTTTCTCCCCCTACTCCTGGGCCTTGGGTTGGCCGGTGCGGGCATGGGAGCAACGGGTCTAGGGGTGTCGGTCCACTCCTACCATAAATTGTCCACCCAGCTCATTGAGGATGTCCAGGCTCTTTCAGGCACCATCCGCGATCTACAGGACCAGATTGACTCCCTTGCTACAGTGGTCCTACAAAACCGGAGGGGCCTGGACCTGCTGACAGCTGAACAGGGCGGGATCTGCCTAGCCCTAAAGGAACATTGCTGCTTCTACGCTAACAAATCCGGGATCGTTCGGGACAAGATCCGCAAGCTCCAGGAGGACTTGGCCGTGCGGCGGCAGGAGCTGGCCAACAACCCCCTCTGGAGCGGCTTCAATGGACTCCTCCCTTATCTGCTGCCACTTCTGGGCCCCTTGTTTGCATTGATCCTTGTGTTGTCTATCGGCCCCTGCCTGTTCAGAACTGGAGCACGTATGCTCCAGGATAGGCTGCAAGCTATTAAAGTCCTGGCCCTGATGTCCCCGTATCAACCAGTGCCCCCTGAGGACCCCTCCCCCGCGTAACCTTGCGTTTGGCTTCTGTACCCACGCTTTGCTGAGCGGTCAAAGATTTGCCCTTCACTGACAAAAAGCAGTGGGGAATGTGAGGCTTGGAGAGGCTTGAGTCTCTACAACTAGACAGACCCCTATCTTGGGCAACCGGGCCAGTAACACAAGGAAGGAACATTTGCATAACCGCTCCTCAGACCCCTATCTTGGGCAACCGGGCCAGTAACACAAGGAAGGAACATTTGCATAACCGCTCCTCAGACCCCTATCTTGGGCAACCAGGCCAGTAACACAAGGAAGGAACATTTGCATAACCGCTCCTCAGACCCCTATCTTGGGCAACCAGGCCAGTAACACAAGGAAGGAACATTTGCATAACCGCTCCTCAGACCCCTATAAAGGGTGTAAAGTTGTGCTCGTGGGTGCTGCCGCTTCCCAAGCCTTACTATAAGGCGGTAGCCCAGATTTAAATCTGCAATAAAGCTGCGACCTGCCCTCTTTGGCGCTGGGCACGTCATCTAAATTTTGTCCGGTTGTCGTGCGATCCAATTTGTTGGTGGGTGGGATCCCGGGCCCTGGAGACCTTGATCAGGTTTCCTTACAACA

>ERV-Env-Tac2

TGTTGGGGACCACCTGTCATGTAAGGCCCCTGGCCTACCCCTGTAAAGGCACCGCACCCAGCCTGCCAGTTCCAGGAAGGGCCTAACCACGAGATGTCCTCATCAGCAGATGTTCCAACGGCGATAAGCTCCGGATAGCCCCTGAAGCAAACTTCCTGATTGCCGCACCTCCCCCCACGCTCTATATATACATGTAGTGTGCAAAAATAAAGTTGACTCTTGCCTCGCACCCACCTCGGTCTCCCTTCTCTTCTTCACCCGTCCCCTTTCAGCCGCTGGCGGCTTACTCAGGCAGGGTCCTCCTCGGCCCCCACTCGACCCGGCCCCGGTGGCACGGGCAAGTGGCGCCCAACGTGGGGCCCGAGGCACGGGACCCTGCCAGGCGGACCCCTGCATTCGTTATCTGAATCGACTCGTCGCGACCCCACATTTGACCGCTCTACGGAGCCCCTGCGTTTAGGTATGTTGAGCTTCGTCCGCCTCTGTATTCTCATGGGTTCCACGCTTTCTAAGGAGCAGGCCTTTATACTTGACCTCAAACAAGCTCTTAAGGAAAGGGGGGTCAAGGTTAAGAAGAAGGACCTTATAAATTTCTTTCTCTTCATTGATGAGGTTTGCCCTTGGTTCCTTGTAAGCGGACCAGAAATTCATCCAGGCAAGTGGCAGAAGGTTGGGAGGGATCTCAACAAAAAATTACAGACTGAGGGCCCGGAGGCAGTGCCCACCACGGCCTTCTCTTATTGGAGCCTCATTAGGGACATAGTGGAGGCCGCCTCTGGGGACCCGGACAAACGCCAGCTCCTTTCAGTGGCCGAGGTTTGTCTACGCCCTTTATCGCGGGCGGCTTCTGTCAGGTCATTGGCGTCGGGGGACCAGGATCCCCCCAGGCCTCCGTCCGTGGTCATCGATATCCCGCCAGCCCCAGCGAAACCACTCTACAACCCCCTCCCTGTGGAAGCGCAATCTATGACCGACCCCCTGACCCTCCCCATCCTTGCAAGACACGACCTCCCACCAACAAAAAACTCCGACCCCGATACCCTGGACCCTGGGGAGGAAGCCGAACTGGAGGATGAGGCCGCCCGTTATAATAATCCCGACTGGCCCCCTCCTCGGCTTCTCCCCCCCCCCCTTACCCAGTTCAAACCTGTGCTCCCGCATTTCTGCCCCCTGTCCCCACCCCCTCAGCCCTTGCGGACGCAAGAAACCAATTGTCCACTCAGGTCACTGAACTCCGAGAGGTCCTTGAGCTGCAGAAACAGTATGTCCAGCTCTCCTCGGAACTCTCCTTGTTACAAAAGACGCTCCGCAAGTCTGTCATCCTTTCCCCTGACGCCCCTCAGGCATCAAAAACCCCTACAAAGAGGAATAGCCCTAGCTCCCGGGATAAGAAATCAAGAGCGTTGCAGCCAATGGCATTTCCCGTCGTAACACGCTCCCAACAGTCTGAGCCCCCACGGCCTCCAGCAGGTGACCCCAAAGAATCCTCCGCCAGTGAGGATGAGGAGGGGGAGGAAGAAGGAGAGGGCGGGGGAGAGAGCGGTGAGGAAGCCTCGACAGGGCAGGAACGACCGGAGTACCGAAAAATGCAGTTTAAAACGTTAAAAGACCTTAATGCCGCCGTCAAATCTTATGGACCCAATGCCCCCTTTACCCTCTCCGCCCTCGAGGCCATGTCTCGCGGGGGATATCTCCTACCGGCAGAATGGCTCCGGGTGGTGCAGGCTGTCCTCACACGAGGCCAGTTTCTAACCTGGAAGGCAGACTTCTTTGATCGCTGCCAGTCCATAGCGGCAGTCAACCTTAAATCCCCGAGCACTCCTGCTGCTAGGTGGACCTATGAGAAACTGAGCGGGCAAGGGAGATACGCAGGAGAAAACAGGCAGCGCCATTTTCCCATAGGCCTCCTAGCCCAAACCACCAATGCTGCGCTTGCAGCTTGGCGTGCCCTGCCAACCGCTGGTTCCCCTCTTGCCCCCCTTAACAAAATAATGCAGGGAGCGCAGGAGGACTTCTCAGAGTATGTCAGCAGGTTACTGGAAGCTACTGAGCGGACCCTAGGGCATGAAGCTGCCAGTGATCAGCTTGTAAGACGTCTAGCATATGAGAATGCGAACAATACCTGCCGCTCCACATTACACGGTAAATGGAGAGATAAAACCCTGGACGAAATGATACGCTTGTGCAGGGACATTGACCCCTTTGCTTCAAGGGTGTCACAGGCCGTGCATCTGGCTATTGGGGCAGCCCTCCAGACAGGGGACCCGCAGAGAAACTGCTTTCGGTGCGGCCAGCCCGGCCATTTTGCCCGCCAGTGCCCGGAATCCTCCCCTACTACTCCTACGCTGTACCCAGATTCCCCCCCTGCTCCCGCTCCGTATAGGTCCCCTCAGCCGTTGACCCTCTGTCCTAGGTGCAGGAAGGGGAAACATTGGGCAAACACTTGCCGCGCCATTACGGATGTCGATGGTCGTCCCCTACGGGGAAACGGCCGGAGGGGCCAGCCCCGGGCCCCTCAGCCAATCCCCTTCGTTCGGGCCTCGGGGGACAGCCCGCCTGCACCCCGCCCTACAGAGCCACCTCGGGAAGTGCGGGAATGGACTTGTGTGCCACCTCCACCACAATACTAACTCCCGAGGGCGGGGTTCAGGTCGTCCCTGCGGGGGTGTACGGTCCCCCACCCCCGAATTTGTACTTCCTCATTCTTGGTCGCGCCTCGGCTGCCATCGGCGGCCTCGTTATTCATCCCACTGTGGTGGACAGTAATTACACGGGGGAGATCCACTTGCCTGTCTCGGCCCCGAAGGGCCCCATTGCTATTTCTCAAGGACAGCGCCTGGCTCAGGCCCTGCCGCTCCCCATACAGACACGCCTGTCTCGGCCCCGAAGGGCCCCATTGCTATTTCTCAAGGACAGCGCCTGGCTCAGGCCCTGCCGCTCCCCCTACAGACAACCTATCCCGCCCCCGTTACTAAACGAGGGCGGTCTCTGCCGGGCTCCTCGGACATATACTGGGCCCAGAGCCCCTGTATTCGTCGCGACCCCTCCCCTTTTTCTTTCTGACCCCGCTTCGGAACTCCTGCATTTGGGTTCCCGGGTAGCGATTGGATCCTCCCGTTCCTGATATCGGTCGTCACGGGCACAGCGGTGCGGGCTCCGGGAAGAGGTCGGCCCCGCGTTACCAGAAAGGACCCGCCCGAAAACCGTTGGCGTGAGTTGTCACCCCCTGCGCTCCTGATCGTTTGTAGACCACCATCCGGAGAGGACCCGGAGAGATGGATAACCCTCGAGAATGAGCATGCACTGATAACCCCGATTTGGGGGTTGCCTCGATCATCTGAATCCAGAGACTGCGGGCCGCATGGACCCTGAAGGTTACGTTCAGTGGGGACAGTGGGTTCTGGGAGGTTGTTTCGGTTTACCTCTTGTGCTTGGGATTGTGATATTCTTGCTGTACTTGTGCCATACGAAGTGCAGACCCCTTAGACAGGACTAGGTGACAAGATTGTAAAGAGAACCAAGCAGCAGAGGTGAGGGCACAGACACCATGATTTCGTGGCGCATGGTCCCTGTGCTGACCCTCATCTTCCTTGCCATGGCAACCGTGCTGTCTCTTCTCCTGCTGCTGCTTCCTGGGACCCAGGGCGGCTTCGAGACCCCCGACCGCGCCGCGCTCAGCCGTACCCTCTTTGGCAGCCCCTGCGACTGCAAGGGCGGTATGCTCTCCGTGCGCCCCGCGTCGTATACACGTTCGGTTGATTGCACCACAAAAATGGCCTACCTTGCCTACCGCCACACCATTACAGGGACCTCCAAGCAATCGTGGGAATGTGTAACCAAGCCCCGGGTAATCCCTGCAATTGGCGACCAGCCTGGGCTCTGCCCCTCCGGCTGCGTCTACCTCCAGGCCCTACACTCCACCTGTTACGATTCTGTCCAACGGTGCACGGGCCCGCAGGATCAGCCCTTGCCCACTGCCATACAGCAAAGGGACTATGCTGGCACCTTCGGAGGTGAGTGGGGGTCTGACCCCCATTCCAGCAAATATGCTCAGGCCCCCTGCGACAGGAACAATGTCGGAAAGACCGTTTGTTGGCCCCTCCAGGCCCCTATCCACCTCTCTGATGGGGGTGGCCCTACGGATCAGGTCAGAGAATCCCGGGTAAGTGCTCGTGTGGAGGAGATAATTAAAAGTCTTTATCCCTCCTTGCACTATCACACCCTCGCCTTGCCAAAACCCCGGGGTACGGACCTCGATGTCCACACCTCGGAGATCCTAGCGGCCACGCTCCGGGCCCTTAACCATACCAACCCAGACCTGGCTGCTAGCTGCTGGCTATGCATGACCCTCGGCACACCCATGCCCCTGGCCCTTGTGTCCGAGAACGCCTCCCTCTCGGAGAATTGCACGCTCAGTCCGCCTTTTAGGGTGCAGCCCGTTAGCCTCGACTCCCTCCCTCCCCCCTGTACCCAAGCCCCTTTTCAAAATTCCAGTTTTGACATCGATGTTGGGCGCGCCCCCTTTGTCAACTGCTCGGTGACTGTCAACCTCTCCTCCTCGGCTATGCGCTGCCCCAGACCGGGCCAGGTTTTCGTGTGCGGGGGTAATCAGGCCTTCACCGCGCTACCCCAAAATTGGACAGGCCTGTGTGTTCAGGCATCGCTTCTGCCGGACATTGACATTATTTCGGGTACTGAGCCCGTCCCCCTGCCCAGCTTGGATTACATAGCTGGTAGATCCAAGAGGGCTGTCGTACTCATCCCCTTGCTCGTCGGCCTCGGAGTTACAGGGGCTTTGGCCACTGGCTCCGCCGGGTTGGGGGTGGCCCTAGACTCATATCGAAAGCTGTCCACCCAATTAATCAGTGATGTGCAGACCCTCTCTGAGACTATTCACGATCTCCAAGACCAAATCGACTCCCTTGCGGAGGTCGTCCTGCAAAACCGGAGAGGGTTGGATCTGCTCACAGCAGAACAGGGGGGGATCTGCCTAGCCCTGCAAGAAAAATGCTGCTTCTACGCCAATAAGTCCGGCATAGTTCGGGACAAGATCAGGAAATTGCAGGAAGACCTGGTCAAGCGTCGTCGCGAGCTCTTTGAGAATCCCCTTTGGAGTGGCTTGCGTGGCGTCCTTCCTTATCTCCTTCCTCTCCTTGGGCCCCTGTTCGGTTTCCTCCTCCTTCTTTCCTTTGGGCCTTGGGCTTTTAACAAACTAACCTCTTTCGTTAAGTCTCAGATCGAGTCCTCTCTCAGGACGCCTGTCGGTGTCCACTACCACCGCCTCGATTCACAGGATGACCCTGTGGATTCTCTCGAAGACGGCTTTCGACTCTCTACTCTCGCGCAGCCTGATTCTCGGTGCGCTAGGATGTGGCGCTCCTTTCAGGAGAAATGCCACCTGACAGCTGTGAGACGGCCGATGACGGGAATATTGAGGCCCCATGGCAGACCCCAGGCGCGGCTGCAGTCGCACCCCATGACGGGAATAGAGCGGGGCCGCCAGGGCCAGGCTGCTGCGGGACCTCCCCGCTTAGCCTAAGACAGGGGCGCTGCCTATACGCTGCAGTCGCACCCCATGACGGGAATAGAGCGGGGCCGCCAGAGCCTGGTCCTCGCGGAGCCACCCCGCTTAGCCTAAGACAGGGGCGCTGCCCGCAGCCACTCTGCCCATGGAAATCAGCCTCATAGCCTAAGCCAGGCTTTCTCTATATACTCTGAGAGGGGGAGATGTTGGGGACCACCTGTCATGTAAGGCCCCTGGCCTACCCCTGCAAAGGCACCGCACCCCGCCTGCCAGTTCCAGGAAGGGCCTAACCACGAGATGTCCTCATCAGCAGATGTTCCAACGGCGATAAGCTCCGGAAAGCCCCTGAAGCAAACTTCCTGATTGCCGCACCTCCCCCCACGCTCTATATATACATGTAGTGTGCAAAAATAAAGTTGACTCTTGCCTCGCACCCACCTCGGTCTCCCTTCTCTTCTTCACCCGTCCCCTTTCAGCCGCTGGCGGCTTACTCAGGCAGGGTCCTCCTCGGCCCCCACTCGACCCGGCCCCGGTGGCACGGGCA

>ERV_Env-Tac3_consensus

GTTTGTCTGATATCCATTTTGTTTTCCCATCCCTTCCTCCCATCCCTTTGTACACCCTTCTTCACGGAAGAAGAATGCCCGCAAAAACTTCCCGCCACAAAACCTGCCTAACCAAGGCCTCCCCCACCCTGACCTGCCTGACAAAAGCCATCCCTGTTGTCCACTAGCGGACTTGACATGACTGACTCCATTTTGTGCAGGCTGAGAGAACAACTCCTTTTTGTATTGTCTGCAACAGATGCCTTCAAGGCCATTAAACAAGTAACACTTATCTATGTAAGGTCCTAGGTCAGATGACATCCTGACAAGACAGTGACAACAGAAGATAACAGAATTTACACACCTATCTATGCAAGGCCATATGGCAGATAACAACAACCTTTACAAGGCAGTAAAAACAGAGAATTTACAGCATCCTGTTGGTCCTAATTGGATTCCTGAAATTCCTAAATGCTCCCTCCCATGTTGCTGTAACTGAATAAAAGACGCAGTGAGAATGGGATCGGGGCTGCTTGATCTGGGTCCCTGGCCCCTTGCAGCCGCGCGCTAATAAAATCCACTTCTAAATTTCTACCTGGGTCTCACTCGCTGATTCTCGGCACAACATTTGGAGGCCCCAGCGAGATGCACGGTGTCCCGTGTCACCGTGGAGACCCAACCTGGGAGGACCTCGGCCTCGGGGGAAGGAGACCATTCTGCTCTGACTTCTAGGGGGGCCGGCCCCTGAGACGTTCCAGGGCCCCGGAACTCAAGGCTGAGAGGTCTAAACTGTCCCCTGACGCCGACTGATCCTCATTTCGGCCTGGTGGATCCTGGGATCGGCAACGATCAACTACTTCCGGAGGTAACCTGGTTTCTGTTTTCTGGAGGGAACGAGTGCCGGACGCGGCAGTTAGCGCTTTGCGCCTCGATTCCCCGGGCACCCCAAGACGTTGGGTCTCTGCCCGATTCTGGTTCTGGTTCTGGTTCCGCGGTTGTCTGTGAATCTGTTCTATATGTGGAAGCGTTGCCTTTAGGAAATTCTGTTTTACGATCCTCTGTCTGAAACCTCCTCGCTCACAGACTGCCAGGCAGGTTTCCACCTGGATCGGGGACGAATATCTTCAGGGCAACTTCTCCAATGTCTACTGGAAGGATGTGTTCGCCTCCAGGTTGTTGTTTGTTTGTGTCTCTGTGTTGTATGAATGGGAGGTTCAGCCAGCACGCCAGAAACGCCTTTGAACTGTATGCTCAGTCACTTTAAAAAAGGATACCGGGATGGGTATGATTATGGGATTACCCTAAAGAAACAGAAGCTCATTCTATTTTGCATGAATGAGTGGCCCACCTTCGGGGTGGGGTGGCCCCCTCAGGGTAGTTTTGATAAGCAAATTGTAAACAAGGTTTGGAGAATTGTGACTGGGACCCCTGGCCACCCCGATCAGTTCCCCTATATTGACGTTTGGCTGGACCTCATTACCCACCCCCCTCCCTGGTTGAAAAACTGCTCCCTGAAAAGGGGCATCCGAGTTCTTTTTGCCCAACCTAAAAAAGGGCCCCCTTCCAGGGACACCCCCCCCCAAAAAAAGTCCTCCAGGAGTCCTAGGAGGATGATCTTCCCCCTCCTTGTAACCCGGGCGCCCTTCGGGACGTCCCGCAGTTTGAGAGCCCGCCCTGGACTCTCAGAGAGAGTCGAGTGGCATAGGCCCCATTAAAGGTACCCAGATTCGAGTATTGGGATGGACCTCCAAAATATGGACGGGGGAGGTTACTTATCGTTGTACGTGTTTCCATAAGCGCTTAGTACCGTGGTCAGTGCACGGTTAAAGTTCTCTAGATATTAGAAAGTGACGGGAGTGTCTGTTTGACTTGAGGAAACGAAGGTGGGAAAAGGAAAAATCTCAGTCAAAACCGAGTGGTGGGAAGTCCCTCCCGGAAATTCCGGAAGGCAGTCCGATGAACTTTCAAAGTATAAGAGAAAAGAGAAGAAATTAATTATAAAAATTATTATTAAATAATTACATATTACTGCCTAACTTGGGCTGGCGCCCACCTCGGAGGGAACGTCTTCTGGCCAAAGTACGGATCCTCCGAGGACTGGGTTTGCCAAGAATTGAACATCTATGTAATTATGAGGGAACCCCGGAATGATGGGGAATTTGTGTATGCCGCAGTCTGGCGGCCGTCGGTGGGTAAATTTGTGCTGAAGGAAATAGATGAGAAGAAAGATAGAGGGGGAAGAAAGAGAAAAACCGGCACACCAAAGTGATGTTGCAGACTCCAAAACAACAGGAAAAGCAAAGAAAGATTATGATATAAATCGTGCAGATGGTGGCAAAGTTGATCATGACTTGTTACGCCTGCGGCCGGCAGGGGCATATGAAAAGAGAGTGTCCTCAGCGGGAAAAAGAAAAGAGTTCTAGGTACATGAAGGAAAAGGGAAGGTGAAAAAATTGCAGTTGAAGGAAATTGTCTGCTCAGGCATCTACACCCTGCGAAGCAGGTGTCCCATAAGGGGGGAATGAGGGGTGGAGAGATTGCCTCTGAAGGAAATTGTATGTGTATGAAGGTTAGAGTAAGTGTGTGTGAAGGAGAAAGAGAAGGGACAGGAGATAATTTTAGTAATTCCTTCGTGGAGGGGGAAGACTAGGGGGATCAGGAGCCCATAGAACAAGCCCACCCTGAGCCCTTGACAAATTTGAGGGTGGGCAGAGAAAAACAAGATTAACAGTATTATATCCTTTTGATAGGCACGGGCGCTACCCGATCGTCTCTAACCAGACAGCCGATCAGGGCCAGAATCGAGAGGGAAATCATTAGGATCTCGGGGGTGCAGGGGGAGAACTTCCCGGTCCCTGTTACAGAGACCTTAGAACTAGAATACCTAGGGTCAAACTTCTCAGGAAAATTCCTGATCATACCCGAAGCCGGGGTAAACTTGTTGGGAGGGCTCTTAACCTCGGCGGGAAAAACCATTAAAAATAAGGAGATTTTGTCTTTACTAGACGCGGTCTGGATGCCGGCCCAGGTGGCCAGCATCCATCGCCCCGGGCACCAGCACGGGGACTCACCCGAGGCCATTGGCAATCAGGCAGCGGATGAGGCTGCCCGAGAGGCGGCAAAGTCCCCTCCCAGCGTTGCCCCCTTATGCCCTGTTTTTGACCCTAATGATACACCGGCCCCTCACTACTCCCCCTCGGATGATTCCTTCGCAAAACAAAAAGGGGGGACTAGAGACGGGTCAGGATGGTGGGTCCTTCCAGAGGGAAGAATTTTTGTCCCTGAGGCAGTAGGGAGAGAATGGATCACGCGGCTACACCAGATCACCCATCTGGGGGCACGAAAGATGGGCTTGTTGTTGAGAGATAGGTACTTTATCCCACACTTGGACAGCCCCTTAGCTAGCATAACCACCCGGTGTGGAACATGTGCACAGGTAAATGCGAAGCAGGGGAAGGCTGCTCCCTCAGGAGTCAGATTGCCGGGATTGCAGCCAGGAGAAAACTGGGAGGTAGACTTTACAGAGGTGAAACCCCCCGCGGCAGGCTATCGATACCTCCTGGTATTTGTTGACACTTTTTCAGGGTGGGTAGAAGCTTTCCCAGTTAAACACGAAACAGCCATGGTAGTGGTGAAAAAGATTCTAAATGAACTTCTCCCGCGATTTGGTCTCCCACTGGGACTCGGGTCTGAAAATGGTCCAGCATTCATAGCCAAAGTGTCCCAAGGCATAGCCAAAGCTTTAGGAATAGAATGGAAATTACACTGTGCCTATCAACCACAGAGTTCAGGTCAGGTAGAAAGGACCAACCGAACTCTTAAGGAATTCCTCACCAAACTGGTCCTTGAAACTCAGGAAAATTGGGTCATGCTCCTCCCACTGGCCCTACTCCGAAGCCGATGTACCCCCAATAAGTCGGGTCTCGCACCTTTTGAGATTCTGTTTGGTAGACCCCCGCCAATCCTCCCTCTAATCAGGGAGGAACTCAGGGCGGACGCTACTAATTCTTCCTTGATTAAGTTCCGGCAGGGTCTCCAGAAAACACAGGGAACACTCCTGAAATCTGTCCGAAACGCCCTGCCAGTTCCCACCTCTGCACCCGCACACGCCTTCCAACCCGGGGACTCGGTCCTGGTCAAGAAATTCACCGCCTCCGGCTTGGAGCCTAAGTGGAAGGGCCCTTACACCGTCATCCTGACCACGCCAACAGCCGTCAAGGTTGACTCCGTTCCTGTCTGGCTCCATCACAGTCGAGTGAAACCTGCTGCGGCCCCGACATGGAAGGCGGAGGCACAGGCCGACCCCCTAAAGCTAAGACTCTCCCGCATTTCCTCCTCCTCGCCTCCCTAATGACCCCCTCTCCCTCGATGTCATTCAACCCTCATGCCCCAAAACCCAAAAACACCTGGACGATGAAAAAGGGGGATCAGGTACTCTGGACATTCTATGCAGAAGAAGGTACATGGACCCATAACCAAGGTTTGAATGATGGCCGGTATTTCCGGTTGGATTTATGCTCCCTCTTCCCCACCTCGATCGGAGGCGCTTCCCCTTGTAGTAATCCGTTTTCTGAAGTAAGAATGTGCCCGGGGTTCCTTACAGATGGCTGGGATGCCAGGTGCCTGGATAGGTCGTCCCATTTCTGCCCGAAATGCGCCTGTGTGACCGCCATTGTAGTATCCTCGTTCCGCGGGGCGGCTTGCGGGGCCAAAGAGGGCCGCGTGGGGACGGATCCGCACTTGACTATCCAAAAGGACCCAACCCCTGGATCCACAACCTTGTTTCTCACCCTTCGCAACCCTGAGAGTAGGTTCTGGAATACTCCCCATTCGTGGGGCATGAGATTAGATGGCAGAAATAGGGCCTGGGCGGACCCTGGGATTATCTTTACCATTGCTAAGCAATCCCCCGTAACATCCTACTTGCCAATCGGCCCCCTTGGGGAATTAGCTCTGCCCCCAAAAATCCTCCCTAGACCTCGGGCCCCGTCCTCCACTGTCCAGTCCTTACCACGGGCCCCGTCCTCCACTGTCCAATCCCATCAGACACCCGCCGGGGCAATTGGCTCTCAAGAGCCAGGTGAGGGGGAGTCCTCCAGCCAGAAACCCCCTCATCCTTCAGTCATGGGCCTCTTACAGGCCGTTTATGGGGTGGTCAACTCCACTCGACCAGACCTGGGCCTAAGCTGTTGGCTATGCATGGATGCCCAGCCTCCATACTATGTTGGAGTGGCTATCAATAACTCTGTGTCTCCCACCTCCGATTCTGACAACTGTGAATGGGACCAGCCAAGGTTGACTCTTGGGGATGTCCAGGGCTCTGGGGTTTGCTTAATCTCGGATAACACGAACCTCCATGCCTCCCCATACTCGCCTGTCTGCTCCCTCAATGTGATGGTCCGGTCCTCCTCCGGTTCTGCTTACTTCCCCGCCCCACCGGGCACCTGGTGGGCGTGTTTGGACGGAATCACTCGATGTGTTTCAGCCCGAGTTTTCCTTGCTCACCCCGGTGGCCCTCTCTGTGTGCTAGTCTCCATCGTCCCCAGAGTGTCCTTGTTGCCTGGCGCTGATGGGTGGGACCACTTTTCCCTGCGGGAGGATTGGTCCCTCCGTCATAAGCGGGCTGCCCCGCTGTTCATCCCCATTCTAGTGGGGTTGGGTTTAGCGGGTTCTGCCGCCCTGGGCACTACCGCACTGGTGCGGGGGGAGGCTAGCTACAGAGAACTCAGCACCCAGGTGGATATTGACCTCACCCACCTTGAGCACTCCATTTCCACTCTGGAGCGACAGGTTGACTCCCTGGCGGAGATGGTCCTCCAGAACCGGAGGGGTTTGGACTTATTGTTTCTGAGACAGGGTGGCCTCTGTGCCGCCCTGGGAGAGGCCTGCTGCTTTTATGCGAATAACTCTGGAGTTGTTCAGGAGAGCCTCTCTCTGGTGAGGAAAAATTTAGCAGACAGGCAAAGGGAGCGTGAACGGGCCGAAACCTGGTACCAGAGTCTTTTCCGGACATCCCCGTGGTTAACCACGCTTGTGTCTGCCCTAGCTGGCCCCTTGTTTCTCCTCGTAGTTGCCCTGCTCGTCGGACCCTGCTTAGTGAATCGCCTCCTAGAATTTGTTAAGTCCCGCATCAACTCTGTTAAGCTGCTCCTCATTAGGGATCTCCACTATCAATCCCTACAAACTGAGCCCGTTGGCCGGTATGACGATGTCGCCACAAACGTGTCAAGGGTTTGACACTCTGTCCATAAGAAGTGGGGAATGTTGTGATCGGGTTTGTCTGATATCCATTTTGTTTTCCCATCCCTTCCTCCCATCCCTTTGTACACCCTTCTTCACGGAAGAAGAATGCCCGCAAAAACTTCCCGCCACAAAACCTGCCTAACCAAGGCCTCCCCCACCCTGACCTGCCTGACAAAAGCCATCCCTGTTGTCCACTAGCGGACTTGACATGACTGACTCCATTTTGTGCAGGCTGAGAGAACAACTCCTTTTTGTATTGTCTGCAACAGATGCCTTCAAGGCCATTAAACAAGTAACACTTATCTATGTAAGGTCCTAGGTCAGATGACATCCTGACAAGACAGTGACAACAGAAGATAACAGAATTTACACACCTATCTATGCAAGGCCATATGGCAGATAACAACAACCTTTACAAGGCAGTAAAAACAGAGAATTTACAGCATCCTGTTGGTCCTAATTGGATTCCTGAAATTCCTAAATGCTCCCTCCCATGTTGCTGTAACTGAATAAAAGACGCAGTGAGAATGGGATCGGGGCTGCTTGATCTGGGTCCCTGGCCCCTTGCAGCCGCGCGCTAATAAAATCCACTTCTAAATTTCTACCTGGGTCTCACTCGCTGATTC

>ERV_Env-Tac4.1

GCTGTGGGGCTGTGGGGTGGGAGTGTGAGGGATGGAACCCAGTGAAGTTCCCTCATGAGACCCCAGGAGGGAAGGCAGAAGCTATCTCCCCGGGCCAGGTTTAGGTAACACCCAGAGCAGAACTGACTTGTGCTATGGCTATGGACCGGAGGGAGATAAAAGGAAATGAAACGGCCACATTACAGAACTTGCTTACTGAAGGTAGAGCCAGTCGGGTGAGGGGCCAAACCCGTGACCCCTTTTCAAGAGAAAAACAGGATGATTCAGCTTAAACCGCAAGGCCACTGCCAGCCTCTCTCCCGCTCCGCTTCACTTCCTTGTACAGGACCGCGCGTTGGGTACTAGCTGCCGCGATTAGATTGTACTCTGCACACAGTAAGCGCTCAATAAATACGATTGAATGAATGAATGTACGCCCCAAGTACCCAGCCAATGGAGAAAGGGGTTAGTCGGTGGGAGGGGTATGCGTTAGGGTATGCGTTAGGGCTATAAAAATCGGCTTCGCCCAATCAACGGGCGCACTCCCCGGCTGACGCTGCTTAAGGGACGGGGGTGGTGCCCTTTCTCATGAGAAAGAATAAAAGCTCTTTCTATACCTGGCTCGGACTCTGATTTCGATCGAGAGGGAGTCAACATCCCACACGGTTTGGGGGCTCGTCCGGGATCCTCTCCAAGCGAGGAGAAGCCTCCCTTGGCCTGGACTGACTGGCAGGACGTCCCGCTGAAATTTTCAGCGGCCCTCGGCCCGGCCTGGGAGCGGCTCTCCTCCGACGAGAAGCGACCCGAGGAGAGACCGAAACCGGGAGTAGAAACAGCTAGCGGGGACGGGACGGAAACCACTCGCGGGAACGATTGGTCACGTAAGTCTGTGCACAGACCTTGGGTGCTGACCCCGGGTGACCGGGCTGGGCGATAAAGGAACAGACCCTATAGACCGCCTGGAACGGGGAGTTTCGGCAGAAAAATAGGAAGTCAGTTCCCGCCCCGGAGAGACACGGGTCCCGTTGAAGTCATAAATTCGGAAACGGGGGTGTCTGCCTCGGTAGAAGAATGGGAAAATCCCAGGAAAAGTCGAGTGGAGGAAGCCCCTAATAAAGGTACCTAGATTAGAAAATTGGGATGAACTTCCAAAGTATAGGAGAAAAGAGAAGAAATTAATTATAAATATTATTATTAAGTAATTAAATACTGCTGCGCAGCTTGGGCTGGCGCCCACCTTGGAGGAAACGTCTTCTGGCCAAAGCACGGATCCTCCGAGGACTGGGTTTGCCAAAAGTTGAACATCTATGTAATTATGAGGGTACTCGGAATGAGGGAATGTGCATATGCCGCAGTCTGGCGGCCGTCGGTGGGTCAATTTGTGGGGAAGGGAATAGATGAGAAGAAAGATAGAGGGGAGAAGAAAGAGAAAAACCAGCAAGACAAAGTGATGATGCAGATTCTAAAGCAACAGGAAGAGGAAACAAAGGTTATGATGCAAACCGTGCAGACGGCGGCCAAGTCGATTATGACTTGTTATGCCTGAGGCCAGCCGGGAAATATGAAAAGAGAGTGTCCTTAGCGGGAAAAAGAAAAGAATTCCATGTGCATGAAGGAAACGGGAAAAAGAAAAGGGAAGATGAAGTAATTGCAGTTGAAGGAAATTGCTCAGGCATCTACACCCTGCGAAGCAGGTGGCCCATAAGAGGGGAATGATGGGTGGAGAGATTGCCTCTGAAGGAAATTGTATGTGTATGAAGGTTAGAGTAAGTGTGTGTGAAGAAGAAAGAGAAGGGACACGAGGGAATTTTAGTAACTCCCTCGAGGAGGAGGAAGATTAGGGAGGTCTGGGGTTCATAAAATAAGCCCACCCAGAGCCCTTGATAAATTTGAGGGTGGGCAGAGAAAAACAGGATTATACCTTTTTGATAGACACGGGCACTACCCGATCGTCTCTAACCAAACTGCCGATCGGGGCCAGAATTGGGAGAGAAACCATTATGATCTCTGGGGTAAAGGGAGAGAACTTTCCAGTCTCTGTTACGGAAACACTAGAATTGGAGTACTTAAGGTCAAGATTCTAGGGGAAATTCTTGGTCATACCCGAGGCTGGGGTAAACGTGTTGGGAAGGGATTTATCACCCGGATGTCTATCCGGCTCGTCCCTATGGGGTCCCAGATTGTTCCCCAAAGGGTATTATTATTAAGAGAGGAAGATAAGAATTGAATTGATCCAAGGGTCTGGGCGGGACCTCGAAATTGGGGAAAATTGAATATACCCCCTTTGAAGATTAAGTTACGGGAACTGGGGACTATGGTGAGAGTGAGACAGTACCCCATCTCACTTGAAGGAAGGTAAGGCCTAAAACCCGTGAATCAAGGCTTATTAGAAGACGGGCTACTGGAACCTTGTCAGTCTCCTTATAATTCCCCCATACTTCCGGTCAAGAAAGGGGATGGTATTTATCGATTGGTCCAGGACCTCCGGGAAGTAAATAAAATAGTACTCCCATCCCACCCCGTGGTCCCGGACCCCTATACCATCCTGGGAAATATCCCAGCTGAGAGCAAATGGTTCAGTGTTATTGATTTGAAGGACGCGTTTTGGGCATGTCCACTGGGCACGGACAGTAGAGACCTAGCAAAAAGCTGGATCCAGCCCGAATTTCCAGAATACTGCAGATTCAGCTCCCCAAGACTAAGAGAGAATTGCGGAAATTCGTAGGGCTGGTGGGCTACTGTAGATTGTGGATTGATTCTTATGCAACCCTGACAAAACTGTTATACCAACACTTGTTAGAGGAAGAACTGGATATCATCCTATGGGATGAGGAGGTTAGGAGCAATTTTAACGGGTTGAAAGGGACCCTGAGGTCACCCCCGCTGCTGGCGTTGCCCTCGCTGGAAAAACCTTTCCATTTATTTGTTAACGTGGATGGCTCTGGGAGTGTTAGCCCAACAGTGGGGGGCGGGGGGGCAGCGGAGGCCGGTGGCTTTTCTTTCCAAGGTCTTAGACCCAGTGGCTTGGGGATGGCCCACTTGTGTGCAGCCCATGGCCGCCACGGCCATCATGGTAGAAGAGAACAGGAAACTGACATTTGGGGGCAGCCTTGTTATCAGTGTTACTCATCAAGTCAGATCTATTCTAAATCAGAGGGCTGGGAGATGGTTGACAGATTCAAGGATCCTCAAATACGAGGCCATTCTACTCGAAAGAGATGACCTGGTACTCTCCCATGACACTAATCAGAACCCAAGCCGCGTTCCTGGTGGGAGGGCCTGATGTGGAGCTTGAGGAAGGGGGACACAGTTGCATGGAACTGATCGATTTTCAGACAAAAACCCGAGAGGATCTACAGGAGTCTCCCATCCCTCCCTGACAGTGTCAATTTGTTTATAGATGGCTCTTCCCGAGTGGTGGAAGGAAAACGGAGGAATGGCTATGCAATTATCGATGGAGACAAAACGGCTGTGGTAGAACTGGTGAATTTCCCAATCCTTGGTCAGCTCAGACCTGCAAATTATATGCACCAAGTCGGGCCCTGAAACTCCTGGAAGGCGGAGAAGGGAACATCTACACTGATTCCAAGTATGCCTGGGGGGTTGTACATGTGTTTGGAAAAATTTGGGAGAAAAGGGGAATGATGAATAATCAGGGGAAAGAGTTAGCCCATACCACCCTATTACAGCAGGTACTGAAGGATTTGCATCGGCCCAAAGCGCTGGCTGTTGTACATGTAAATGGCCACCAAAAGGGGAACTCTCGAGGCTAGGGGAAACCGCCTGGCTGACCAGGAAGCAAAAAGAGCTGGAGAACTCAAACAGGAAATAACCGAACCCATGTTGGTCCTGATTCCTACATTTACCACTAATTTAAAGCCGGTATCTCTATAGGAGAAAGAGGCAAGGAGGGCCCAAGATTTGGGAGCCCAGAAGGATGAGCGGGGGAGATGGATTCTCCCATACGGGAGGGAGGTCCTTAATGAAGCCACAACGAGACAGGTGTTACAACACCTACACCAGGGTAGCCATTGGGATGTCCAGAGCCTGTGTGATGCAGTACTGGTAAAAAACATCTGTCCTGGTATCTGTACACTTGCCCGGCAAGCAGTCGATGGCTGTATTATTTGTCAAAGGACGAATAAGAACAGCCAGCGACGCTGCCCCGCGGGTGGCCAGCCTCCGGGAATTTGACCATTCCAGAGCATCCAAGTGGACTTCACTGAGGTGCCCCCGGTGGGTAGGCTGAAATACCTACTGGTGGTAGTGGATCACCTCACGTTCTGGGTGGAGGCCTTTCTCCTGGCTCAGGCCACTGCTATTGCGGTGAGTTAAAGCCCTTTTGGAACAAATTATCCCTCGATATGGATTGGTAGAGAGAATTGACTCAGATCAAGGAACCCACTTCACTGCTAGAGTCCTCCAGTCTTTGATGATAGCCCTAGAAATTTCCTGGTACTTACACACTCCCTGGCACCCTCCGTCCTCAGGCAGGGTGGAAAGAATGAATCAGGAAATTAAGAAACAGCTCACCCGATTGGTGATAGAAATCCGACTTCCCTGGACGAAATGTTTACTGTTAGCCCTCCTCCGCATTAGGACCAAACCCTGCCGGGATATAGGATTATCTCCATACAAACTCTTATACGGTCATCCCTATCCAGCTAGACTGTCTCAACCTCCCCAGTGGGAGACCAAGGACAGGTTTCCAAGGGAGTACGTGCAGTCCCTGTCAAGCTATTTGTTTTCCCTACAGAAGAAGGGGATTGTCGCTCAAACCCCTCTGCTGGGATTTCCCGTACATAAGTTCCAGGCTGGCGATTGGATCCTCATTCGGGCGTGGAAGGAGAAGAAGCTGACGCTGACCTGGGAGGGCCCATTCCAGGTACTGCTCACCACGGACACGACAGTGCGGACTAAAGAAAGAGGCTGGACTCATACCAGAGTAAAGGGACCTGTCCGAAAACCATTGGAGTGGACTGTTGCCTCCTATGATCCTGACCAATTGAGGACCACCATTCGGAGGAGACTTGGAGAGACTGATGCCCACCCCCCCCCCTTAAATGAGGAAGGACCGGTAACCCTGCCATGACTCCTGAAGATTACCTCTGGTGGGGGCAGTGGGTTCTGGAGGCTTGCTTCGGTTTGCCTTTCATGGTTGGGGGAATATTTCTGGTGTGCCTGTATATTAGGAATTGTATTTCCATTAGGCAGGACTAGGTGATAGGATTGTAAAAAGAATCAAGTAGCAGAGGTGAGAGGACAGACACACATCATGATCTCATGGTGCATGGTCCCTGTGCTAACCCTCGCTTTCCTTACCCACCTATCCATCCCTGTGCCTTTAGGAAAAAGGACCCCGCCCCAGCCAAAGGAGGAAAATACGTTTACCCAGCTAGGGGAAACGATAGCTAGCACCCTTAATGTGACCAATTGTTGGATTTGCGGTGGTCCCCAGGAGTTAGAAAGTTGGCCTTGGGTCCCTATACCCCTGGAGCCGGCCTGGATCCTCAGTAACCAGTCAGAGGTGCGTAATGGTTCTGAGTTTTGGACATCCTCAGAGAAACATCGATGGCACCTGGCGGAGTTAGTTAAGGGCCAGTATTGCCTGAATCAGTCCGGAGGAGGGGAGTCGGTGGGAGAAAGTGACTGTGTCTGGACCTATTCCTCCACAGAAAAGAAACAGGTTAACTGTACTCCTTACCAGAACTGGTGGAACGATACTCACATTAGGTGTCACAATGGGAATTGGATGGGGTGTAATGTCACCGGTACACGATCATTCTGTGCAACCAAGATGTTGACTGGTAACTCGGCCGTAGGTTGTTCCCAGTTAACTGGGGCCCAGAGCGCACTGCCAGCGTGGTGTCTTAAGTATAATGAGACTGGAGTGAACTCCAATAGCACACACGTGATCTGGGAATGGCATAATTCAACCTGGTCAGGACATTTTCCTTCTTTCTGGAGTCCATGGAATCGGAGCTCCGACCCCCTTATCAAGACTTGCGTGGAGAATGCCTCCCTAGGTCTGTGGGAGTGCTGGTTTTTAGAGGAACAGCTCGGAGGACCCCTAGGGGGAGGACCTTTTGGGGATTGGGAAGGCATCTATTACCCCGTGGCCATAAACAGCACTGAACCCTTCACCATAAATTGGACTGTAATATCCCGGAACGCGCGGCCGGCACTAAGGGGCCATTATTGGATCTGCGGCCTAACAGCATATACACACCTGCCCGCAAATTGGTCGGGCAGCTGTTATATTGGCATAATTAGACCAAAGTTCTTCTTCCTGCCAGGCCAGGAGGGGAGTCACCTAGGAATCGACCTCTATGATGATTTGAGGGATGATGGAGGGAGAGAAAAGAGGTCTCCCGATACTTCGCTAACAAGTACTGGGGACGCAAACAGGTGGGGAGACAATTGGCCTCCGGAAAGAATAATAAGGACCTACGGTCCGGCCACGTGGGCTCAGGACGGGAGTTGGGGGTACCGAACTCCAATTTATATGCTCAACCGGCTAATTAGACTCCAGGCGGTCTTAGAAATTATTACTAATCAAACGGCGAGAGCCCTTGGTCTGTTAGCAGAACAAGCCACTCAAACCAGGGAGGCTGTCTTACAGCATCGGCTGGTTTTAGACTATTTATTAGCGGCTGAAGGGGGTGTGTGTGGAAAGTTAAACCTGTCAAACTGTTGTCTAAAAATTGATGACAATGGGAGGGTAGTCATGGAAATAGCTCAGGAAATAAGGAAACTAGCCCATGTTCCTGTGCAAACCTGGAAGACTCCTTTTGGTACCTCATGTATGTCTTGGCTTGGAGGCGCCTGGTGGAGACAAATTTTATGGTTCTTGCTTCTTGCTATAAGTGGAATAATACTCCTTCCTATCTGTCTGCCCTGTATGATGCAATTAATCACTAGGATCGTACAGAGTTCAATCCAGAAGATGTTGCAAGTATCAGGGAGTGGAGAAGTTAAAATTATGATCATGAGAGCTCAAGGATGGATCCCTTTAAATACTATGGATCCATCTGATTCTGATGAGCATCCTCCGGAAGAAATGATCGAGGAGGTGTATCAGCAATGGTGTGAGGAAACTTCAGGGAGGATAAAAGAAAGGGGGATTGTGAGGGATGGAACCCAGTGAAGTTCCCTCATGAGACCCCAGGAGGGAAGGCAGAAGCTATCTCCCCAGGCCAGGTTTAGGTAACACCCAGAGCAGAACTGACTTGTGCTATGGCTATGGACCAGAGGGAGATAAAAGGAAATGAAACGGCCACATTACAGAACTTGCTTACTGAAGGTAGAGCCAGTCGGGTCGGGGGCCAAACGCGTGACCCCTTTTTGAGAGAAAAGCAGGTTGATGCAGCTTAAACCGCAAGGCCACTGCCAGCCTCTCCCCCACTCCACTTCACTTCCTTGTACACGAGCACGCATTGGGTGCTAGCTGCCGTGATTAGATTGTACGCCCC

>ERV_Env-Tac4.2

GAATGTTGGGGACCACCTGTCATGTAAGGCCCCTGGCCTACCCCTGCAAAGGCACCGCACCCCGCCTGCCAGTTCCAGGAAGGGCCTAACCACGAGATGTCCTCATCAGCAGATGTTCCAACGGCGATAAGCTCCGGAAAGCCCCTGAAGCAAACTTCCTGATTGCCGCACCTCCCCCCACGCTCTATATATACATGTAGTGTGCAAAAATAAAGTTGTCTCTTGCCTCGCACCCACCTCGGTCTCCCTTCTCTTCTTCACCCATCCCACTTCAGGCAGGGTCCTCCTCGGCCCCCACTCGACCCAGCCCCGGTGGCAGGGGCAAGTGGCGCCCAACGTGGGGCTCGAGGCACGGAACCCTGCCAGGCGGACCCCCGATACGGATCGACTCGTCGCGACCCCACTCTCGACCGCTCTAAGGAAGGAGCCCCTGCACTCGTCGCGACCCCACCTTTGACCGCTCTAAGGAAGGAGCCCCTGCACTCGTCGCGACCCCACCTTTGACCGCTCTACGGAAGGAGCCCCTCCACTCGTCGCGACCCCACCCTTGACCGCTCTACGGAGCCCCTGCGTTTAGGAACGTTGAGACACGTCCGCCTCAGTTCTCCCTCGTCGCGACCCCGCCGCTCTGAGACAGGAGCCCCTGTATTCGTCGCGACCCCTCCCCTTTTTCTTTCTGACCCCGCTTCGGAACCCCTGCGTTTGGGTTCCCGGGTAGCGATTGGATCCTCCCGTTCCTGATATCGGTCGTCACGGGCACAGCGGTGCGGACTCCGGGAAGAGGTTGGCCTCGCGTTACCAGAGGGAAAGGACCCGTCCGAAAACCGTTACCAGAGGGAAAGGACCCGTCCGAAAACCGTTGGCGTGGGTTGTCACCTCCTGCGATCCTGATCGTTTGAAGACCACCATCCGGAGAGGACCCGGAGAGATGGATAACCCTCGAGAATGAGCATGCACTGATAACCCCGATTTTCCTCCTACTGGGGGTTGCCTCGATCATCTGAATCCAGAGACTGCGGACCTCATGGCCCCTGAAGGTTACGTTCAGTGGGGACAGTGGGTTCTGGGAGGTTGTTTCGGTTTACCTCTTGTGCTTGGGATTGTGATATTCTTGCTGTACTTGTGCCATACGAAGTGCAGACCCCTTAGACAGGACTAGGTGACAAGATTGTAAAGAGAACCAAGCAGCAGAGGTGAGGGCACAGACACCATGATTTCGTGGCGCATGGTCCCTGTGCTGACCCTCATCTTCCTTACCCCCCTATCCATCCCTGCGCCGGCAGGGAAAAAGATCACACCCTGGCAGAAGGAGGAGAATACGTTTACTCAGCTAGGGGAAACGATAGCTAACACCCTTAATGTGACCAACTGTTGGATTTGTGGTGGTCCCCGGGAGTTAGAGAGTTGGCCTTGGGTTCCTATACCCCTGGAGCCGGCCTGGATCCTCAGTAACCGGTCAGAGGTGCGTAACGGTTCTCAGTTTTGGACATCCTCAGAGAAACATCGATGGGACCTGGCGGAGTTAGTTAAGGGCCAGTATTGCCTGAATCAGTCCGGAGGAGGGGAGTCAGTGGGAGAAAGTGACTGTGTCTGGACCTATTCCTCCACAGAAAAGAAACAGGTTAACTGTACTCCTTACCAGAACTGGTGGAACGATACTCACATTAGGTGTCACAATGGGAATTGGATGGGGTGTAATGTCACCGGTACACGATCATCTTGTGCAAACAAGATGTTAACTGGTAACTCAGCCGTAGGTTGCTCCCAGTTCACTGGGGCCCAGAGCGCACTGCCAGCGTGGTGTCTTAAGTATAATGAGACTGGAGTGAACTCCAATAGCACACACGTGATCTGGGAATGGCATAATTCGACCTGGTCAGGACATTTTCCTTCTTTCTGGAGTCCATGGAATCGGAGCTCCGACCCCCTTATCAAGACCTGCGTGGAGAATGCCTCCCTAGGTCTGTGGGAGTGCTGGTTTTTAGAGGAACAGCTCGAAGGACCCCTAGGGGGAGGACCTCTTGGGGATTGGGAAGGCATCTATTACCCCGTGGCCATAAACAGCACTGAACCCTTCACCATAAATTGGACTGTAATATCCCGGAACGAGCGGCCGGCGCTAAGGGGCCATTATTGGATCTGCGGCCTGACAGCATATACACACCTGCCAGCAAATTGGTCGGGCAGCTGTTATATTGGCATAATTAGACCAAAGTTTTTCTTCCTGCCAGGCCAGGAGGGGAGTCACCTAGGAATACACCTCTATGACGATTTGAGGGATAGTGGAAGGAGAGAAAAGAGGTCTCTCGATACTTCGTTAACCAGTACCGGGAATGCAAATGGGTGGGGAGACGAGTGGCCTCCGGAAAGAATAATAAGGACCTACGGTCCGGCCACCTGGGCTCAGGACGGGAGTTGGGGGTACCGAACTCCGATTTATATGCTCAACCGGCTAATTAGACTCCAGGCGGTCTTAGAAATTATTACTAACCAAACAGCGAGAGCCCTTGGTCTGTTAGCAGAGCAGGCCACTCAAACCAGGGAGGCTGTCTTACAGCATCGGCTGGTTTTAGACTATTTATTAGCGGCTGAAGGGGGTGTGTGTGGAAAATTAAACCTGTCGAACTGTTGTCTAAAAATTGATGACAATGGAAGGGTGGTCATGGAAATAGCTCAGGAAATAAGGAAACCAGCCTCATAGCCTAAGCCAGGCGTTCTCTATATACTCTGAGATGGGGAGATGTTGGGGACCACCTGTCATGTAAGGCCCCTGGCCTACCCCTGCAAAGGCACCGCACCCCGCCTGCCAGTTCCAGGAAGGGCCTAACCACGAGATGTCCTCATCAGCAGATGTTCCAACGGCTATAAGCTCCGGAAAGCCCCTGAAGCAAACTTCCTGATTGCCGCACCTCCCCCCACGCTCTATATATACATGTAGTGTGCAAAAATAAAGTTGTCTCTTGCCTCGCACCCACCTCGGTCTCCCTTCTCTTCTTCACCCATCCCACTTCAGGCAGGGTCCTCCTCGGCCCCCACTCGACCCAGCCCCGGTGGCAGGGGCAGAGGAA

>ERV_Env-Tac4.3

CTTGTGAGGGATGGAACCCAGTGAAGTTTCCTCATGAGACCCCAGGGGGGAAGGCAGAAGCTATATCCCCGGTCCAGGCTGGGTTACACCAGATACGAAGACCCGGAAGGAGATAAAGGGAAAATAAAACAGCCACATTGCAGGACCTGTTTACAAAAAAACAGGATGCAGCTAACCGAAAAACAGGATGCAGGTTAAACCGCAAGGCCACTGTCAGCCTTGTACGTGAGCGCGCGTTGGGTGCTAGCTGCCGCGATTAAATTGTATGCCCTAAGTATCCAGACAATGGAGAAAGGGGATAGTCGGTGGGAGGAAGAGGATGCGTTAGGGCTATAAAGATCAGCTTCAATCAACAATCAGCTTCAGCTACAATCAACAAGCGCACTCCGTGGCTGATGCTGTTTCAGCGACGGGGGTAGTGCCCTTTCTCATGAGAAAGAATAAAGACTCTTTCTGATCCTGACTCGGACTCTGATTTGGTGGGTAGAGGGAGCCGCTATCCCACACAGTTTGGTGGCTCGTCCAGGATCCTCTCTGAACGGGGAAAGCCTTCCTTGGCCTGGGCTGACTGGCAGGACGTCCCGCTGAAATTTTCAGTGGCCCTCGGTCTGGCCTGGGAAGAGCTCTCCTCTGACGAGAAAAGACCCGAGGAGAGACCAGAGCCGGGATCAGAAACAGCCAACGGGGGAACGACGGCGGTCACTCGCGGGAACGATTGGTCACGTAAGTCTGTGCACAGACCCAGATGGGATCTGGGTGACCGGGCAGGGTGGAGAGGTGGTCCTGTTGAGGCTGCAGAGTTGGACTCCGGCCAAGAGCACAGGAGAGAAAAGAGCCTTCTCCACCCGGGGCGGTCCTGATTGAGTCTGTTTGAGCGACGGCTCCAGGCAAGAGCACAGGAGAGATAAGAGCCCCAGCTTCCCAGAGAGTCGAGTGGCATAGGCCCCATTAAAGGTACCCAGATTCGAGTATTGGGATGAACCTCCAAAATATGGAAAAATTATACAATACATTCTATTGATATTTGCTCCCCCCCCCCCCCCCATCTAAGCTGTAAACCTGTTGTGGATGGGGGAGGTCACTGTTTATCTGTTAATCGTTGTACGTGTTTTTGTAAGCGCTTGGTACCGTGGTCTGTGCACGGTTAACGTTCTCTAGATATTAGAAAGTGTTGAGAGTGTCTATTTGACTCGAGGAAACAAAGGTGGGAAAATGGGAAAATCTCAGTCAAAGCCGAGTGGTGGGAAGCCCCTCCCGGAAATTCCAGAAGGCAGTCCGTTGAGGGCCAGGTTGGATGACTGGAATGAACTCCCCAGATATAGAGGGAAAGATAAAAGAAAAATGATTACTTATTGTTGTTTAATATGGGCGGGTGTCCAACTCGGGAAAGACGTCCTCTGGCCAAAATATGGATCCCCCGAGGACTGGGTTTGTCAAAAGCTAAATACTTATGTGAATATGAAGAAATCCCCGGAGTGAAGAGGAGTGTGCCTATGCCGCGCTCTGGATGCCATTGGCAGGTCAATTTGTGGTAAAGGGAGCAAGAGAAAAGGGGCATGGAGAGGAGAAGCCCACCCCTTGGGATCCTCTCGCTTTTCTCCCTCCCCCTTACGCCCCCCCTTCCATCGGCACAAGTGGCTGCTCCCCTTTCCCCTTCTTCTGCTTCGGCTCCAAATCCGACAGCCGAGGGAGTGAAAGGGGAAGGGGACCCGATCGATTTAAAGGAACCAAGCCAGCCGGCGCACACTCCTCTTCGCCATGAGTTAAAACGGTTACTAGAAGACCAAGAGAATTTCCCGGCTTTCGAGCAAATCTACCCACCAATACCCCCTCAGGGAGGTTCCTTTGGCTCAAGGGGGAATCGGGTATGTCAACGCTCCCCTTAGCAGCATGGAGGTTAGGAATTTTAAAAGGGAAATGAAGGTTTTAATAGAAGACCCGGAAGGGGTTGCGGAACAAATCAACCAGTTTTTCAGCCCCAACCTGTACACCTGGGCTGAGTTAATGGCAATCCTAGACATACTCTTCACTGGGGAGGAACAGGGACAGATAAGAAGGGTGGCCCTGAGAGTCTGGGATGACGCACATCCCCTTGCCCAGGGAGGACTGAGGGGGGAACAGAAATACCCCCTCCAGGACCCCCGTTGGGACCATAACGATGCCGCTCACCGGGACCACATGAGGGATCTGCGGGGGATAATAATTCAAGGAATAAGGAACGCAGTCCCCAAAAATCAGAACATGAATAAGGTTACCGGGATCAGGCAGGATAGAGAAGAGACACCATCGACCTTCCTCAATAGGCTGAAAGAGCATATGAGGAAATATTCTGGGCTCGATCTATCTGATCCCACTGCCCAGAGCCTCCTGCGGGTGTACTTTGTCATGAACTCCTGGCCCGATATTAACAAGAAACTGCAGAAAATAGAGGGGTGGCAGGCGAGGCCACTGGATGAGCTATTAGGAGCTGCCCAAAAAGTTTACGTGAGTCGTGGGGAAGAGGAGAAGAAAGAAAAAGACAGGCCAGCCAAAGTGATGTTGCAGACTCTAAAGCAACAGGAGGAGCAAACAAGGGTTATGGTACAGACCGTACAGACGGTGGCAGAAGCAGTTAGGGGACCCTATGGAAGAGGGAGAGGGCGCAGGCACCCTGGGATGGGAGAGGCTCCAAGGAAAGGAGAATTCCCTCCTCGTGCCCCACTAACTTGTTTTGGCTGTGGCCAGCAGGGGCATATGAAAAGGGACTGTCCCCAGCAGGAAAAAGAACAAAGAATTTACTCCCTTGTGGAGAAGGAAGATTAGGGAGGTCAGGGGTTCATAGAACAAGCCCACCCTGAGCCCTTGATAATTTGAGGGTGGGCAGAGAAAAACAAGATTATACCTTTTTGATAGACACGGGCGCTACCCGATCGTCTCTAACCAAACTGCCAATCGGGGCCAGAATCGGGAGGGAGACCATTATGATCTCAGGGGTGAAGGGGGAGAACTTCCCGGTCCCTGTTACGGAGACCTTAGAACTGGAATACCTAGGGTCAAACTTCTCGGGAAAGTTCCTAATCATACCCGAAGCCCGGGTAAACTTGTTGGGAAGGGATTTGATCATCTGGATGTCTATCCGGCTTGTCCCAATGGGGTCCCAGATTGTTCCCCAAAGGGTAGTATTATTACAAGAGGAAGATAAGAATCGAATTGATCCAAGGGTCTGGGCGGGACCTCAAAATCGGGGAAAATTGAATATCCCCCCCTTGAAGATTAAGCTACGGGAACCGGGGACTCTGGTGAGAGTAAAACAATACCCCATCTCACTTGAAGGAAGGCAGGGCCTAAAACCTGTGATCCAAGGCTTATTAGAAGACAAGCTACTGGAACCTTGTCAGTCTCCTTATAATTCCCCCATACTCCCGGTCAAGAAAGGGGATGGTACTTATCGATTAGTCCAGGACCTCCGGGAAGTTAATAAAATAGTACTCCCATCCCACCCTGTGGTCCCGGACCCCTAGACCATCCTGGGAAAGATCCCAGTGGAGAGTAAATGGTTTAGTGCTATCAATCAATCAATCAATCAATCAATCGTATTTATTGAGCGCTTACTATGTGCAGAGCACTGTACTAAGCGCTTGGGAAGTACAAATTGGCAACATATAGAGACAGTCCCTACCCAACAGTGGGCTCACAGTCTAAAAGGGGGAGACAGAAAACAAAACCAAACATACTAACAAAATAAAATAAATAAGATAGATATGTACAAGTAAAATAAATAAACAAATAAATAGAGTAATAAATATGTACAAATATATATACATATATACAGGTGCTGTGGGGAAGGGAAGGAGGTAAGATGGGGGGATGGAGAGGGAGACGAAGGGGAGAGGAAGGAAGGGGCTCAGTCTGGGAAGGCCTCCTGGAGGAGGTGAGCTCTCAGCAGGGCCCTGAAGGGAGGAAGAGAGCTAGCTTGGCGGATGGGCAGAGGGAGAGCATTCCAGGCCCGGGGGATGACGTGGGCCGGGGGTCGATGGCGGGACTGGCGAGAACGAGGTACGGTGAGGAGATTAGCGGCAGAGGAGCGGAGGGTGTGGGCTGGGCTGCAGAAGGAGAGAAGGGAGGTGAGGTAGGAGGGGGCGAGGTGATGGAGAGCCTTGAAGCCCAGGGTGAGGGGTTTCTGCCTGATGCGCAGATTGATTGGTAGCCACTGGAGATTTTTGAGGAGGGGAGTAATATGCCCAGAGCGTTTCTGGACAAAGATAATCCGGGCAGCAGCATGAAGTATGGATTGAAGTGGAGAGAGACACGAGGATGGGAGATCAGAGAGAAGGCTGGTTCAGTAGTCCAGACGGGATAGGATGAGAGCTTGAATGAGAAGGGTAGCGGTATGGATGGAGAGGAAAGGGCGGATCTTGGCAATGTTGCGGAGCTGAGACCGGCAGGTTTTGGTGACGGCTTGGATGTGAGGGGTGAGTGAGAGAGCGGAGTCGAGGATGACACCAAGGTTGCGGGCTTGTGAGACGGGAAGGATGGTAGTGCCGTCAACAGTCATGGGAAAGTCAGGGAGAGGGCAAGGTTTGGGAGGAAAGACAAGGAGTTCAGTCTTCGACATGTTGAGCTTAAGGTGGCGGGCAGACATCCAGATGGAGATGTCCTGAAGGCAGGAGGAGATTCGAGCCTGGAGAGAGGCGGAGAGAGCAGGGGCAGAGATGTAGATCTGGGTGTCATCAGCGTAGAGATGATAGTTGAAGCCATGGGAGCGAATGAGGTCACCAAGGGAGTGCGTGTAGATTGAGAACAGAAGGGGACCAAGCACTGAACCTTGGGGAACCCCCACAGTAAGAGGATGGGAGGGGGAGGAGGAGCCTGCAAAAGAGACTGAGAAAGAACGACCAGAGAGATAAGAGGAGAACCAGGAGAGGACAGAGTCTGTGAAGCCAAGGTCAGATAGTGTGTTGAGGAGAAGGGGGGGGTCCACAGTGTCAAAGGCTGCTAAGAGGTCGAGGAGGATTAGGACAGAGTATGAGCCGTTGGATTTGGCAAGCAGGAGGTCGATTTATCGATTTGAAGGACGCGTTTTGGGCATGTCCACTGGACCCGGACAGCAGAGACCTATTTGCCTTTAAATGGGAAGATCCGGACTTGAGTAGGAAACAACAACTAAGGTGGTCTGTCCTTCCCCAAGGATATACTGAGTCGCCTAACCTTTTCGGCCAGGTATTGGAGACCGTCCTCTAGGGTTTCCAGTCCTCCCCTCAGACTCTGGTTCTGCAATATGTTGATGACTTATTGATAGCCAGACCGAAGAGGGAGGATGTGAGCAAAACTTCTACCGAGCTTCTAAATTTCTTGGGGGACCGGGGACTGAGGGTCTCCCAGAAAAAGATGCAACTTGTGGTAAAAGAGGTGAAATATTTAGGCCACCTCATTAGCGAAGGTAGCAAGAAACTAGATCCAGCCCGAATTTCCAGAATACTCCAGATTCAGCTCCCCAAGACTAAGAGAGAATTACGGAAATTCCTGGGGCTGGTGGGCTACTATAGGTTGTGGATTGATTCCTATGCAACCCTGACAAAACCGTTATACCAACACTTGTTAGAGGAAGAACCGGATATCATCCTCTGGGATGAGGACAGTAGGAGCAGTTTTAACGGACTGAAAGAAACCCTGCGGTCACCCCCGGTGCTGGCGTTACCCTTGCTGGAGAAACCTTTTCATTTATTCGTTAGTGTGGACAGAGGGGTGGCTCTGGGAGTGTTAGCCCAACAATGGTGGGGGCAGCGGAGGCCAGTGGCTTTTCTTTCCAAGATCTTAGATCCAGTGGCTTGGGGGTGGCCCACTTGTTTGCAGGCCGTGGCCGCCACGGCCATCATGGTAGAAGAGAGCAGGAAACTGACATTTGGGGGCAGCCTGGTTGTCAGTGTTCCTCATCAAGTCAGATCTATTCTAAATCAGAGGGCTGGGAGATGGTTGACAGATTCAAGGATCCTCAAATACGAGGCCATTCTACTTGGAAGAGATGACCTGATACTCTCCCATGACACTAATCAAAACCCAGCCACCTTCCTGGTGGGAGGGCCTGATGTGGAGCTTAAGGAAGAGACACATAGTTGCATGGAACTGATTGATTTTCAGACAAGAACCCGAGAGGATCTACAGGAGTCTCCCATCCCTGACAGTGTCAATTTGTTTATAGGTGGTTCTTCCCGAGTGGTGGAAGGGAAACGGAGGAATGGCTGTGCAGTTATCGATGGAGACAAAATGGCTGTGGTAGAACTGGGTAAACTTCCCAATCCTTGGTCAGCTCAGACCTGTGAATTATATGCACTAAGTCGGGCCCTGAAACTCCTGAAAGGCAGAGAAGGGGATATATATACAGACCCCAAGTATGCCTGGGGAGTCATACATGTATTCGGAAAAGTTTGGGAGGAAAGAGGCATGATCAATAGCCAGGGAAAAGAGTTAGCCCATACCACTCTATTACAACAGGTACTGAAGGATTTACATCTACCCAAAGCCCTGGCTGTAGTACATGTAAATGGCCACCAAAAGGGAAGCTCTTTCAAGGCTAGAGGGAACCGTCTGGCCGACGAGGAGGCAAGAAGGGCTGAAGAACTCGGGCAAAGAATAACCGACCCTATATTGGTCCTGATTCCTACGTTTCCTTCTAATTTGATGCCTGTATCCCTATCGGAGAAAGAGGCAAATGGGGCCCAAGATTTGGGAGCCCAGAAGGATGAGCAGGTGAAGTGGGTTCTCCCAGATGGAAGGGAGGTCCTTAATGAAGCCACAACAAGACAGGTATTACAGCACCTGCACCAAGGTAGCCATTGGGATGTCCAGAACCTGTGCGATACAGTACTCGGAAAATACATCTGTCCTGGTATCTATACACTCGCCCGGCAAGTAGGAGCTGGCTGTATTATTTGTCGGAAGACGAATAAGAACAGCCAGCGACGCTGCCCCGCTGGTGGCCGGCCTCCGGAAATTCGACCATTCCAGAGCATCCAAGTGGACTTCACCGAGGTATCCCCGGTGGGTAGGCTGAAGTACTTATTGGTGGTAGTGGATCACCTCATGTCCTGGGTGGAGGCCTTTCCCCTGGCTCAGGCCACTGCTACTGCGGTGAGTAAAGCCCTTTTGGAACAAATCATCCCACAATATGGATTGGTAGAGAGAATCGACTCAGACCAGGGAACCCATTTCACTGCTCGAGTCCTCCAGTCTTTGATGAAAGCCTTAGAAATTTCTTGGGATTTGCACACCCCTTGGCACCCTCCGTCCTCAGGCAGGGTGGCAAGAATGAATCAGGAAATTAAGAAACAACTCACCCGATTGATGACAGAAACCCAACTTCCCTGGATAAAATGTTTACCGTTAGCTCTCCTCCGCATCAGGACCAAGCCCCGCCGAGATATAGGATTATCTCCATACGAACTCTTAAACTGTCATCCCTATCCAGCTAGACTGTCCCAACCTCCCCAGTGGGAAACTAAGGACAGGTTTTTAAGGGAATATGTGCAGTCCCTGTCGAGCCATTTGTTTTCCCTACAGAAGAAGGGGATTATCGCTCAAACCCCTCCACTGGGATTTCCCATACATAAGCTTCAGGCTGGCGACTGGATCCTCATTCGGGTGTGGAAGGGGGAAAAGCTGATGCCGACCTGGGAAGGCCCGTTCCAGATATTGCTCACCACAGACACTGCGGTGCGGACTAAAGAAAGAGGTTGGACTCATCATACCAGAGTAAAAGGACCTGTCCGAAAACCATTGGAGTGGACTGTCGCCTCCTGCGATCCTGACCATTTGAAGACCACCATCCGGAGAGGACCCGGAGAGACGGATATCCCTCGAGAATGAGAACGAACTGGTACCCCCGATTTTCCTCCTAATAGGGGCTGCCTCAATCATCTGAATCCAGAGACTACGGACCTCATGACCCCTGAAGGTTACTTTCAGTGGGGACAGTGGGTTCTGGGAGCTTGTTTCGGTTTACCTTTTGTGGTTGGGGGTATAATATTCTTGCTGTGCCTGTACCTTAAGAAGTGTAGACCCATTAGGCAGAACTAGGTGACAAGATTGTAAAGAGAACCAAGCGGCAGAGATGAGGGCACAGACACCACGATTTCGTGGCGCCTGGTCCCTGTGCTGACCCTCATCTTCCTTACCCCCCTATCCACCCCTGCGCCGGCAGGGAAAGAGATCACACCCTGGCGGAAGGAGGAGAATACGTTTACCCAGCTAGGGGAAACGATAACTAACACCCTTAATGTGACCAACTGTTGGATTTGTGGTGGTCTCCGGGAGTTAGAGAGTTGGCCTTGGGTTCCTATACCCCTGGAGCCGGCCTGGATCCTCAGTAACCAGTCAGAGATGCGTAACGGTTCTAAGTTTTGGACATCCTCAGAGAAACATCAATGGGACCTGGCGGAGTTAGTTAAGGGCCAGTATTGCCTGAATCAGTCCGGAGGAGGGGAGTCAGTGGGGGAAAGTGACTGTGTCTGCACCTGTTCCTCCACAGAAAATGAACAGGTTAACTGTACTCCTTACCAGAACTAGCGGAACAATACTCACATTAGGTATCACAATGGGAATTGGATGAGGTGTAATGTCACTGGTACACGATCATTTTGTGCAATCAAGATATTGACTGGTGACTCGGCCGTAGGTTGCTCCCAGTTCACTGGGGCCCAGAGCGCACTGCCAGCATGGTGCCTTAAGTATAATGAGACTGAAGTGAGCTCCAAGAGCACACAAGTGATCTGGGAATGGCATAATTCGACCTGGTGAGGGCATTTTCCTTCTTTTTGGAGTCCATGGAATCGGAGCTCCGATCCCCTTATCAAGACTTGCGTGGAGAATGTCTCCCTAGGTCTATGGGAGTGTTGGTTTTTAGAGGAACAGCTCGGAGGACCTTTGGGGGGGGACCTCTTGGGGACTGGGAAGGTGTCTATTACCCCGTGGCTATAAACAGCACAGAACCCTTCACCATAAGTTGGACTGTAATATCCCGGAATGCGCGGCTGGCGCTAAGGATCCATTATTGGATCTGCGGCCTGACAGCATATACACACCTGCCAGCAAATTGGTCGGGCAGCTGTTATATTGGCATAATTAAACCAACGTTTTTCTTCCTGCCAGGCCAGGAGGGGAGTCACCTAGGAATACACCTCTATGACGATTTGAGGGATAGTGGAGGGAGAGAAAAGAGGTCTCTCGATACTTCATTAACAAGTACCGGGAATGCAAACGGGTGGGGAGACGAGTGGCCTCCGGAAAGAATAATAAGGATCTACGGTCCGGCCACCTGGGCTCAGGACGGGAGTTGGGGGTACCAAACTCCGATTTATATGCTCAACCAGCTAATTAGACTCCAGGCGGTCTTAGAAATTATTACTAACCAAACAGCAAGAGCCCTTGGTCTGTTAGCAGAGCAGGCCACTCAAACCAGGGAGGCTGTCTTACAGCATCGGCTGGTTTTAGACTATTTATTAGAGGCTGAAGGGGGTGTGTGTGGAAAATTAAACCTGTCGAACTGTTGTCTAAAAATTGATGACAATGGGAGGGTGTTCATGGAAATAGCTCAGGAAATAAGGAAACTAGCCCATGTTCCTGTGCAAACCTGGAAGACTCCTTCTGATACCTCATGGATGTCTTGGTTTGGAGGCGCCTGGTGGAGACAAGTCTTATGGTTCTTGCTTCTCGCTATAAGTGGAATAATACTCCTTCTTATTTGTCTGCCCTGTATGATGCAATTAATCACTAGGACAGTACGGAGCTCAATCCAAAAAATGTTGCAAGTATCGGAGAATGGAGAAATTAAAATTATGATCATGAGAGCACAAGGATGGATCCCTTTAAATACTATGGACCCATCTGATTCTGATGAATATCCTCAGGAAGAAATGATCGAGGAGGTTTATCAGCAATGGTGTGAGGAAACTTCAGGGAGGATAAAAGAAAAAGGGGGGATTGTGAGGGATGGAACCCAGTGGAGTTCCCTCATGAGACCCCAGGAGGGAAGGCAGAAGCTATCTCCCCGGGCCAGGCCGGGTTACACCAGATACGAAGAACCGGAAGGAGATAAAGGGAAAGTAAAACAGCCACACTGCAGGACCTGTTTACAAAAAAACAGGATGCAGCTAACCGAAAAACAGGATACAGGTTAAACCGCAAGGCCACTGTCAGCCTTGTACATGAGCGCGTGTTGGGTGCTAGCTGCCGCGATTAAATTGTATGCCCTAAGTATCCAGACAATGGAGAAAGGGGATAGTCGGTGGGAGGAAGAGGATGCGTTAGGGCTATAAA

>ERV_Env-Tac5_consensus

GCACGCTCACACACACACACAGGTGCCCTCTCTCCCTCCCTTTCTCTCTCTCTCTCGTTCTCTCTCTCTTTATTAACCATGTAATCGCTGATAGCAAATAAACGGCAACCAAGCTTTGGACCTCTGGCTGACTCTTCCTGGTGTGAACGCGCGGCCTGCGTCCGGAGACGTCCGAAGACCCGGAGAGGGTAAGAACCCGAGAGTTGCCCCCGAAACCCCGGGGAGCAACGACTGGCGCCCAACGTGGGGCTCGGGTACCCCCATAGGGGAAGACCAGGGAGAGTGCCCCGTAGGCCAGGTAGCTAAAGGTCAGGCGGGAAGATGGGGACAGTACGATCGCTGCCCATATATAAGCCAGAAGATAAGGAATTGTACACCCGGCACTGTTACAAGATATTGAAAAGTAAAGGGGTAAAGATTGAGCTGAAAACAATAAGGGAATTTATAGGGAAGGTAACTATGACCTCCCCGTGGATACTTGATTCCGGGATCACGGAGGAGAGATGGGACGTTATTGGGGAACAGATGACGGCCTATGAAGACTCCCACCCGGGGGAGCTAAAGGACGTGGACTTTCTTATACACGGTATCCTCCGTGCGGCCTTTCAAGGGCCAGAGAGACTCGTTCGTAAGTTTGACGCGACCTGCCAGACTGACAAGGAGGAAGCGGGGCAGGAAGGGGAGAACAGCCGACAACCTGAGGAGACTGGTCAGGCCGAGGCAGAGACACCCCCCCCACGGGCCCCGAGTTACCACCGCATCTATCCTGATCTCGGGCCTTACACCCAGAGTGAAATGCCCAACGCCGAGCGGGAAAGAGGAGGTCAGGCTACACAGACGGAGGTTGAGAATGAGATAGGGGATACGCGGGAACAGTTCAGGCAGATGGATGTGGGAAGCAAGAAACCGATCGTTAAAAGTGGGGAGGGGACGAGAGCGGTTTCAGCCCTACAGCGAGCTTTGGAAGAGGCGGTGGCGAGGGGAGAGGACGTCACGGGATGGGAGGTATTCCCCGTGATAGAAAGACCAGACGGGGGACGAGGTTTCGCCCCGATACCTTGGGCGAAACTCAAAGAGTTGAAGGCGGCGTGTGTCGCCTACGGTCCCAGCTCCCCTTATGTGAGCCAGCTCCTGGACACTATGTCCCTGGAAAGCGTTTTGACCCCGAATGATTGGAAATCCCTTGCCCGCGGGTGTTTGGATCCCGGACAGGGCCTTATATGGATGTCTGAGTTTACCACCGCTGCGAAAGAACTAATATGCAGACGGGGTTTCCCGAACCCCGCCGAGGCTTTTGCAGCTGTGACCGGGACGGGACGATTCGAGACTCCTGAGATGCAGGTCAACTACGAGCCCGAGACGTATATGGTGATTGCCAGGGTTGCCCTAACTGCCTGGCAAAAGGTTCCCGAGAAAGGGGACCATCGCTCCCCCTTAACGCAGATCAGACAGCGCCCGGACGAGGCGATTCAAGATTTCGTTTCACGCATGCAGTCCGCGGTCACCCGTATTATAGGGGATCGGGACGGCGCCGAAATTGTATTGAAGCAGATGATCAGAGAGAACGCAAATAGTGCCTGCAGGAAAGCATTGGCAGGGCTGCCCAGAGAGGCCACATTAGGAGACATCCTGCAAAGATGCGAAGGGGTTGGAGGAGAAGAGTACAAGGCGCAGATGCTTGCGGGGGCAATAATGAAAGGATTATCAGGAGTCGGGGAAAGGGGGCGACAGTGCTTTCGGTGCGGACGGATGGGACACCTGATGGCTCAATGCCGAGCTCAGGACAAAAGTGGCCCCCCAGCACAGCCGAGAAAGGGTGTGACTTGCTTTGAATGTGGGAAGCACGGGCATTATGCGAAACAATGCCGCTCGAGGCGGAGACCCCGAACGGCGTCGGGAAACGGGTGGAGGGGCCCCGCGCGGGCCCCGAATCACGCGTTCCCCGTGTCAGCCGCGGGAGAGAGCCCGAAAAGGGTTTCTCTCATAGAGGAGTACCAGAGGCTTCGGTCCCGGGAGCCACTTGGGCTGAAGTCCGATGCCTGGCCCCAGTGGAAATCCGACCCGGGGACACTGTAAACGTCCCAATACAGCCCCTCCCCTGGAGGGCCCTAGTGGTGGGGCTTCAGACAAGAGCGGCGGGGGTCGTTCATTCGTCCGAACGGGGGGAAGGGCCCGGGAAGATTCCCCTCACCAACCATCATCCGTATGGTATATACCTAGGATCGGGAATGGTCATTGCTCGCGCTACGCCCCTTGACCCCCCGCCCCCTGATCCCCCGCCCCCAGCTATTGGAATAATACAGGAAATAACCCTTGATAAACCGTGGCGGACCCTCTTAGTTGAGGGAAAGCCCCTTAAAGGGCTCCTGGACACCGGGGCCGATCGCTCCGTTATTCAGGATTGCTGCTGGGCAGCGGAGTGGCCACTAGCAAACCACTCGATGGGGGTGCAAGGCGTGGGGGGACTGCAAGCCGCAAGAGAGGCGGGCCGTTCGTTGATTTGGTCATGCCGGGGAAGGCAAGGTGCGTTCGTCCCGCTGTGCGTTCAAGGGCTCCGTATGAATCTGTGGGGTAGAGATGTGCTGCAGGGGCTGGGAGCCCACCTTATAGATGAAGCTGATCCTTTTTAGGTGGGGCCACTGGGTTCCGGGGCTTGTCNACACCCCCACTGGTATGGCTTCCACACCCACCGGTATGGGTGGACCAGTGGCCCCTGACCAAGGACAAGCTGCAGGCGCTGAGGGAACTAGTTGCGCTCCAATTTGCACAGGGGCACCTAGAGGAATCGTTCAGCCCCTGGAACGCCCCCGTATTCGTTATAAAAAAGAAAGCCGCGGGGAAGTGGCGCTTCCTCATGGATTTAAGAAAAATCAATGCGCTCATTGTGCCAATGGGACCCCTACAACCCGGATTGCCCTCCCCTAACATGATTCCAAAAAATCACCAGATCCGGGTCATAGACATTAAGGACTGCTTTTACAGCATCCCGTTACACCCTGACGACAGGGTGAAGTTTGCTTTTACTGTCCCGAGCCCGAATTTCGCGGAACCAGCACTCCGGTACCAGTGGAAGGTGCTGCCACAGGGCATGTCTTGTAGTCCCACCATATGCCAGTGGTTCGTGGGGCAGATACTCGCCCCTTTCCGCAAGGAATACCCGGGGGCGACGATCGTCCATTACATGGACGACATCCTTCTGGGTATGCCCGACCAAGGGCAGGTACAGACGCTCACCCGGCGAGTGGTGGCTGCCCTCGCAGCCCAGGGTTTATTCGTAGCACCNGAGAAGGTACAAGAATCAGCCCCGTACACGTACCTTGGGTTTGACGTCACCGAGACCCGGGTGACTCAAAGACCACCCCAAATTGACCCCCGAAAATATGTCACGTTAAACGATATGCAGGGTTTGGTAGGGAGGATTCAATGGATGCGCGCGAGAACGCCCATCCCCTCCGCCCTCATGCAACCCCTGTACGATCTCCTAAAAGGAGACCCCAATCTTAAATCGCACCGCGAATGGACGGAGTCCGCGAAAAGCGCACTCCGAGAAATCACACAGCGGTTGGCGGGTAGCCATACTTGTCGAGCTGAACCCCACCTCCCCATGGAGGTCACGATATTTCGGGAGGGTAGCCTTTTCGCGGCTATACACCAAGGAGCCGAGATTCTCGAATGGTGTTATCCTCGAAACCCCTCCCGTGTTCTCCCAAAGGAGACTGAGCTTCTCGGTCGCTTTTGCCAGAACGCCATACAGAGGGTAGTGGCGCTTTCAGCCACTTACCCTATAGTCCATGTCGGGATAGCCATGGGAGACCTTGAGGCGATAGCCAGGGACAGCTTCCTGTGGGCCATGCTCCTACAGCAGGCCACCTTTACGGAACGCTCCCCGTTGACTCTTAGCCACTTATACGAGGGATCGGACATTCTGACCCCTCGGGTGATCTCCGATACTCCGGTCGGAGGAGACAATGTCTTTACGGACGCCACCAAGGAACACCGGGCGGCAGTTTTCAATCAGACCACCGGTGCCTTGTCGGTGCTCGACACACCATACGGCTCGACTCAGCGAAATGAACTTTTCGCTATCATATGGGCAATGACTAACTACCCCCAGGCGATCAACATTATCTCGGATAGCCTGTATGCCGTTAATCTTGCCCGGCGAATCGAAACCTCCATACTTTTCGACAGGCACTCGGAGATTGGGAACATGATCTCTCAGCTTCAGGCTGCCGTAGCGGCTAGGGAATGTAAGGTATACCTCATGCACGTCCGTTCCCACACGGACGGACAAGGGCCGATCTTTGACGGCAACCGAACTGTGGATGCCAGCCTACACCCCACAGGGCCGTCACTAATGGGGCTGGATGCCGCGGCTGCGGCGCATAGGGAGTTCCATCTCCCGGCCACCTCGCTCCGCCGGCTGTATGGGGTCACTAGAGAGGAAGCCCGGTCTATTGTCCGGCGCTGTACTCGCTGTCTTCCGTTCACCCCACGGCCGGCAGGGTCGACGGGTGTGAACCCCCGGGGGCTCACGCCGAACGAGCTGTGGCAAATGGATGTCACCCATTGGGGGACCTCCACGATTCATATGACCATTGACACCTTCTCCGGATTCATGCTGGCGACACGCCAAGCAGGAGAAGCCGCAAAACATGTGCAAAACCATTTGTACCATTGCTTTGCCACTATAGGCACACCCCAGGAGATAAAGACGGATAATGGCCCCTGCTATGTCTCCAAAGCCATGTCCCTCTTTTTCTCCTCCTTTGGCATCTCCCATGTTACCGGGATACCTTACAACCCCAACGGTCAAGGTATAGTGGAAAGGGCAAACAGGACGCTGAAAACTCTCCTGCAGAAGCAGGGGGTGGGGAAGCGGGTCACGCAGTGCGCCCTAGACAAGGCAACGTACACCCATAACTTTCTATCTGTTGATCGGGAGACGGGACTCTCCCCCGCCATGCGGCAACTCTGCCACAGGTCCGCGACCGTGGAACCCCGGCGCCACCACCCGCACGCCCGCACTATGGGGCGAGCGATGTGGCGTACTGTGGAGGGCGATTGGCGCGGTCCTGATCCGGTACTAATTCGGGGTCGAGGATATGCTTGTATCTCCACAGGTGACGGCCCCGTTTGGATCTCCTCGCGACACCTCCGCCTCGTCGAGGAAGACGATGCCCAGCCGCAGGGGAAGTCCCAGCGCCCTGATTCCCCTCCTCCTCCCCCTCCTTCCCCTTCCCCTTCTTCTTCCCGGGGGCGCGGTGGGGAGGGAGGCCGGGACGCGGATTAACTGGACTGCTCTGGTAAATAGCCACCTGAAGGATGTTGAGGAGCAGAGCGGGGTTTGGAGGTGGGCCCTCCTCGCCAATGCGCCTTTGTTACGCATGGTCTCCTGGAGGGAGGACTCCCCCCAACTCTCATTTCAAGGGAACGTGACCAAGCTCACGGGCCAGCACCGGGGTCAGTCCCCAGACACTGACCAGCATGAGATCCGTATTCGGGGCGCTATCTTGCGCAGCACTGGTCCCCCCATATGCTGGTACACCGGCAATGGGGCTTTCTCGAACCAGCCCGAGGTGACCCGATCTTGCTTGCGGTTGCTAAATTTTACTTCTACACTGGAAGTCAACATACCCCAAGGTCAGGGGCACCCCACACACCGAAACCTTACCCTGCTGGGGATCCACGGCACACTTCAGTGCACCCCCGGCGGCACAAAATCCAGCGAGGGAAGCGGGGGGTATTTTGGCCCTATCCGAAATGCGAGCTCGCTCCCCGGTCCCGGGAGAATGTGCGCCGAGGGTCGGGGTGGGGTTTGGATGCCTCTAACTGACTGTTCCACGGGCATATTCCGCTGTATTCCCCATAGTGATAACGGTACCCTCCTGCTCTCCTGGGATGACCACATTGGGGACCGCACATATACGGAGGCGGCCGCCCCAGGGCTTTACGCGCTACCGTGGGGGACGCACCACCCCGGTCTCTGGAAAGCCTTTACTTCCCTGCGCTCCCCCACCGCGACCGTCAGGGTCCGCTCGTGCGCCTGCCCTGACGGGGCGACGTGCCAGGATTACCCTTTCTCGGTGTATCGTATTATTTTCCGTGAGGGTCTGAACGTTTCCCTGCACTTGCAGGGCAATTGTGCCGACCATGATTATGTCTTTCAAGGGAACGCCCTTATCCCTGCCCCTTACGCGCTTATAGCCTGCCCAAGTACTGATCCCAGCTGGAACCTTACTGCCTCCCGGTCAGCTTATGGCGACTTTTCCTTGTCCACCACGGGTTCCTCACGATGCGTCCTTATGTCTCGGGCGCGACCCATGACCACGGAGGGTGCGGCAGATTACGCTGTTTGGCTGGTCCGGCGGCCCCGCTACACCCTTATACCCGTTAAGTCCGCTGACCCTTGGTTTTCCCAGGCCTCCGACCATCGATGGCACACCCTAGCGACCCGCTTGCGCGGCTTGCGCACCCGCAGGGAATTATTTGAGGCCATGCTGGGTATCGCGAACCTCGCGTTTTCCGCCTTCCAGGAATGGCAAATCCTGAATCTGTACGATCGGACTGGTTTCCTCGCGTCTCAGCTCTCGGCCTTCATGCACTCCACCCAACTGGCGTGGGGGGTTCAGCGTCACCTGGACGCTTCCCTGCAGCAGGAGCTGATCGGCCTTGAGCACGTGATCGAGACGCTCGGCGACGAGGTTCGCCTCCTGTCCCTACGGCAGGCGGTGAAATGCGACTACCGGTATCGGCATGTCTGCGTGCTTCCGCTCCGCTGCAACGCCACCAACTCTTCGTTCCCTGCCTCCTGGGACGGAGTAAAGAAACACCTGGAGGGACTTTTCCTGGCCGCCAACGTGACCGGCGAGTTACAGGAACTGTGCCGCCTTATTGACGAGCTCAACGCCCAGTCGCTGCGCTTCCCCAAGGCTTGGGACGACCTAGGCCGCGGGGAGGACGCGGTGGAGGAACTTTGGGACTGGATTAAACGCGTGATGCCCGGGGGATTGGCCCCTTGGCTGGTCTCGGTCCTTGCCGCCACTGGGGGGGTTTTGCTTTTGCTCATGTGCCTCCCGTGCCTCCTTCGTCTCTTTTGCTCCATTGTTGCCCGGGTGGTCCGCGAGCTTAAGGTCGAGATGCTCCCATTGCGCGGACGCCCTTAGCGGTCCCCGCCATAACGGGAGGGGGACACGTGGGGAGCGTGGGAAAGATAGGGAGTATGTGCACACAAACCGTACCCAACGGCTCAAAGCCAAACAGACTCAACAACAGGAGATAAAGGGACCAGGGCAAGAAAAACAAGCTTGCACCTGCAAAGCTCGGAGGCAACCTCAAGGCCATATCCACAGCCAGCAATGGTTGGCAACGGGTGGCTGGGGGCCAGCCAGAGCAAACGCTTCGTGATGGACAGAGCTGTTGCTAGGCTAGTGGCTGGAGGGTGGCGAGTGAGTGTGCTACATGGCCTGAGAAAATCCTGCCCCCGGGCAACGGGGGGTATAAGAGACGAACAAGACAAGGGGGGGTGCAAGCACGCACGCTCACACACACACACAGGTGCCCTCTCTCCCTCCCTTTCTCTCTCTCTCTCTTTCTCTCTCTCTTTATTAACCATGTAATCGCTGATAGCAAATAAACGGCAACCAAGCTTTGGACCTCTGGCTGACTCTTCCTGGTGTGAACGCGCGGCCTGCGTCCGGAGACGTCCGAAGACCCGGAAAGGGTAAGAACCCGAGAGTTGCCCCCGAAACCCCGGGGA

>ERV_ENv-Tac6_consensus

GACCTAACATCTGGCCCATCAATTACTGGGCCTAAGTAGTAAGCCAGGCCTTCCGTGCTCACAGGAAGACCTACAAGGAACTGAGAGGAGAGCTGTGGACAGGCAAGAGTTACTTGTTATCGGCCCTACGGCCTTGCATAGACGGGTGTTGCTTGTTACTGGTTTTGAAGGTTACAGAATGGCTGTGTTAGTTATAAACACAGCAACATGGAAAAATACAGAATGTGACTGAAAGATATAAAAGTCTTGCTGCTTAACCCAATAAACAGTCTCCTTGTCCACCTTGAGACTCGCCTGGCCTGCGTTCTTCCATTGCGAGTGCCCGTCTCCCCACTGCGCTGGACGCGTGGAGGCAGACGGCAACAAATGGCGCCCGAACAGGGACAGGGACATGAAGCCTGGACCCTCAGACTCAGCACCGCAACCCTCACGAAGCAACTCATCACTGCGCAGTGACTCACTAAGGAACCGAAGAGGTGAGGGCATTTAAGTTTACTTTCATTTTCAAACACCAGGCTTACAGCAGGGAAGAAAATGGGACAAATTCAATTCCGTTCTCGGGATCATTATATCCAATTACTTAAGGATGTTGTAAAGGAGCGTGGGTTGATTATCACCACTGCCCAGATTGAACAATTTTTGGAATGCGTGGAAAAATGCTGCCCTTGGTTTCCAAAGGAAGGAAAGTGGAATCCTTTCACTGAGCCAGCTCCTTCCGCACCACCNCCTCCACCACCCCCTCCATGGGTCCCGAAACCAGCACAAACACCTCTGCAGCGAGCGATGGGGGCTGCTATGGAAGGGGGAGAGGAGATACCTATTGAATTATTGATGGGTTTCCCTGTGATGGAGCAACCTGACCCTGCTAATCCTGGTCAGTTAATACGTACCCACGAACTCTTGAATTTCAAACTATTAAAAGAAGTGAAACAGGGTGCTGCGACATATGGGCCCACCGCTCCTTATGTGCTGGCACTCATAGAAAGTATTGCTTCCTCAATCCTTTGCCCGTATGATTGGCAAACCTTAGCCAAGACTGTCCTGGATGGCGGAGACTATTTGCTTTGGAAGGCAGAATTTTTTGATTTGTGCGCGGAGCAAGCGCGTCGCAATGATCGCGCCCGACCTCCAGTTCAAATCACTTTCGAGATGCTTACAGGGGCTTACAGGGAGCTACAGACTCGTGAAAGCTTGTCCCAGCCCCTACCCAAAGGGGTACTGATTACATGGAAAACTGTTTTTACGGCGCTGCGGGCGCTGCATCCCGCTGAACGGGTGTTACAGGAGGCGCCGGCGGCGCGCGACTTGGGGGCAGGGAGTGATCAGGAGGACCCCCTGTCCTTTGAAGTTTTGNCNCCAACAGAAGAATTGGAGCCGGTGTACGCTACTGTTCCGGATCCCGAGGACTTGTATCGGGACCAGGATCATAATGACTCTGGCGATCCTTCTGACTCTGGGACTGCAGACCCGAAGGAGGGATATGATCTATACCCGCCTTTGGCCCCTCTTACAACGACGGCTGCTGTCGCACCCACAGCAAAGGACCTTTTGCAGACCCAGNGCCTGCAGGCGGTATCCCGTTCGTCGATCGTTCCCCCTCCCCCCCTTGCTCCCCCTTTGTCTGCTGCGCCGCCGTATGTATATGCTGTGCCTGCTACGGCGCAGCCTGCCTATACCGGGGCCCTTTCGGGCTGCCGGCAGAAGGCATTGCGCCGAGGGGATGTACAGCTACTGCAAGCGATGCCGGTGGNGTATCTTCCCACCGGCCGGCCGGCTCAGTATGACTCTCTCCCTTATGAGTTGATTAAGGAGTTGCGGAAAAGTGTTCGCGATTATGGGTTACAGTCGTCCTATACAATGAATCTAATGGGGTTGATTGGGTGGATGAAGACTATNAANTAGCTGTTAGTGGCCGCTACCAACCCCGAAATTGGCTGCTTTACAAGTGATAGTCGCGGAACAATTAGCGGCGGGGCATATTGAACCCTCCGATAGCCCTTGGAATTCCCCTGTCTTTGTGATAAAAAAGCGCTCGGGGGCCTGGAGGATGTTGACTGATCTCAGAGAAATCAATAAGACGATGCAGCCGATGGGAGCGCTGCAACCTGGGCTCCCGAACCCGGCTATGATCCCTAGGAATTGGCCGATATTAGTAGTAGATATTCAGGATTGTTTTTTCTCTATTCCGCTCCACCCGGACGATCGTATTCGATTTGCGTTTTCAGTCCCTGTGGCTAACAGGACTCAACCTACTACGCGATATCATTGGACGGTCCTCCCCCAGGGAATGAAAAATAGCCCCACAATGTGCCAGACTTACGTTGCGCTTCTGATAGCGCCGCTCCGCGTCAAGCATCCTGAGGCATATATTATTCACTATATGGATGACATATTATGTGCTTGCGCCACCGCAGCCCAACTACACGCCTTACGGCCTGCTCTTCTGGCGGCTCTAGAATCTGGAGGGCTGCGGGTTGCACCCGAGAAAGTCCAGACAAAGCCCCCGATCACCTATCTGGGACATATTTTGACAGATCGGACAGTGTCCCCTGTGACCCCTACCTTGGATGTTTCAAAATTAAAAACCCTTAATGATTTCCAAAAATTACTGGGGGAATTAAACTGGGTGCGCCCTTATCTTGGAATCCCCACCGATCAATTATCTCACCTTTTTGCTACCTTGCGAGGAGCCCCGGAGCTCTCTTCCCCCCGCTCCCTTTCACCACAAGCCGCCCGGGAGTTAGAGCAGGTAGTCAAAAAATTGGCTCAGGCTACGGTGGACCGTGTCACCCCAGGGATACCCTTATCCGCCGTTGTCGTTCCCACCTCTGTCATGCCTACAGGGGTTATATGTCAAGAACAACAATTGGTTGAGTGGATGCATTTGCCCACTCAGCCCCACCGCTCTGTTTCCCCATATCCGACTTTGGTAGCCCAGTTATTGGGTCGTTTAATTCGGCGCATTACCGTCCTTTCTGGGACCGGACCCTCTGCATTATACCAACCATATACTTCAGATCAACTTGCCACACTTCTTGGCAACGATCCCGATTGGCAGATCCTTTTGGCAATGCATACCGGGGCTTGGGTTACTGGTCTCCCAGATATTCCACGATTAAAGGTTTTCTCACCTTTTGCATGGGTATTCCCCTGCATCACGGTTCCGGACCCAATACCGGGGGCTACCACAGTTTTTACAGATGGAACGAAAGGCCGAGCCGCCTATTATTGTCCTCCGGACACTTCCCGCGTTATCCCCATTCTGTCCCATTCCGCTCAACACGCGGAACTTGTAGCTGTGATGTCAGTCCTTCGCGATTTCCCTCAATCACTTAATATTATCTCAGATAGCCGGTATGCATGCTTTGTTACTCTTCACATTGAGACTGCTGCGCTCCGGTTTTCCGAGGCAGCCATTTTTCCTTTGTTCCAGGAACTTCAAGCAACGATTCGCGCTCGTTCTCATCGTTTTTATATTTCTCATATTAGGGCGCATTCGTCCCTCCCCGGACCATTGGTTGAAGGTAATGCTTGTGCGGACCTACTTACTAGGGAAATTGCTGGCGCACCAAAGGCGATTAATTTTGTTGCACCACTGTTGGCTCCGGCTCTTCTCCCTTTAGATGCTACAGCCGCAGCCTCGCAAGCCCACCGGCGCTTCCACCAAGGGGCAGGAATGTTGCGCCGTCAATTTGGCATTTCTCGGGAAAGCGCGCGGGCTATTGTGAAAGCTTGTACCTCCTGTGTTACTACATTACCTGCGGCCTCTGAAGCCACGAATCCCCGTGGTCTTGCGCCAAGAAAGCTGTGGCAAATGGACGTCACGCATTACCCTCCCTTTGGCAGATTGGCTTTTATTCATGTTACTGTTGATACCTGTACATTATTTACTTTTGCGTCGGCTCACACTGGTGAATCAGCCAAACATGTTATGGATCATTTGTTTCTTGCTTTTGCCTTTATGGGAATTCCACAGGTATTGAAAACCGACAATGGCCCCGCATACACTTCTAAGGCGTTCCGGTCTTTCTGTAATACTTTTGCTATCCGCCTTGTTACGGGTATTCCATACAATCCCACGGGTCAAGCCATTGTCGAAAATCGTCATCGTTGGCTCAAAGCCTTTTTGGAAAAACAAAAAGGGGGAGATGAACTGACCTCTCCCCATAGACAATTACAATCCGCTATATATACCCTTAATTTTTTAACTATGGATGACAAAGGTCTCACCCCCGCTGAAAAATGGGGTGGTAGGGAAACTAAAGAACACCCAGAAAGAGTAAAATGGAAGGACCCCCTCACGCGACAGTGGCGAGGGCCAGATCCCTTATTAACGTGGGGCCGAGGTTATGCTTGTGTCTTTCCAGAAACTGAGGACCGCCCGATCTGGATACCTACAAAGAACATACGCCGCGTCCTTTCTACAGTGCGTTCTCCCTCACCCTGCGTACTTCAGGAGCTGACAGAGAACCAGGATACAGACTCCGCGACCCTTCCTCTGGAGGATCAGCAGCAAAGTTCCTAAAAATGATGATCGCTTTCCTGATTCTCGCCTCCTCCACTGTTGTGATTACTGAGGACTATTGGACTTTTGTTCCGTACCCTCCGTGGGTGCGGCCCGTTACGTGGCAGGACCCGGTGGGAGATATTCTCACTAATGCTTCTGATCTTGTAGGGGGGGATGCGATTCCATGGGAAGGACCAGGGGAAGAGGCACTTGTCAATGTCTCCCTCCTTGCTACTCGCACTCCTATATGTTTCTCTGCAGCTCCGACCCCAAGTCCGTGTCTGTCTCTGCTCCCCCTCACTACTTGGGCTCAAGAAAGAGAATTGCGTGAACCCCAGCCCCGTTCGGTGTACTGTGGGATTACCCGTTTGACTGCTGTATCCCATCGTTTGGGAACCTCACCTTCGGTACCCGAACTTCCTTCTTGTGTGTTTGCCAGCTCGTCTTGGCCACCATCAGAGTCTGTTTTGCCTATTTGGCAAACGTGCCGACAACCTTTTCCCACGGTTATTCATTTGTCCTCTCACGTATTCCCAGCCACCTTACTGAATTGGGGCGGACATGACAACCTTACTTATTTATCCCCTTTGCATGGTTATCTTGGGTTTCTGCCCACGGATGCTTTCCAGGCTTTAGGTTCCTCTCGTGCCGGAACTTCCTCCCCTTTGTACCCACACCTTTGGCGGGCTTTTGCTGCTTTGGGTCCAATCACACTTGCTTTTGCTCGATTACAGGTCCCTTTCGATTCTATCCCAGTCCATATGGAAGAACGGCAAATAAGAGCATGTATATTGCCACCGCAAGCCTTCCTTGTTGGCTACCCGTCCCTTATCCCCTTCCCTTCCCTCTGGAATATTACATGTGATAATTGTAGACTTACCCAATGTATATCCGCCCATGATGCCACTGCTACTATTCTTATAGTAGAACAACCTCGTCATGCTATACTACCCACCTCATTATCCCACCCTTGGGCTGAGTCCCCTGCGGATTCTGCCCTGCAGATGCTAGCTGATCATTTTCGTATTCCAAAACGGCGACATCGTCGGTTTGTTGGATTGTTAATCACTGGGTTTATCGCTTTGGCATCCCTTATTATTGCTACCACCTTATCCTCTATTACCCTGCATACTGAAATAACACAGGCCTCCTTTGTTAATTCCCTTGCAAGAAATGTTTCTGATGCTCTGCAAACACAGTTACGATTAGATAAAGCCTTTACCGCTCGCCTCTCTCTTCTACAGCATTCTCTTATAGAATTAGGAAATGAGGTTGATGCTCTGCGCACTCAACTTTCTTTTCAACGTTGTGATTATCGTTACCCTCATATCTGTGTGACCCCATATTATTATAATGCCACATTTCCCGGGTTTTCGTGGTCCGCGATCCGAAATCGGCTCCAAGGAGCCTGGCATTCTTCGAATGTCTCCCTTGATTTGTCACACCTTGCCTTACTCTCCGATGCTATGCAAAATACCCCTCCCTTGACCATTCCTGCCGATATTGCTTCACCGATCATTTCTGCCCTTGAGGCGGTTAATCCTTTTAATTGGCTCTCAATGCTGCTTCATAGTCTCCCATATATTATTTTGTTGATCTTGGCCCTGTTTTGCCTGCCCTGCCTTGCCCGATGGCTATCTGTCTTCCTTCGCCCGCTTCTTACTCAACTCCATGCTTTGCAATTGTTTCAAAAACAAAAAGGGGGACTTGTGGCAGACCTAACATCTGGCCCATCAATTACTGGGCCTAAGTAGTAAGCCAGGCCTTCCGTGCTCACAGGAAGACCTACAAGGAACTGAGAGGAGAGCTGTGGACAGGCAAGAGTTACTTGTTATCGGCCCTACGGCCTTGCATAGACGGGTGTTGCTTGTTACTGGTTTTGAAGGTTACAGAATGGCTGTGTTAGTTATAAACACAGCAACATGGAAAAATACAGAATGTGACTGAAAGATATAAAAGTCTTGCTGCTTAACCCAATAAACAGTCTCCTTGTCCACCTTGAGACTCGCCTGGCCTGCGTTCTTCCATTGCGAGTGCCCGTCTCCCCACTGCGCTGGACGCGTGGAGGCAGAC

>ERV_Env-Tac7.1

GGATCAGTGGAAAAGAGCATGGGCTTTGGAGTCAGAGGTCATGGGTTCAAATCCCTGCTCTGCCAATTGTCAGCTGTGTGACTTTGGGCAAGTCACTTAAATTCTCTGTGCCTCAGTTACCTCATCTGTAAAATGAGGGTTAAGACTGTGAGCCCCCCGTGGGACAACCCGATCACCTTGTAACCTCTCCGGCGCTTAGAACAGTGCTTTGCACATAGTAAGTGCTTAATAAATGCCATTATTATTATTATCATTATTATTATATGACTATCCCCTAAGGCTTACAACATCCAATCCTTCCATCCTCCTATGGCATCAGGGACACTGGGGCCTCTGTTGGACATTAGACAGGCATCCCCACAAAGTTGTTGAAGGTAGAGCCAGCTGAACACTCCACTGTCCACTAGTGGACAACCAAGCCAGGGCTGGTTTCTGCACGGAGCCCCAACCCAAAAGCCTTGTATAGCAAGGGGCTGGGGGATGTACCAGGCCATGGAAACTGGGATAGGGACAAGGTAAGTAAGTGAACATACTTGTCCTCAACTTCTCCCTGCCCCTTCCTCCTCAATCCTAATTCCCTTCTTGGACATAACATCTCCTCCAAGAGGCCTTCCCCAATTAAGCCCTTTTTTCCCCTGGCTCCCTCTCCATTCTCCCTTGTCTAGGCACTTGAATCTATGACCTTTGGGCAATTTGATATTCACTCCACCTCCAACTCCTCAATACCTTGTACATTTCTTTAAATTATATATTATGAATTATCTATTTATAATATTATCTGTCTGCCCCTCTTGACTGTAAGTTTGCTATGGGTGGGGAACATTAACCACTAATTCTGCTGTATTGGACTCTCCCAAGGGCTTAATACAGTGCTCTGCACATAGTAAGTGCTTAATAAATACCACTGACCTGTTTACTGATCCCAGAGTTTGAACTTTCTCCTTCCTAGTGCGCTTCTCAAGAGTTCTGCTCAAGGGACTATGTGTTTTCCTGGGGTTTGGGGAAGTGAATATATTAGTCTGTTAGAGAAAGAGGTATGAGAATTTCCGTGCCGTGACCCATCCATTCCCACATCTTCTCTGCTACTACCACCAGTCCCCATAGGAGTGTTCGAAAGTTTCAGAGGGTGCAGACTAGTTGGGGTGAGAGCAGGGTCCGAAGGTGGAGCCAGACAGGGTAGGAACACTGTCATAAAGACTTATCAATAGGAGAAGCAGCGTGGCTTTGTGGAAAGAGCACGGGTTTGGGAGTCAGAGGATGTGGATTCTAGTCTCTGCTCTGCCACTTGTCTGCTGTGTGTCCTTGGGCGAGCTGATTAACTTCTCAGTGCCTCAGTTACCTCATCTGTAAAATGGGGATTGAGACTGTGAGCCCCACATGGGACAGCCTGATTACCTTGTATCTAGTCCAGTGCTTAGAACAGTGCGTGGCACAGAGTAAGCGCTTAACAAATACCATAACAATGATAATAGTAGCAATGGTTCCTGGCTAGGAGATCTCCACCAAGGGGCAAAGAGTCGCACTAGGACTGAAGGATGGGTGGTCGATTCAACCTGCTCAACCTGTTTGTGACCAGGCTGCAGGAGCCCAGGATGCTGGGCATCTTAATTGCGTGTGAATCATTGGTTCAACGTGGTGAGGGCCAGGCCTACCTGTGATTACTGCTTCCCTGGTGCACCTCCATCTGTCCTAGGATGCATGTGGAGACCACAGCAGTCGGTGTGGGTACTTGTTTTGAAGACAGTAGGTAGAGACAGTGAAATTGAATGAAGACCTGGCTCTCCTGACAGTAGGGCTCCCTCTGCAAGACTGCAAAGGCAGCAGCCGAGAGGGCCAAGGCAGCCGTTTCGGGGAGTGGAAACTCATCGGCACCCTTTCACTGACCACAATGGTAACAATATTGCAGCGTGGTTGCCAATCAGCACCCTGCAATATCCTGAAATCCAAGAGTGGCCTGCTGACTATCTCTGTAGCTGGAAAATGCTGTTTGAATGGGGTGGAAAATCCACAGTCCCTGAAAGACTTAATGAACCATTGGAGGACTGGGAAATGTTGAAGGTGGCAGTGCCAACAGGAACTATTGGGCTGGTTGAGGTATAAATCTTAGGATCTGCTAGACATAGGTCTTCGGGGGAAAATAATTAAGGTTGAGCAGAAAGCCTGAGGTAGGGAAGGTGATGGTAGCAGGAAGAGAGGATTATCTTCAGGGTAAAACAGATTGGGAAGCAAGAACAGGGAAGGTGATTAATCAGTCAAGCAAACAATCTGTGTACAAAGTATTGTACTAAGCTTTGGGGAAAGCACAATTATAATTGTACTTTCCTGCCCATAAGGAGGAGGTTACAGTCTCCAAGAGGAGACAGATATTCAAATAAAATACAGATAGGGGACATAGTTAAGTATCAAGATATTTACGTAAGTTCCATAGGACTCTTATGAGTATCAAAGAGCTTAAAATGTACACAGAAAAGTACATAGTTGATGGTGAGAAGAGTGGAGATAAGCAATATAAAGGCTTAGTCAGGGAAGGCCTCTAGGAAGAGATGTGATTATAGGAGGATGCCTCTAGGAAGAGATGTGATTATAGGAGGATTTTGAAGGTGGAGAGAGTTGTGTTCAGTGATATATGAGTAATAACAATAAAAATTATTGTATGTGTTAAGCGCTTACTATGTGCCAGGTACTGTACTTGGGGCTGGGGTGGATACAAGCAAATCGGGTTGGACATAGTCCCTGTTCCATGAGGGGCTCAGTCTCAATTCCCGTTTCACAGATGAGGTAGACTGAGGTACACTGTGAGCCCATTGTTGGGTAGGGACCGTATCTATATGTTCCCAACGTGTACTCCCCAAGCGCTTGGTACAGTTCTCTGCACACAGTAAGCGCTCAATAAATACGATTGAATGAATGATGAAAGGTAAATGAAGCACGGAGAAGTTAGTGTCTTGCTCAGGTCATACAGCAGACAAGTGGCGGAGCCAGGATTAGAACCCGTAACCTTTTGATTCCCAGACCCGTGCTCTATCCACTACACCATGCTGAGTTCCAGGCCCAAGGGAGGATATGGGCAAGGGGTTGGCGACAAGATAGATGAGATCAAAGTACATAGAGTAGGCTGGCAATAGAGGAGCTGAGTGTGCATATTGGGTCATAGTAGAAGATTAGAAGGGTGAAATACAATGGAGTGAGCTGATAGAGAAATAGCGTGGCTTAGCAGAAAGAGCACGGGCTTGGGAGTCAGAGGTCATGGGTTCTAATCACGGCTCTGCAACTTGTCAGCTGTGTGACTTTGGGCAAGTTGATGATGATGATGAAGTCACTTAACTTCTCTGGGCTTCGGTTACCTCATCTGTAAAATGGGAATTAAGACTGTGAGCCCGTCGTGGGACAACCTGATTATCTTGTATTTACCCCACTGCTTAGAACAGTGCTTGGCACAGAGTAAGTGCTTAACCATGAAGGCGCTGAAACAAGTCCCTGATGCGGGTCGGCGGGAAACATCTTTTGCTGCCGTTCGTCAGGGTGCTCAGGAACATTATGTTACTTTTCTGGATAGGCTCCAAATTGCCATACAGCGTCAGATTGATTAGGGGGAGGCTCGCGGTTATTATTATGTCAATTGGCAATTGAAAATGCCAATGTTGATTGCAGGAAGGCATTGGATCCCCTACGGAACAAGGCAAAGACGATATCAGACCTAATTAAGGCGTGCCAGAATGTTGGCTCTGAGCACTTTAAAGCTGAAATGTTAGCCTCTGCCCTGGCGCAACAACTTACGGTGGCCCGGGCGGCGGTCAAGTGTTTTTCATTTGGACAGGAAGGGCATATAAGGCGTGACTGTCCTAAGAGGTTTAGAACCCGGCAGGTAAGGCGGGACGTTGTGTCCTTACAGCCATGTTCCAGATGTCACAAAGGATTCCACTGGGGTAGTCAGTGCAAATCCAAATTTGATGTGCAGGGCAACCCAGTGTGGCAGCCGGGAGATGGCTGGAGGGGCGTGAAGCCCGGCGCCCCGAGGACGGCACACAGGGCTACGAGAAATTTCGTGGGTCAGGCGGGAGCTCCGCAGTCGGAGGGTCTCCGCGACATGTCTCTGACCTCCGCCCAGCCACCGCAGTGAGCGCCGGGTTGGACCTGGCCACTGCCAGACCAACCGTGATTTCCAATATTAGTGTACATTTGCTGCCGACGGGCGTATTTGGACCCATGCCTCTGAACACCATGGCCCTTTTGATAGGTCGATCTTCAACTAGTAGAAGTGGGCTGTTTGTCTTACCCGGGGTTATTGACCCTGATTATACGGGGGAAATCAGGATTATGGTCTGGACACCGACACTGCCTTGTACGGTTCCCCCGGGTGAACGCACTGTTCAACTTGTTTTGCTCCCAAGTAGGGGAACGGGTGGTAGTGTTCAGACACGGGCTGGAGGTTTTGGCAGTACCGGTCCACCACGGATATTTTGGACGCACAAGGTCTCCCCAGGACAGCTCTTGCTGACCTGTTTGGTCAATAATCGCCCTTTCACTGGCCTGGTTGATACGGGTACCGATGTTACTATCATTCAGCAGTCCCAGTGGCCTCCCGATTGGCTCCTAGTTTGCTCAGCGGCTGCTGTGGCGGGGTCGGTGGGATGCAAGTGTCATGGCAGAGCGCTCATTCCTTGCTGGTTAAAGGGCCAGAGGGCAAACAGGGCGTATTACAACCTTATGTCCTGGCAGTGCCTTGCACCTTGTGGGGACATGACTTATTAGTGCAATGGAACGTGACACTTATGACAAATTTATAGCGGGGGCCACTGCAGTTCAACCTCGCCTTAAACTGACCTGGAAGACTGACAGGCCGGTTTGGGTAGATCAGTGGCCCTTGAAGGGCGAGAGGTTGGTTAAGGCATGATAACTTGTACAGGAACAACTGGCCTTGGGGCATATAGTTCCTTCTACCAGTCCCTGGAACACCCCCATTTTTGTCATACCCAAAAAATCGGGAAAGTGGCGACTACTGCAAGATTTGAGGGCGATTAATGCAGTAATGACACAAATGGGGCCCTTGCAGCCAGGCACGCCTTCCCCGTCCATGCTTGCTGAATCCTGGCATTTAAGGGTGATTGACTTGAAGGACTGTTTTTTCACGATCCCACTTCATCCGGATGATTGTTCGCATTTCGCCTTCTCGGTGCCCACAATCAATAATCAAGGACCACTGGACAGATACCATTGGGTAGTATTACCGCAAGGCATGATGAATAGCCCTACTATTTGTCAGATGGTGGTAGGCTCGGCGCTGGATCGGGTACGGGCAGTCCACCAGCATGCTATTACTTACCATTATATGGATGATATTCTTATTGCAACACAAGATTCGCGAGCCCTCTCACTCGTGTGTCAGAATGCCAACCAGCAACTACAGCACCAAGGGCTGCCGATAGCAGAGGAAAAGGTTCAGACTGTTGCACCCTGGAAATACTTGGGATGGAGACTGTATGAATCTGAAATCCATCTACAGCCCCTGCAAATCGCTAATACCATAAATACCTTGAACGATTTACAGAAACTCTTAGGTACCATTAACTGGCTTCGGCCGATTTTGGGCATAACGATGGAGGAATTATCTCCGCTTTTTCACCTGCTACGAGGTGATCCTGACTTGACATCTTCGCGTACTCTCACCTCGGCAGCATCTACCGCGTTACAGCGCATTGCCACAAGAATACAGACTAGTTATGGTCACCGACGCTCGAACTCCCTCCCAATTCATTTATTGGTGATTTACCACACCTTCCAGCTGTATGCGGTGCTGGGACAATGGACAGAAGGAGCCGTCTCTCCTACTGTGACACTGCGGACCTTGGAGTGGCTTTTTCTCCCTCATTCCTTCTCCAAAACTGTGACAACCCCCATTGAGATGATGGCCCGTCTTATTTCTCACGGGCGCATCAGATGTCAACAATTAATGGGGGATGATCCCTCTTTTATCCACATATCGGTTACTCGTGCCGACCTTGACACCTGGCTATCACAAAGCCTTGTCCTGCAAATTGCACTTGTGGACTTTCTAGGAACGATCGAATACACCCTGCCTAGACACAAATTGTTGCAGTCGTTGCCCCAGGTGCCCTTGCAGCCGCGGATCCTACTTTCACATGTTCCGTTGCCTAAGGCATGCACAGCCTTTGTAGATGGGTCCGGAAAGACAGGAAAAGCGGTAGTCGTCTGGAAAGGTGTCTCAGACGCTTGGGAATCAGACGTTTTCCAAGTAGAGGATCAACACAGATTGTGGAATTGGTCGCCGTTGTACCCGCCTTTGAATTATTTACATCCGAAGCTCTGAATCTGATGGTAGACTTTGCGTACGTCTCGGGTGTAGTCCAATGCCTGGAAGGGGCCTTTATTAAGGAAGTGGAGAATCGCCTCTTATTCCAGTTGCTTTTGCGATTATCACGCCTGTTAACACAAAGGTCTCACCCATATTTTGTCGTCCATATTGGATCACATACGGCATTGCCTGGTCCGCTGACCGAGGGCAATGCTGTAGCGGATGCTTTAAAAATGCATGTAGTACGGCCAAATCTGTTAACACAGGCCCGGCTTTCACACGACTTTTTCCACCAGAATGCACGGTCTCTGCACAAACAATTTTTGCTGACTATTCAACAAGCACGGGATATTGTTCGCGCTTGTCCTGATTGTCAGCAACTGTCCTCAGTACCTATTCCTGCAGGGGTAAATCCCCGCGGCCTTCGATCCAATGACATTTGGCAATCCGATGTTACTCATGTGGCCGAATTTGGTAGAATGCATTATGTTCATGTTACTGTTGATTCCTTCTCGCATCTTGTTGTGTCCACCGCCCATGCAGGTGAAAAGGCTTGTGATGTGGTTTGACATTGGTTGCACTCTTTCGCCGTCATGGGGGTCCCGGTTACTGTTAAAACTGATAATGGCCCCACCTATGTCTCTCGTAGGGTACAACTCTTTCTCCAAGATTGGGGGGTGCACTATATCACTGGTATCCCTTACTCCCCTACTGGGCAAACCATTGTGGAACGGATGCACCACACGCTTAAAGCTTTGTTGTTTAAACAAAAGAGGGGGAATCCCACGGGAATGACTCCCCAGGAACGGTTATACAACGCTACATACGTTTTAAATTTTTTAACCTTGTCTGATTTGTCCCTTACAGCAGCACAGCACCACTTTGGACGAGACACTACGTGGCCCGAACGGCCCCGTGTGTACTATAGACCTCTTGGGTCGGAACAGTGACAAGGACCTGCACCTCTGATCACCTGGGACCGTAGGTACGCCTGTGTTTCTCTGCCTTCTGCCCCCTACTGGCTGCCGGCGCGTTGTGTCAACCCCTTCAAGACGAGATACCACAGTCTGCGGAGGGCCACCAGGATGGCGAAGACGCGGGCGGCGCCCCCTGATACAGGGCTCAACACGGTCAGAATGTTTATGATCTGGAGCCTGATGGGAAGTCTTTGCCTGCTTGTCACGGGGAATGGACTTTACTGGGCACATATTCTTGATCCACCCATCTTCAAACCTATAACCTGGTGGGATCCTACACCACCTATCGGGAATAATGACACTGAATGGATAGGTGGGCTATGGATGCCCACGACGATAGACGGAGGGTCTAATAATACTAGTTTTTTTGCTATCAATAATACTGAGTTTGTAACTGACCTTCCACCCCTTTGTCTGACAACTAGTAATGACAGCGACATCGGCTGTATCCTGCTGTCGCCTCAGCAACACCTGATAGTGAGGGACAAACGTGGAACAAAGAATTCCACTTTAATAAGCCTCCCGGGTGTGTCTGGAGCATCGTCTCTCCACAACCTGTTGGAAGTGGGGATATATACCCCCGACTTGCCCCGTTGTACGGAGGACCCACTCACAAACAACACATGGATAAACTGGCAGCCATGTCACGGGCTCGGTCCTGATCCTGTTACTGTCAACGGAACTACGGGAACCATTTTGGACTGGGGCCCTCATGGGGTTTTGTGGGACTCGGCAAGCAACCACTCTGTTGGGTTCGGCATCCATAACCATAGTGTATCCTGGCATGGTGGTGGGTTAGCTGGGCCTCTTTTACAGTTTTATATGCCCGCCAACAACACTGCCTCCCCATTACATATGGGTATTTGGCATTTAGGCTTCGCATTTATTAATCGTACCTTATGGAATCTCACCCTGTCTAATCATACTGCTAACGCTACACAGTTACAAACTGATCCAATTATGATTTGCACTTCACATCCCTACATCTTTGCAATGGCACAAGTGGCTACCATAAGCCACTGCAACCATACTTACTGTACTAACCTGACCAATGTATGGCATGGAAATTGTTTCTCTTCTTTAAATGCACTACGCAACAATCTTACCATTATATTTCCCCTGTGTCAGTGCCCGGAGCTGTGGCTTCCTGTTAACCTTACACGTTCATGGGAAGGTGAGAGCGGTCTAGGTCATTTTGCGCGAATAATCCAGGAAGAGGTGGATAGCTCTAATAAGCGCCGAAAACGATTCATAGGGTGGTTGATATTTGCACTTGTATCTGCTATTGTCATTCTGGCTTCTGCGACCACTGCCATCGCCTCACTGGCACAGTCAGTGCAGACGGCAAAGGTGGTAGAGGAAACACTAACCAACGTCACGCAGGAATTTGAGATGCAGGAACATATTGACGAGGAAATCATGGTGAGATTACAGGCCCTCGAGGCAGCGGTAATCTGGCTCGGAGATCGACAGACAGCCTTAAAGACCCGCCTTTCGTTGCATTGTGACTGGGAGCATATCTCTGGAGGCCTTTGTGTGACCCCCTTGCCCTGGAATGCTACCACGCATCCATGGGATACGGTCAAAGAACATCTGCAAGGCGCGTTTGACATGTCCTTACAGCACGATGTCCGATTCTTGCATGATCAATTAAAAGGCCAGATTCAGCAGCTGCAGGCATTAAACACTCAGAATGTTCTCGAAACCCTTCAACGGGATATGTCTTGGTTGAACCGAAAACGTGGTTCACGGGGTTAAATCTGCACGTCTGGGTATTTGTCGGTATAGCAGGAGCCTTGCTTTTCTTCCTTTTGCTCTTTTCCTATATGACATGCTTGCTTATTAGGGCCACACGCACGGTTGAAGCTCGTGTGATGGCCACGTTGCTCATTAATGGTATAGTTATAAATAAAGGAGGGGGAGATGTGGGGAACAGCAGGGCCAAGTAAAGAAGGAACTGAGAGGAGAGCTGTGGCCAAAAGGCTGGTCTCTGTTATTGCAGCCTTGTACAGGCAAGAGTTACTTGTTATCGGCCCAATGGCCTTGCATAGATGGGTGTTGCTTGTTGCTGGCTTTGAAGGTTATGGAATTGCTGTGTTAGTTATGAACACAGGAACATGGAAAAATACAGAATGTGACTGAAAGATATAAAAGTCTTGCTGCTTAACCCAATAAACGATTTCGTGCTTACCCTAGCCGGAGTCCGTGCCTTCATACACCACACTTAACAAATACCAAGATCATTATTATTGAGTGCCTTCAAGCCTATTTTAGGGAATTTTTATTTGATGTAGCAGTAGGTAGGCAATCATTGGAGGTTTTGAGTAGTGAGGAGATGTGGACTGACCATTTTTTCAGAAAAATGATACGGGCAGCAGAATGAAGTATGGACTGGAGAAGAAGAGATAGGAGACAGAGAGATCAGCGAGTTGGCAGATTCAGTAGTCAAGGCAGAGAAACAAAACAGTCTAGTCGATAGGGCATGGGCCCGGGAGTCAGAAGGACCTGAGTTATAATCCCAGCCCTGCCATTGGTCTGCTTTGTGACCTTGGACAAGTCATTTAACTTCTTTTCAGCGTGGCTCAGTGGAAAGAGCACGGGCTTTGGAGTCAGAGGTCAGGAGTTCAAATCCCGGCTCCACCAATTGTCAGCTGTGTGACTTTGGGCAAGTCACTTAACTTCTCTGTGCCTCAGTTACCTCATCTGTAAAATGGGGATTAAGACTGAGCACCCCTTGGGACAACCTGATTACCTTGTAACCTCCCCAGCGCTTAGAACAGTGCGTTGCACATAGTAAGTGCTTAATAAATGCCATCATCATCATCATCCTTCTTTGG

>ERV_Env-Tac7.2

AGTGTGGGGAACAGCCGGGCCAAGTAAACAAGGAACTGAGAGGAGAGCTGTGGCCAAAGGGCTGGTCTCTGTTATCACAGCCTTGTACAGGCAAGAGTTACTTGTTATCGGCCCTACGGCCTTGCATGGACGGGTGTTGCTTGTTACTGGCTTTGAAGGTTACAGAATTGCTGTGTTAGTTATAAACACAGGAACATGGAAAAATACAGAATGCGACTGAAAGATATAAAAGTCTTGCTGCTTAACCCAATAAACGATTTCGTGTTTACCCTAGCCGGAGTCCATGCCTTCATACACCACAAATGGCGCCCAAATCGGGAATGCAAATCTGAATATATGATCCGGTGGTGTACCCGCAGCTGGACGGAGGATATGAGTTCGTAAAAACTCCGGCCGGAGACGTTAGGACATCACCATCCGCAGGACGGAGAGATGAGCAGCCGTGGAAGAATTGACAGCATGGGGCAATCAGTCACGAGAGAGCAGAAGTTGCAGCTTGAGATTTTGCAACGTATTTTAAAGGAGCAAGGGTTTAAGGTGCAACCCATACCACTGGTCCAGTTATTAGTATGGATTCGGGATCATTGTATATGGTTCCCTGAAGACGGTTCTTATAATCTTACCTTATGGCAAAAGGTGGGCTAGGAGCTACAGACTCGTGACAGCTTGTCCCAGCCCCTCCCCAAAGGGGTACAGATTACGTGGAAAACTGTTTTTACGGCGCTGCGGGCGCTGAATCCCGCTGAATAGGTGCTGCAGGAGGCGCCGGCGACGCGCGACTTGGGGGCAGGGAGTGATCAGTGTCCTTTGAAGTTTTGCCTCCAACAAAAGAATTGGAGCCGGTGTACGTTACTGTTCCGGATCCCGAGGACTTGTATCGGGACCAGGATCATAATGACTCTGGCGATCCTTCTGACTCTGGGACTGCAGACCCGAAGGAGGGATCTGATTTATACCCTCCTCTGGCCCCTCTTACAACTACGGCTGCTGCCGCACCCACAGCAAAGGACCTTTTGCAGACCCAGCGCCTGCAGGCGGTATCCCGTTCGTCGATCGTTCACCCTCCGCCCCTTGCTCCCCCTTTGTCTGCTGCGCCGCCGTATGTATATGCTGTGCCTGCTACGGCGCAGCCTGCCTATACCGGGGCCCTTTCGGGCTGCCCGCAGGAGGTATTGCGCCAAGGGGATGTACAGCTACTGCAAGCGATTCCGGTGGTGTATCTTCCCGGGCGGCTGGCTCGGTATGACTCTCTCCCTTATGAGTTGATTGAGGAGTTGCGGAAAAGTGCTCTAGATTATGGGTTACAGTCGTCCTATACAATGAATCTAATAGTGGCTGTCTCTGAATCGTATGTGATGACACCCCATGATTGGCGTACCTTGTTCCGTTTATTGATCAGTCCGGCCCAGTTTTCTGTTTGGGACTCTGAGAATCGGGAAGCTGTTACCTTGCAAGTTATGGATAATCTTGCTAACAATATTAATCTTGGGGTGGATGAGTTAGTAGGGCAAGGGAAGTTTGCCACGCCCCAAGCACAGGTTCAACTGAACCGGGTGGCCTTTACCCAGGCTGCCTCCTTAACCATGAAAGGCGGTAGTCGTCTGGAAAGGTGCCTCGGACGCTTGGGAATCGGATGTTTTCCAAGTACAGGGATCAACCCAGATTGTGGAATTGGCCGCCGCTGTGCGCGCCTTTGAATTATTTGCATCAGAAGCTCTGAATCTGATAGTAGACTCTGCGTACATCTCGGGTATAGTCCAACGCCTGGAAGGCGCCTTTATTAAGGAAGTGGAGAATCGCCTCTTATTCCAATTACTTTTGCGATTATCGCGCCTGTTAACACAAAGGTCTCACCCGTATTTTGTCGTCCATATCAGATCACGTACGGCATTGCCTGGCCCGCTGACCGAGGGCAATGCTGTAGCGGATGTTTTAACAATGCAGGTCGTACGGCCAAATCTGTTAACACAGGCCCGGCTTTCACACGAGTTTTTCCACCAGAATGCGCGGTCTCTGAGCAAACAATTTTCGCTGACTATTCAACAAGTGCGGGATATTGTTCGCGCTTGTCCTGATTGTCAGCAACTGTCCTCAGTACCCATTCCTGCGGGGGTAAATCCCCGGGGCCTTCGATCCAATGACATTTGGCAATCTGATGTCACTCATATGGCCGAATTTGGTAGACTGTGTTATGTTCATGTTACTGTTGATTCCTTCTCGCATCTTGTTGTAGCCACTGCCCATGCAGGAGAAAAGGCTCATGATGTGGTTCGACATTGGTTGCACTCTTTCGTCATCATGGGGGTCCCGGTTACTGTTAAAACTGACAATGGTCCCGCCTATGTCTCTCGTAGGGTACAACTCTTTCTCCAGGATAGGGGGGTGCGCCATGTTACTGGTATCCCTTATTCCCCTACTGGGCAAGCTGTTGTGAAACGGATGCACCACACGCTTAAAGCTCTGTTGTTTAAACAAAAGAGGGGGAATCCCACGGAAATGACTCCCCAGGAACGGTTATACAAGGCTACATACGTTTTAAATTTTTTAACCTTGTCTGATTTGTCCCTTACGGCAGCACAGCACCACTTTGGACAAGACACTACGGGACCCGAATGGCCCCGTGTGTACTATAGACTTCTTGGGTCAGAACAGTGGCAAGGACCTGTACCTCTGATCACCTGGGGCCGTGGGTACGCCTGTGTTTCTCTGCCTTCCGGCCCCTACTGGCTGCCGGCGCATTGTGTCAAGCCCTTCAAGACAAGGATAGCACAATCTGCGGAGGGCCACCAGGATGGCGAAGACGCGGACTGCGTCCCCTGGTACGGGGCTCAACACGGTCAGAATGTTTATGATCTGGAGCCTGATGGGAAGTCTTTGCCTGCCTACCACGGGGAATGGTCTTTACTGGGCACATATTCTTGATCCACCCATCTTCAAACCTATAACCTGGTGGGATTCTACACCACCTATCGGGAATAATGACACTGAATGGATAGGGGGGGTATGGATGCCCCCGACGATAGACGGAGGATCTAACAATACTAGTTTTTTTGCTCTCAACAATACTGACTTTTTAACTGACCTTCCACCCCTTTGTCTGACAACTAGTAATGACAGCGACATTGGCTGTATTCCGGTGTCACCTCAGCAACACCTGATAGTGAGGGACAAACGTGGAACAAAGAATTACACTTTAATAAGCATCCCGGGTGTGTCTGGAGCATCGTCTTTCCACAACCTGACAGAAGTGGGGATATATACCCCCGACTCGCCCCGTTGTACGGAGGACCCACTCGCAAACAATGCACGGTTAAACTGGCAGCTGTGTCGCGGACTCGGTCCTGATCCTGTTAACCTCAACGGAATTACGGGAACTATTTTGGACTGGGACCCTCATGGGGTTTTGTGGGACCCGGCAAGCAACCACTCTGTTGGGTTCGGCATCCATAACCATAGTGTATCCTGGCATGGCGGTGGGTTGGCTGGGCCTCTTTTACAGTTTTATATGCCCGCCAACTACACCGCCTCCCCATTACATACGGGAATTTGGCGTTTACGCTTCGCGTTTATTAATCGTACCTTATGGAATCTCACCCTGTCTAATCATACTGCTAACGCTACACAGTTACGAACTGATCCAATTATGATTTGCACTTCACATCGCTACGTCTTTGCGATGGCACCAGTGGCTACCATAAACCACTGCAAACATGCTTACTGTATTAACCTGACCAATGTATGGTATGGAAATTGTTTCTCTTCTTTAGATGCACTATGCAACAATCTTACCTTTATATTTTCCCTGCCTCGGCGCCCGGATCTGTGGCTTCCTGTTAACCTTACACGTTTATGGGAAGGAGAGAGTGGTCTAGGTCTTTTTGCGCGAATACTCCAGGAGGAGGTGGATAGCTCTAACAAGCGCCAAAAACGATTCATAGGGTGGTTGATATTTGCACTTGTATCTGCTATTGTCATTCTGGCTTCTGCGACCACTGCCGTCACCTCACTGGCGCAGTCAGTGCAGACGGCGAAAGTGGCAGGGGAAACACTAACCAACGTCATGCAGGAATTTGAGATGCAGGAACGTATTGATGAGGAAATCATGGTGAGATTACAGGCTCTCGAGGCAGCGGTAATCTGGCTCGGGGATCGACAGACAGCCTTAAAGACCCGCCTTTCGTTGCATTGTGACTGGGAGCATATCTCTGGAGGCCTTTGTGTTACCCCCTTGCCCCGCATCCATAGGATACGGTCAAACAACATCTGCAAGGCGCGTTTGACACGTCCTTACAGCACGATGTCCGATCCTTGCATGATCAATTAAAAGGCCAGATTCAGCGGCTGCAGGCATTAACCACTCAGAATGTTCTCGAAACCCTTTAATGGGATATGTCGTGGTTGAATCCGAAAACGTGGTTCACGGGGTTAAATCTGCGCGTCTGGGTATTTGTCGGTATAGCAGGAGCCTTGCTTTTCTTCCTTTTGCTCGTTTCCTATATGACATGCTCGCTTATTAGGGCCAAACGCACGGTTGAAGCTCGTGTGATGGCCACGTTGCTCATTAATGGTATAGTTATAAATAAAGGATGGGGAGATGTGGAGAACAGCAGAGCCAAGTAAACAAGGAACTGAGAGGAGAGCTGTGACCATAAGGCTGGTCTCTGTTATCACAGCCTTGTACAGGCAAGAGTTACTTGTTATCGGCCCTACGGCCTTGCATGGACGGGTGTTGCTTGTTACTGGCTTTGAAGGTTACAGAATTGCTGTGTTAGTTATAAACACAGGAACATGGAAAAATACAGAATGCGACTGAAAGATATAAAAGTCTTGCTGCTTAACCCAATAAACGATTTCGTCCTTACCCTAGCCGGAGTCCGTGCCTTCATACACCACA
